# Supplementary figures and images for: Combined immunoinformatic approaches with computational biochemistry for development of subunit-based vaccine against Lawsonia intracellularis
Source: PLoS One. 2025 Feb 24;20(2):e0314254. doi: 10.1371/journal.pone.0314254 (PMC11849901; doi:10.1371/journal.pone.0314254)

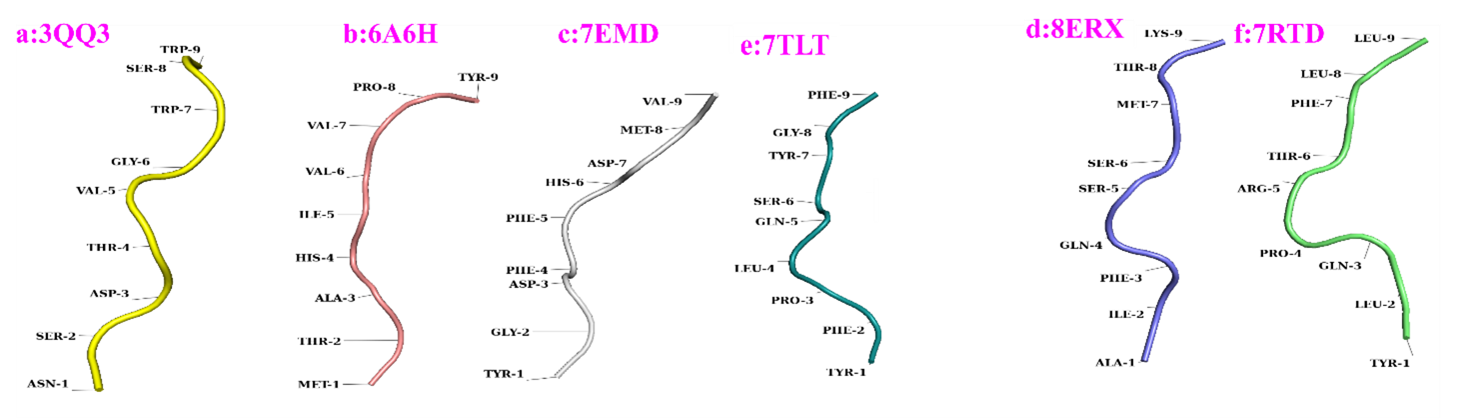

Supplement: S1 Fig — It shows that all epitope obtaining the loop shape while they in interatom with their binding site of the respective MHC receptors. (TIF) [file pone.0314254.s001.tif]

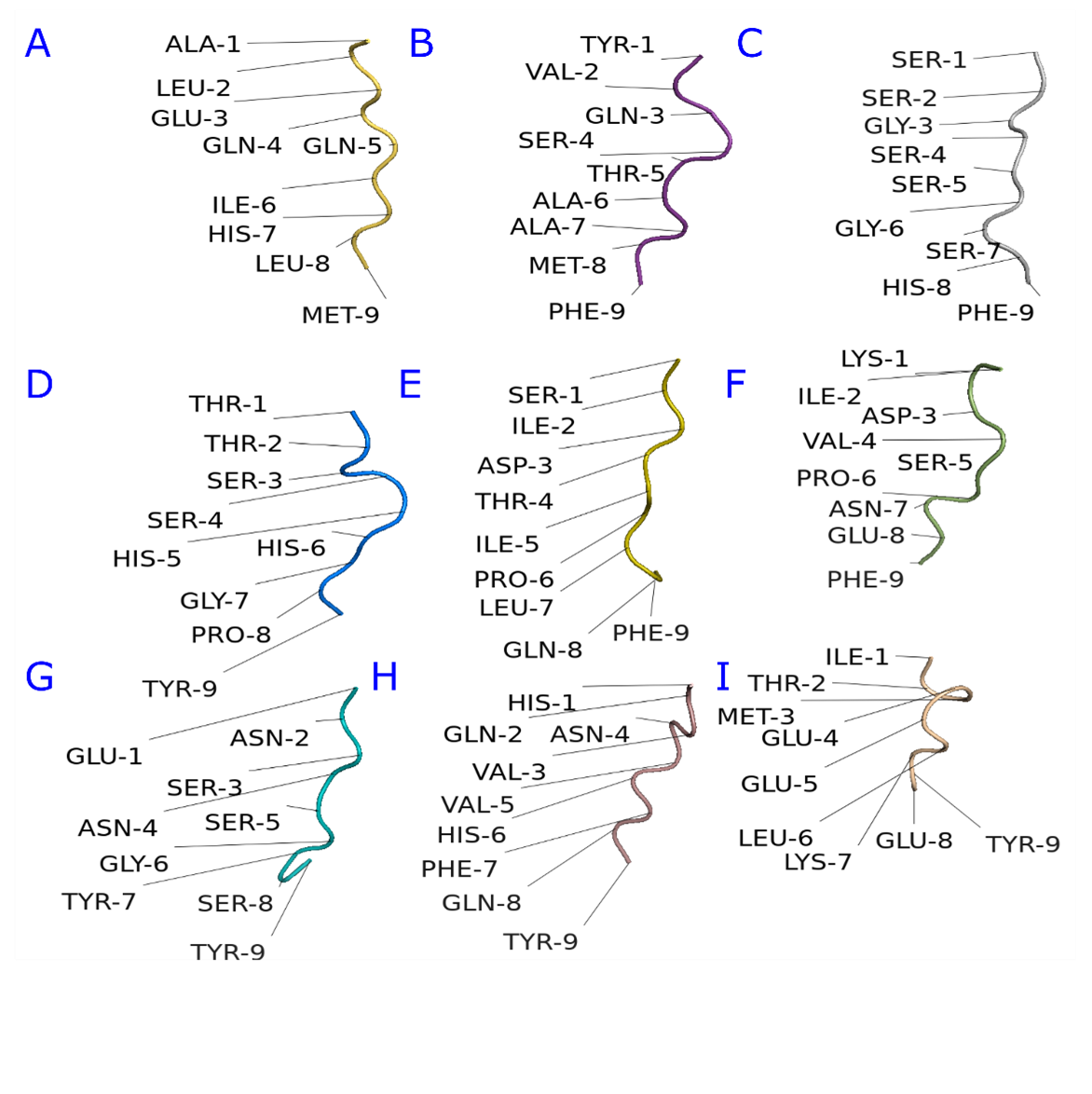

Supplement: S2 Fig — We only show C1-C6 (A-F) and D1-D3 (G-I)., this is applying to all other conformations. (TIF) [file pone.0314254.s002.tif]

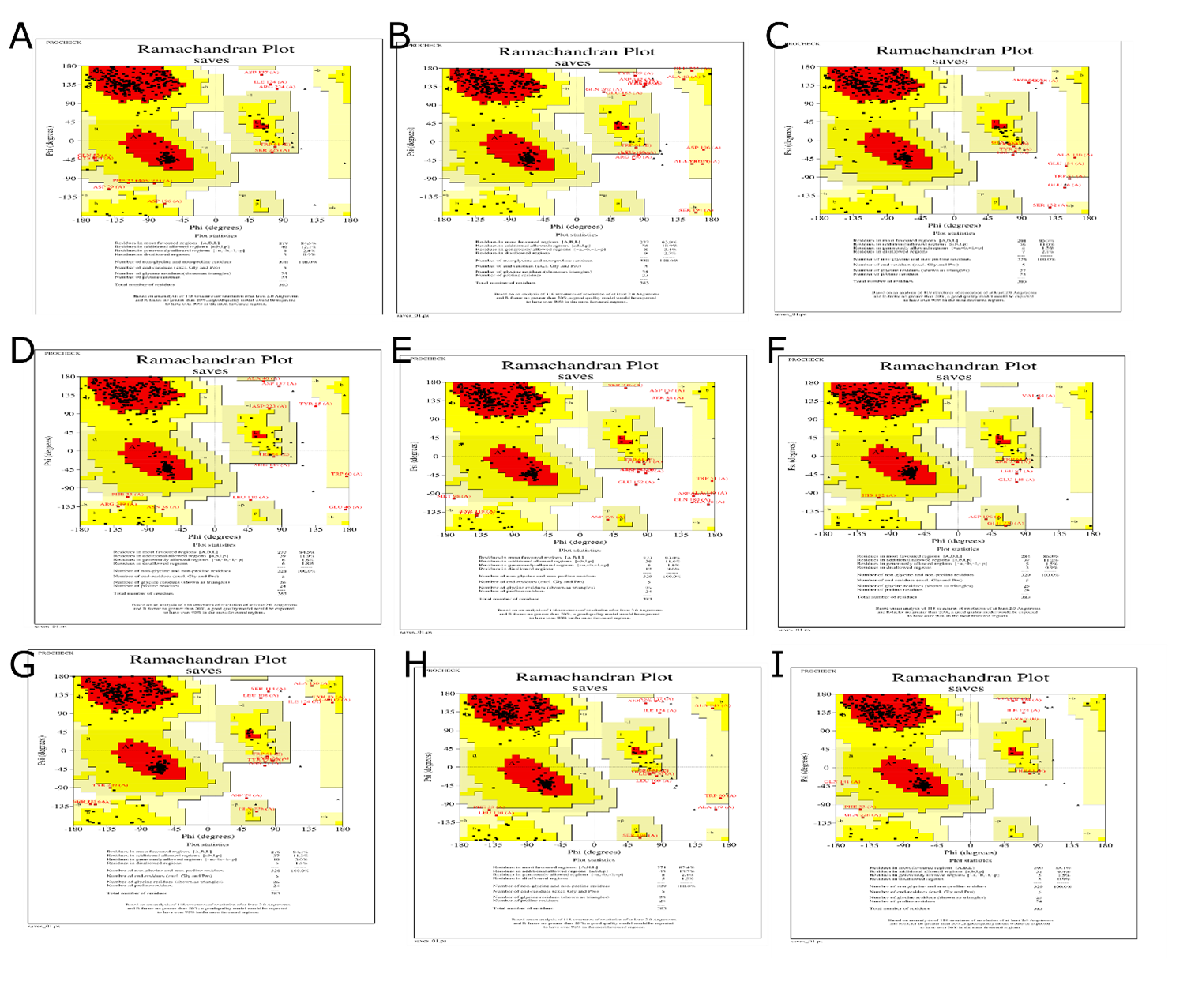

Supplement: S3 Fig — The Ramachandran plot for our epitope-SLA for simulation C1-C6 (A-F) and D1- D3 (G-I). (TIF) [file pone.0314254.s003.tif]
