# Supplementary material for: Combined immunoinformatic approaches with computational biochemistry for development of subunit-based vaccine against Lawsonia intracellularis
Source: PLoS One. 2025 Feb 24;20(2):e0314254. doi: 10.1371/journal.pone.0314254 (PMC11849901; doi:10.1371/journal.pone.0314254)
Supplement: S1 File — S1-S8 Tables are included herein. (DOCX) [file pone.0314254.s004.docx]

**S1 Table**: *Lawsonia intracellularis* proteins (256) were selected based on the pre-prediction criteria.

| **Protein ID** | **Resn** | **HDOC** | **PSI** | **PSI** | **SVM** | **Vaxijen** | **TMHMM** | **Sol** |
| --- | --- | --- | --- | --- | --- | --- | --- | --- |
| Q1MRM5 | 0.8811 | 0.8862 | Virulent | 0.9674 | 0.992 |  |  |  |
| Q1MNY6 | 1.377 | 1.0489 | Virulent | 0.4666 | 0.9151 |  |  |  |
| Q1MSD7 | 1.1605 | 0.8882 | Virulent | 1.5939 | 1.0544 |  | 0 | 0.39 |
| Q1MNU1 | 1.2341 | 1.5363 | Virulent | 0.8119 | 1.0678 |  | 39/595 | 0.227 |
| Q1MSF9 | 1.1305 | 1.2615 | Virulent | 0.4544 | 0.9409 |  |  |  |
| Q1MQR3 | 0.4413 | 0.7229 | Virulent | 0.0634 | 1.0141 | 0.6934 |  |  |
| Q1MP19 | 1.1151 | 0.8065 | Virulent | 0.0401 | 0.861 |  |  |  |
| Q1MQW9 | 1.2082 | 0.7569 | Virulent | 0.8994 | 1.0413 |  |  |  |
| Q1MQF1 | 0.3935 | 0 | Virulent | 0.5074 | 0.9383 |  |  |  |
| Q1MSG6 | 0.7278 | 1.1307 | Virulent | 0.7066 | 1.0407 | 0.674 |  |  |
| Q1MP65 | -0.3 | -0.785 | Virulent | 0.0557 | 1.0831 | 0.6705 | 63/396 | 0.675 |
| Q1MSC6 | 0.9099 | 1.3729 | Virulent | 0.2164 | 0.8157 | 1.1338 | 0 | 0.396 |
| Q1MRZ5 | 0.6086 | 0.5237 | Virulent | 0.3899 | 1.1123 |  |  |  |
| Q1MNU8 | 1.3403 | 0.5187 | Virulent | 1.5222 | 1.0037 |  |  |  |
| Q1MQ36 | 0.6184 | 0.1052 | Non-Virulent | -0.219 | 0.4532 |  |  |  |
| Q1MQB5 | -0.578 | 0.0436 | Non-Virulent | -0.578 | -1.036 |  |  |  |
| Q1MQ50 | -0.795 | -0.768 | Non-Virulent | -0.457 | -0.992 |  |  |  |
| Q1MSD8 | 1.0566 | 0.936 | Virulent | 0.5592 | 0.9846 |  |  |  |
| Q1MP04 | -0.241 | 0.529 | Virulent | 0.283 | -0.732 |  | 0 | 0.737 |
| Q1MQX8 | 0.4183 | 0.4951 | Virulent | 0.2881 | 1.1722 |  |  |  |
| Q1MS31 | 0.3214 | 0.8783 | Virulent | 0.9095 | 1.0325 | 0.6255 |  |  |
| Q1MP76 | 0.8807 | 1.111 | Virulent | 0.8028 | 0.9968 |  |  |  |
| Q1MPV3 | 1.0327 | 0.8947 | Virulent | 1.2628 | 0.101 |  | 22/300 | 0.326 |
| Q1MQF4 | -0.451 | -0.676 | Non-Virulent | -0.881 | -1.018 |  | 113/228 | 0.57 |
| Q1MNS7 | 1.0334 | 0.9085 | Virulent | 1.0908 | 1.0299 |  |  |  |
| Q1MPI1 | 0.9426 | 0.9274 | Virulent | 1.0847 | 1.0096 |  |  |  |
| Q1MP29 | 0.847 | 0.8663 | Virulent | 0.3618 | 0.9707 | 0.6535 |  |  |
| Q1MNT4 | 1.2234 | 1.0718 | Virulent | 0.8416 | 1.0289 | 0.6334 | 32/841 | 0.345 |
| Q1MSA6 | 0.7095 | 0.8513 | Virulent | 0.9174 | 1.0134 |  |  |  |
| Q1MS99 | 0.4622 | 0.4905 | Virulent | 0.4616 | 1.1334 |  |  |  |
| Q1MP32 | -0.068 | 0.6754 | Virulent | 0.73 | 1.1653 |  |  |  |
| Q1MPK7 | 0.9505 | -0.032 | Virulent | 0.1769 | 0.9187 |  |  |  |
| Q1MSB9 | 1.3938 | 2.2132 | Virulent | 1.1324 | 0.8451 |  | 0 | 0.36 |
| Q1MS89 | 0.9918 | 0.6023 | Virulent | 0.1073 | 0.8819 |  |  |  |
| Q1MQF5 | 1.6027 | 1.3111 | Non-Virulent | -0.47 | 0.6328 |  | 0 | 0.449 |
| Q1MNV0 | 0.3616 | 0.4225 | Virulent | 0.2276 | 1.1586 |  |  |  |
| Q1MP51 | 0.6988 | 0.5614 | Virulent | 0.8255 | 1.0014 |  |  |  |
| Q1MQK7 | 0.9533 | 1.7834 | Virulent | 0.9679 | 1.0506 | 0.8921 | 0 | 0.753 |
| Q1MQN5 | -0.516 | -0.069 | Virulent | 0.3119 | -0.459 |  |  |  |
| Q1MPG7 | 0.8974 | 0.4243 | Virulent | 0.3301 | 0.8971 |  |  |  |
| Q1MQZ9 | 1.0261 | -0.017 | Non-Virulent | -0.131 | 0.6316 |  |  |  |
| Q1MNS1 | 0.7785 | 2.3021 | Virulent | 0.6827 | 0.8634 | 0.6224 |  |  |
| Q1MNX9 | 0.3454 | 0.3606 | Non-Virulent | -0.083 | 0.8207 |  |  |  |
| Q1MPE3 | 1.2625 | 0.6216 | Virulent | 0.4319 | 0.9398 |  |  |  |
| Q1MQW1 | 0.585 | 0.2574 | Virulent | 0.7621 | 1.0123 |  |  |  |
| Q1MSE1 | 1.2575 | 0.6283 | Virulent | 1.124 | 1.0373 | 0.9882 | 42/162 | 0.525 |
| Q1MSG3 | 0.8097 | 0.5048 | Virulent | 0.4938 | 1.0468 | 0.9392 | 52/89 | 0.505 |
| Q1MNQ9 | 0.0675 | 0.3965 | Virulent | 0.072 | 0.8563 |  |  |  |
| Q1MP16 | 0.3314 | 0.0101 | Virulent | 0.4781 | 0.895 |  |  |  |
| Q1MSC8 | 0.7582 | 1.9379 | Virulent | 0.6791 | 0.9637 | 0.7067 |  |  |
| Q1MP64 | 1.2407 | 0.4344 | Virulent | 1.17 | 1.014 | 0.7622 | 9/256 | 0.686 |
| Q1MQ46 | 0.9699 | 0.698 | Virulent | 0.3774 | 1.0264 |  |  |  |
| Q1MNP8 | 0.4932 | 0.4735 | Virulent | 0.3792 | 1.1574 |  |  |  |
| Q1MQD0 | 0.8946 | 0.0112 | Virulent | 1.2207 | -0.046 | 0.71 | 0 | 0.498 |
| Q1MPK6 | 0.3553 | 0.1691 | Virulent | 0.6419 | 0.9748 |  |  |  |
| Q1MRC6 | 1.0098 | 0.5367 | Virulent | 0.2197 | 1.0227 |  |  |  |
| Q1MRH6 | -0.219 | 0.45 | Virulent | 0.5393 | 0.9844 |  |  |  |
| Q1MNS3 | 0.7537 | 2.1324 | Virulent | 0.4416 | 0.8275 | 0.6224 |  |  |
| Q1MP07 | -0.207 | -0.763 | Non-Virulent | -0.532 | -1.085 |  |  |  |
| Q1MQ09 | 0.5797 | 0.4443 | Virulent | 0.5291 | 1.0657 | 0.8035 |  |  |
| Q1MP48 | 1.1803 | 1.0304 | Virulent | 0.8233 | 1.0579 |  |  |  |
| Q1MPH4 | 1.006 | 0.9636 | Virulent | 1.1175 | 1.0615 |  |  |  |
| Q1MR66 | 0.3472 | 0.362 | Non-Virulent | -0.153 | 0.9826 |  |  |  |
| Q1MNN5 | 0.7255 | 1.4519 | Virulent | 0.9243 | 1.0739 |  |  |  |
| Q1MNX4 | -0.622 | -0.462 | Non-Virulent | -0.721 | -0.98 |  |  |  |
| Q1MS97 | 0.5419 | 1.6099 | Virulent | 0.6396 | 0.9619 | 0.972 | 0 | 0.677 |
| Q1MRN0 | 0.1905 | 0.1073 | Non-Virulent | -0.327 | -0.505 | 0.6587 |  |  |
| Q1MQN9 | 1.3423 | 0.4075 | Virulent | 0.6399 | 0.9769 |  |  |  |
| Q1MR99 | 1.0208 | 1.0963 | Virulent | 0.1522 | 0.7283 |  |  |  |
| Q1MS71 | 0.3046 | -0.055 | Virulent | 0.8712 | 0.9832 | 0.7138 |  |  |
| Q1MRY6 | 0.7455 | 0.2533 | Virulent | 0.1889 | 0.9987 |  |  |  |
| Q1MNT1 | 1.1086 | 1.7288 | Virulent | 0.9675 | 1.0413 | 0.6725 | 83/688 | 0.221 |
| Q1MPI9 | 0.1002 | 0.0796 | Virulent | 0.2178 | 0.7366 |  |  |  |
| Q1MP18 | 1.5369 | 0.8597 | Virulent | 0.748 | 1.0209 |  |  |  |
| Q1MQ33 | 0.7707 | 1.1502 | Virulent | 0.7545 | 1.076 |  |  |  |
| Q1MQ61 | 1.2775 | 0.4534 | Virulent | 0.7398 | 1.0137 |  |  |  |
| Q1MS98 | 0.8397 | 1.0235 | Virulent | 0.7048 | 1.0651 | 1.0041 | 0 | 0.751 |
| Q1MNU0 | 0.8857 | 2.0342 | Virulent | 0.7551 | 0.8726 | 0.6539 |  |  |
| Q1MPI2 | 1.2298 | 1.8109 | Virulent | 0.9536 | 1.0527 |  |  |  |
| Q1MQM4 | 0.9955 | 1.2973 | Virulent | 1.0481 | 1.1157 |  |  |  |
| Q1MP13 | 0.5262 | 0.5587 | Virulent | 0.1602 | 1.1615 | 0.9965 | 55/110 | 0.624 |
| Q1MSD9 | 0.3542 | 0.7826 | Virulent | 0.3469 | 1.0295 |  |  |  |
| Q1MQX6 | 1.2129 | 1.1129 | Non-Virulent | -0.282 | 0.2604 |  |  |  |
| Q1MQZ8 | -0.352 | 0.0165 | Virulent | 0.6149 | 0.5032 |  |  |  |
| Q1MNT9 | 1.3062 | 2.3258 | Virulent | 0.9402 | 0.8963 |  |  |  |
| Q1MRR7 | 0.7732 | 0.3986 | Non-Virulent | -0.301 | 0.6886 |  |  |  |
| Q1MNU7 | 0.4468 | 0.5042 | Non-Virulent | -0.108 | 0.6818 |  |  |  |
| Q1MNT7 | 1.452 | 1.8926 | Virulent | 1.2382 | 1.0582 |  |  |  |
| Q1MRZ4 | 1.139 | 0.9138 | Virulent | 0.7689 | 1.0247 |  |  |  |
| Q1MP67 | 1.0871 | 0.6698 | Virulent | 1.1398 | 1.0477 |  |  |  |
| Q1MQ11 | 0.6437 | 1.0034 | Virulent | 0.31 | 1.0129 |  |  |  |
| Q1MQK6 | -0.06 | -0.14 | Virulent | 0.7646 | 0.7078 | 0.8813 | 0 | 0.51 |
| Q1MNQ5 | 0.2676 | 0.0567 | Virulent | 0.5155 | 0.9095 | 0.7986 |  |  |
| Q1MPX4 | 1.3408 | 0.6633 | Virulent | 1.3125 | 1.063 | 0.7516 | 0 | 0.731 |
| Q1MRR5 | 0.9372 | 0.8632 | Virulent | 0.873 | 1.0678 |  |  |  |
| Q1MNT8 | 1.5965 | 1.9513 | Virulent | 0.9887 | 0.9215 |  |  |  |
| Q1MPR6 | -0.084 | 0.2811 | Virulent | 0.4913 | 0.3298 |  |  |  |
| Q1MNU6 | 1.1935 | 1.7653 | Virulent | 1.4239 | 1.0115 | 0.6462 |  |  |
| Q1MPD5 | 0.3903 | 0.0466 | Non-Virulent | -0.021 | 0.7802 |  |  |  |
| Q1MQR2 | 1.0695 | 1.348 | Virulent | 0.2999 | 0.7111 |  |  |  |
| Q1MNS8 | 1.323 | 1.1711 | Virulent | 1.028 | 1.1033 | 0.801 | 22/702 | 0.236 |
| Q1MPI7 | 1.7176 | 0.8123 | Virulent | 1.0028 | 1.0539 |  |  |  |
| Q1MP78 | 0.6655 | 0.7408 | Virulent | 0.4249 | 1.0395 | 0.8538 | 0 | 0.725 |
| Q1MNS9 | 1.144 | 0.4893 | Virulent | 0.9232 | 0.9929 | 0.6333 |  |  |
| Q1MP44 | 1.3791 | 0.9318 | Virulent | 0.1912 | 0.3391 |  | 0 | 0.518 |
| Q1MNY4 | 0.8228 | 0.107 | Virulent | 0.6379 | 0.9565 |  |  |  |
| \|Q1MP22 | 1.4862 | 0.694 | Virulent | 0.9039 | 1.0545 |  |  |  |
| Q1MNT0 | 1.0705 | 0.8531 | Virulent | 0.9586 | 1.036 | 0.6947 |  |  |
| Q1MPY3 | 1.3315 | 2.1612 | Virulent | 1.5676 | 0.9015 | 0.6353 | 41/1340 | 0.546 |
| Q1MQ75 | 0.5033 | -0.287 | Non-Virulent | -0.866 | -0.684 | 0.7691 |  |  |
| Q1MNR0 | -0.934 | -0.64 | Virulent | 0.3606 | -0.854 | 0.6923 |  |  |
| Q1MPY6 | 1.3867 | 1.7095 | Virulent | 0.8807 | 1.0479 |  |  |  |
| Q1MR59 | 0.7006 | 0.7468 | Non-Virulent | -0.387 | 0.1334 |  |  |  |
| Q1MQV8 | 0.8241 | 1.2941 | Virulent | 0.5181 | 0.945 |  |  |  |
| >tr\|Q1MR02 | 0.1352 | 0.3201 | Virulent | 0.6406 | 1.0989 |  |  |  |
| Q1MNN1 | 0.0364 | 0.1097 | Virulent | 0.0844 | 0.3254 | 0.7941 | 85/136 | 0.608 |
| Q1MPI4 | 1.1099 | 0.3148 | Virulent | 0.703 | 0.9764 |  |  |  |
| \|Q1MQW0 | 1.0361 | 0.9705 | Virulent | 0.2432 | 0.9123 | 0.617 |  |  |
| Q1MRZ2 | 0.9247 | 1.0226 | Virulent | 1.3705 | 1.0313 | 0.6050 | 0 | 0.712 |
| Q1MPS4 | 0.3041 | 0.1917 | Virulent | 0.519 | 1.0613 | 0.78 |  |  |
| Q1MRL1 | 0.6624 | 0.0555 | Virulent | 0.2202 | 0.9054 |  |  |  |
| Q1MP39 | 1.3022 | 1.1269 | Virulent | 0.7445 | 1.0637 |  |  |  |
| Q1MSD0 | 0.8807 | 0.5897 | Virulent | 0.4422 | 1.0735 |  |  |  |
| Q1MS27 | 0.8381 | -0.098 | Virulent | 0.5653 | 0.9367 |  |  |  |
| Q1MSC7 | 0.7699 | 0.2042 | Non-Virulent | -0.126 | 0.785 |  |  |  |
| Q1MNP2 | 0.6552 | 0.0822 | Non-Virulent | -0.116 | 0.6933 |  |  |  |
| Q1MNX2 | 0.1252 | 0.2465 | Non-Virulent | -0.269 | 0.3189 |  |  |  |
| Q1MPN1 | 1.0265 | 0.4289 | Virulent | 0.3219 | 0.9507 |  |  |  |
| Q1MNP4 | 0.2046 | 0.3067 | Non-Virulent | -0.352 | 0.1117 |  |  |  |
| Q1MQJ6 | 0.2913 | 0.4187 | Virulent | 0.3509 | 1.1383 |  |  |  |
| Q1MNT3 | 0.3296 | 1.1594 | Virulent | 0.5094 | 0.8607 | 0.8226 | 0 | 0.578 |
| Q1MP61 | 0.8708 | 0.5086 | Virulent | 0.6507 | 1.0005 |  |  |  |
| Q1MPI6 | -0.128 | -0.089 | Non-Virulent | -1.076 | -1.004 |  |  |  |
| Q1MNR6 | 1.0262 | 1.1586 | Virulent | 0.5207 | 0.1163 | 0.7515 | 191/1139 | 0.3 |
| Q1MP62 | -0.107 | -0.695 | Virulent | 0.3767 | 0.9291 |  |  |  |
| Q1MS34 | 0.5 | 0.1665 | Virulent | 0.1786 | 1.078 |  |  |  |
| Q1MNW2 | 0.4241 | 0.0433 | Non-Virulent | -0.068 | 0.7452 |  |  |  |
| Q1MP27 | 0.7964 | 0.6725 | Virulent | 0.7374 | 0.9931 | 0.6542 |  |  |
| Q1MS65 | 0.8981 | 0.1573 | Non-Virulent | -0.371 | -0.119 |  |  |  |
| Q1MP85 | -0.119 | -0.773 | Non-Virulent | -0.122 | -0.767 |  |  |  |
| Q1MQK5 | 0.842 | 1.4637 | Virulent | 0.8151 | 0.93 |  |  |  |
| Q1MP02 | 0.0828 | -0.366 | Non-Virulent | -0.302 | -0.905 |  |  |  |
| Q1MPI5 | 0.8677 | 1.7278 | Virulent | 0.7948 | 0.9655 |  |  |  |
| Q1MRJ3 | -0.067 | -0.241 | Virulent | 0.0109 | -0.592 | 0.6958 |  |  |
| Q1MRM3 | 0.7081 | 0.5417 | Virulent | 0.9476 | 0.9794 |  |  |  |
| Q1MRY7 | -0.603 | 0.1026 | Non-Virulent | -0.898 | -1.038 |  |  |  |
| Q1MPE6 | 1.0069 | 1.7694 | Virulent | 1.0547 | 1.0433 | 0.8743 | 0 | 0.705 |
| Q1MR75 | 0.9553 | 0.9041 | Virulent | 0.0951 | 0.8385 |  |  |  |
| Q1MSG1 | 0.913 | 0.7262 | Virulent | 0.4836 | 1.0283 |  |  |  |
| Q1MRX9 | 0.9213 | 0.2796 | Virulent | 0.5313 | 0.928 |  |  |  |
| Q1MRJ2 | 0.4526 | 0.4994 | Non-Virulent | -0.549 | -0.055 |  |  |  |
| Q1MP31 | 0.909 | 0.8526 | Virulent | 0.8021 | 1.055 |  |  |  |
| Q1MRZ9 | -0.163 | -0.018 | Non-Virulent | -0.099 | -0.55 |  |  |  |
| Q1MSB6 | 0.8654 | 1.1046 | Virulent | 0.5266 | 0.9873 | 0.8246 | 22/110 | 0.588 |
| Q1MRZ3 | 1.3351 | 0.9184 | Virulent | 0.5269 | 0.9272 |  |  |  |
| Q1MNW1 | 0.9242 | 0.8617 | Virulent | 0.9015 | 0.9914 |  |  |  |
| Q1MQD9 | 0.0707 | 0.1018 | Non-Virulent | -0.642 | -0.515 |  |  |  |
| Q1MNT2 | 0.3365 | 0.1973 | Non-Virulent | -0.088 | 0.7708 |  |  |  |
| Q1MNR7 | 0.8358 | 1.0528 | Virulent | 1.046 | 1.0904 |  |  |  |
| Q1MP58 | 0.4921 | 0.3087 | Virulent | 0.7287 | 1.0694 | 0.8789 | 0 | 0.893 |
| Q1MQX9 | -1.385 | -0.449 | Non-Virulent | -1.146 | -0.927 |  |  |  |
| Q1MQX7 | 0.4618 | 0.0297 | Virulent | 0.8421 | 1.0152 |  |  |  |
| Q1MNZ9 | 1.5199 | 1.0704 | Virulent | 1.3219 | 1.1161 |  | 12/723 | 0.33 |
| Q1MPJ0 | 1.5521 | 0.99 | Virulent | 1.2387 | 1.0867 |  |  |  |
| Q1MPI8 | 0.7644 | 1.0388 | Virulent | 0.7187 | 1.0016 |  |  |  |
| Q1MRS2 | 1.4556 | 1.6749 | Virulent | 1.2253 | 1.1016 | 0.7219 | 0 | 0.802 |
| Q1MQK9 | 1.5011 | 1.5471 | Virulent | 1.4714 | 1.034 |  |  |  |
| Q1MNN9 | 0.5746 | -0.348 | Virulent | 0.5278 | 0.5679 |  |  |  |
| Q1MQ70 | 1.312 | 0.8534 | Virulent | 0.35 | 0.9079 |  |  |  |
| Q1MR57 | 0.2408 | -0.147 | Non-Virulent | -0.177 | -0.085 |  |  |  |
| Q1MS26 | 1.2424 | 0.7956 | Virulent | 0.7664 | 1.0424 |  |  |  |
| Q1MNZ1 | 1.0613 | 0.9018 | Virulent | 0.3129 | 0.9239 |  |  |  |
| Q1MQK8 | 1.3482 | 1.2102 | Virulent | 1.2035 | 1.1042 | 0.7395 | 0 | 0.643 |
| Q1MSF7 | 0.7073 | 0.1476 | Virulent | 0.0712 | 1.0539 |  |  |  |
| Q1MQK4 | 0.7591 | 1.0158 | Virulent | 0.6464 | 0.9939 |  |  |  |
| Q1MSE0 | -0.871 | -0.195 | Non-Virulent | -0.418 | -1.037 |  |  |  |
| Q1MNR5 | 0.1882 | 0.433 | Non-Virulent | -0.029 | 0.8445 |  |  |  |
| Q1MQA4 | 0.2062 | 0.0761 | Virulent | 0.2696 | 0.8375 |  |  |  |
| Q1MRW9 | 1.2234 | 0.9248 | Virulent | 0.579 | 0.9865 |  |  |  |
| Q1MP68 | 0.2736 | 1.2466 | Virulent | 0.899 | 0.9652 | 0.766 | 0 | 0.542 |
| Q1MNS0 | 0.8448 | 0.4796 | Virulent | 0.3302 | 0.9415 | 0.7393 |  |  |
| Q1MSC4 | 1.1291 | 0.8392 | Virulent | 0.7935 | 1.0052 |  |  |  |
| Q1MNU2 | 1.1136 | 1.0761 | Virulent | 0.1385 | 0.7656 |  |  |  |
| Q1MQE6 | 0.7374 | 0.1066 | Virulent | 0.067 | 1.0282 |  |  |  |
| Q1MQY1 | -0.059 | -0.8 | Non-Virulent | -0.476 | -0.87 |  |  |  |
| Q1MNN2 | 1.0677 | 1.9201 | Virulent | 1.023 | 0.9332 |  |  |  |
| Q1MRZ0 | 0.2907 | -0.312 | Non-Virulent | -0.624 | -0.873 | 0.6732 |  |  |
| Q1MNQ8 | 1.3908 | 2.1089 | Virulent | 1.3797 | 1.0059 |  | 0 | 0.697 |
| Q1MSA0 | 0.547 | 1.626 | Virulent | 0.3014 | 0.7767 | 0.8174 | 0 | 0.586 |
| Q1MQL0 | 1.1625 | 0.2459 | Virulent | 0.2837 | 0.9838 |  |  |  |
| Q1MS96 | 0.4412 | 1.268 | Virulent | 0.3813 | 0.9694 | 0.8588 | 0 | 0.609 |
| Q1MNZ2 | -0.015 | -0.293 | Non-Virulent | -0.162 | -0.379 |  |  |  |
| Q1MP82 | 0.93 | 0.5544 | Virulent | 0.5057 | 1.0358 |  |  |  |
| Q1MRQ3 | 1.0306 | 0.7178 | Virulent | 0.5328 | 0.9705 | 0.6514 |  |  |
| Q1MSC9 | 1.3636 | 1.6317 | Virulent | 1.3083 | 1.0581 |  |  |  |
| Q1MPM3 | 0.6231 | 0.5429 | Virulent | 0.9582 | 1.011 |  |  |  |
| Q1MP14 | 0.9937 | 0.4456 | Non-Virulent | -0.043 | 0.8699 | 1.0917 | 65/124 | 0.459 |
| Q1MNS4 | 1.242 | 1.64 | Virulent | 0.7414 | 1.0266 |  | 19/700 | 0.311 |
| Q1MQ76 | 0.8852 | 0.5823 | Virulent | 0.0978 | 1.05 |  |  |  |
| Q1MNM7 | 0.6657 | 0.7976 | Virulent | 0.0316 | 0.9778 |  |  |  |
| Q1MQ64 | 0.8751 | 0.5714 | Virulent | 0.7884 | 0.9923 |  |  |  |
| Q1MP28 | 1.0839 | 1.1388 | Virulent | 0.875 | 1.1048 |  |  |  |
| Q1MNS6 | 1.5811 | 1.3161 | Virulent | 1.3551 | 1.0977 | 0.7132 | 19/619 | 0.303 |
| Q1MS54 | 1.0374 | 0.9692 | Virulent | 0.8355 | 1.0486 |  |  |  |
| Q1MRY4 | -0.263 | -0.545 | Non-Virulent | -0.722 | -0.797 |  |  |  |
| Q1MP71 | 1.5582 | 0.8647 | Virulent | 0.3021 | 0.81 |  |  |  |
| Q1MSG0 | 0.548 | 0.3501 | Virulent | 1.1723 | 1.0115 |  |  |  |
| Q1MPL5 | 1.108 | 0.853 | Virulent | 0.8365 | 0.9992 |  |  |  |
| Q1MNV6 | -0.019 | 0.6759 | Virulent | 0.1683 | 0.4045 |  |  |  |
| Q1MNW6 | -0.184 | 0.3272 | Non-Virulent | -0.229 | -0.344 |  |  |  |
| Q1MP75 | 0.0606 | 0.4388 | Virulent | 0.0687 | 0.8993 | 0.8044 |  |  |
| Q1MNS2 | 1.4342 | 1.8702 | Virulent | 1.2031 | 1.0629 |  | 0 | 0.264 |
| Q1MS66 | 0.6675 | 1.0141 | Virulent | 0.9013 | 1.0228 |  |  |  |
| Q1MNV9 | 0.4331 | -0.027 | Non-Virulent | -0.004 | 0.7489 | 0.6608 |  |  |
| Q1MPA5 | -1.504 | -1.253 | Non-Virulent | -1.073 | -1.095 |  |  |  |
| Q1MP38 | -0.76 | -0.236 | Non-Virulent | -1.015 | -0.994 |  |  |  |
| Q1MNW0 | 0.0821 | -0.339 | Non-Virulent | -0.119 | -0.577 | 0.7379 |  |  |
| Q1MQ69 | -0.346 | 0.0996 | Non-Virulent | -0.021 | -0.443 |  |  |  |
| Q1MSB0 | 0.1534 | 0.0324 | Non-Virulent | -0.394 | -0.443 |  |  |  |
| Q1MRZ8 | 1.125 | 1.129 | Virulent | 0.6151 | 0.9456 | 0.7009 | 0 | 0.296 |
| Q1MPI3 | 1.3654 | 1.9972 | Virulent | 1.3457 | 0.8686 |  | 0 | 0.516 |
| Q1MNR9 | 1.0224 | 0.875 | Virulent | 0.113 | 0.7895 |  |  |  |
| Q1MNZ0 | 1.2971 | 0.7475 | Virulent | 0.2653 | 0.9191 |  |  |  |
| Q1MNT5 | 1.4408 | 2.0907 | Virulent | 0.9827 | 0.9404 |  |  |  |
| Q1MRP8 | 0.9116 | 0.1169 | Virulent | 0.7201 | 1.0034 |  |  |  |
| Q1MP30 | 1.0398 | 0.2639 | Virulent | 0.7444 | 0.9901 | 0.6739 |  |  |
| Q1MRR1 | 0.7916 | 0.7763 | Virulent | 0.9016 | 0.9966 | 0.7236 |  |  |
| Q1MSA8 | 0.8953 | 1.1604 | Virulent | 0.9946 | 1.1059 | 0.6918 | 0 | 0.739 |
| Q1MQD4 | 0.9194 | 2.1663 | Virulent | 1.2101 | 0.8111 |  | 21/230 | 0.49 |
| Q1MPF5 | 0.5847 | -0.282 | Virulent | 0.096 | 0.2792 |  |  |  |
| Q1MS36 | 0.6114 | 0.6676 | Virulent | 0.7886 | 1.0128 |  |  |  |
| Q1MP69 | 0.9785 | 1.936 | Virulent | 1.2652 | 0.977 | 0.6818 | 0 | 0.496 |
| Q1MS85 | 0.9956 | 0.1844 | Virulent | 0.3985 | 0.9858 |  |  |  |
| Q1MNT6 | 0.7696 | 1.2857 | Non-Virulent | -0.162 | 0.5532 |  |  |  |
| CAJ54196.1 | 1.4492 |  |  |  |  | 0 |  | 0.14 |
| CAJ55049.1 |  |  |  |  |  | 0.429 |  | 0.16 |
| CAJ54898.1 | 1.0898 |  |  |  |  | 0 |  | 0.25 |
| CAJ54431.1 |  |  |  |  |  | 0 |  | 0.27 |
| CAJ54357.1 | 0.95 |  |  |  |  | 0 |  | 0.33 |
| CAJ54789.1 | 1.4895 |  |  |  |  | 0.48 |  | 0.33 |
| CAJ55230.1 | 0.9066 |  |  |  |  | 0.418 |  | 0.34 |
| CAJ54417.1 |  |  |  |  |  | 0 |  | 0.37 |
| CAJ54091.1 | 1.3123 |  |  |  |  | 0 |  | 0.37 |
| CAJ54672.1 |  |  |  |  |  | 0.414 |  | 0.38 |
| CAJ54934.1 |  |  |  |  |  | 0 |  | 0.4 |
| CAJ54315.1 |  |  |  |  |  |  |  | 0.49 |
| CAJ54315.1 | 0.7916 |  |  |  |  | 0.723 |  | 0.49 |
| CAJ54808.1 |  |  |  |  |  | 0.45 |  | 0.5 |
| CAJ54511.1 |  |  |  |  |  | 0.42 |  | 0.58 |
| CAJ54428.1 | 0.8262 |  |  |  |  | 0 |  | 0.53 |
| CAJ54496.1 |  |  |  |  |  | 0 |  | 0.55 |
| CAJ55195.1 | 0.93 |  |  |  |  | 0 |  | 0.6 |
| CAJ54302.1 | 1.0503 |  |  |  |  | 0.416 |  | 0.67 |
| CAJ54210.1 |  |  |  |  |  | 0.476 |  | 0.67 |
| CAJ54281.1 |  |  |  |  |  | 0.431 |  | 0.68 |
| Proteins from pathogenic LI and LI proteins with unknown function assessed based on virulency, antigenicity, solubility and membrane helicity. Abbreviation used in the table. Resn: Residue composition. HODC: Higher order Dipeptide Composition Based. PSI-BLAST-PSSM: PSI-BLASTcreated PSSM Profiles. SB: Similarity based. SVMs and PSI-BLAST: Cascade of SVMs and PSI-BLAST (All based on VirulentPred). Sol: solubility prediction-based Protein-Sol. Antigenicity: ANTIGENpro based antigenicity detection. TMHMM: Transmembrane helices prediction based on TMHMM — 2.0. | | | | | | | | |

**S2 Table**: Outputs of prediction for 19 pre-prediction proteins after assessments of their ranked, digestion, signal peptide and toxicity.

| **Uniprot Entry** | **Epi** | **EL_rank** | **Antigenicity** | **Selected** | | **Digestion** | **S-P** | **Docked** | **Prediction** | **Charge** | **Mol Wt** |
| --- | --- | --- | --- | --- | --- | --- | --- | --- | --- | --- | --- |
| Q1MNQ8_a2 | QLAPTPLLY | 0.0214 | 1.1814 | Yes | | Yes | No | Y | Non-Toxin | 0 | 1015.35 |
| Q1MNQ8_a3 | ALEQQIHLM | 0.0704 | 0.6691 |  | | No | No | Y | Non-Toxin | -0.5 | 1082.43 |
| Q1MNQ8_a4 | QTQNTNTLF | 0.1171 | 0.4484 |  | | No | No | Y | Non-Toxin | 0 | 1066.27 |
| Q1MNQ8_a5 | TTNSQHPLF | 0.149 | 0.1511 |  | | No | No | Y | Non-Toxin | 0.5 | 1044.26 |
| Q1MNQ8_a6 | ASFINTETY | 0.2192 | -0.2724 |  | | 3/6chy | No | Y | Non-Toxin | -1 | 1045.23 |
| Q1MNQ8_a7 | STSMDGSGY | 0.2285 | 1.872 |  | |  | No | Y | Non-Toxin | -1 | 904.03 |
| Q1MNQ8_a8 | SMIGNNIDY | 0.2472 | 0.1810 |  | |  | No | Y | Non-Toxin | -1 | 1026.26 |
| Q1MNQ8_a9 | TSDRDKENF | 0.3668 | 0.8785 |  | |  | No | Y | Non-Toxin | -1 | 1111.24 |
| Q1MNQ8_a10 | VLGEFFERY | 0.385 | -0.0209 |  | | Several | No | Y | Non-Toxin | -1 | 1159.43 |
| Q1MNQ8_sla2_a1 | SRLSPYKEY | 0.0914 | -0.1861 |  | | Several | No | Y | Non-Toxin | 1 | 1142.39 |
| Q1MNQ8_sla2_a2 | ASFINTETY | 0.1438 | -0.2724 |  | | 3/6chy | No | Y | Non-Toxin | -1 | 1045.23 |
| Q1MNQ8_sla2_a3 | QLAPTPLLY | 0.1469 | 1.1814 | X | | | No | Y | Non-Toxin | 0 | 1015.35 |
| Q1MNQ8_sla2_a4 | IERVVAKQY | 0.2285 | 0.0630 |  | |  | No | Y | Non-Toxin | 1 | 1105.43 |
| Q1MNQ8_sla2_a5 | ERVVAKQYF | 0.3034 | 0.5203 |  | |  | No |  | Non-Toxin | 1 | 1139.44 |
| Q1MP64_a1 | YVQSTAAMF | 0.1966 | -0.0353 |  | | 1.00 | No | Y | Non-Toxin | 0 | 1017.28 |
| Q1MP64_a2 | SVMTTAMGF | 0.2095 | 0.2473 |  | |  | No | Y | Non-Toxin | 0 | 944.25 |
| Q1MP64_a3 | ALEGMVNKM | 0.3171 | 0.5392 | Yes | | Several | No | Y | Non-Toxin | 0 | 992.35 |
| Q1MP64_a4 | AQMELVKQF | 0.4285 | -0.0172 |  | |  | No |  | Non-Toxin | 0 | 1093.44 |
| Q1MP64_a5 | LSAPKGGGY | 0.7445 | 0.5353 |  | | Several | No |  | Non-Toxin | 1 | 849.08 |
| Q1MP64_a6 | AQDVQNSAL | 0.7767 | 0.5891 |  | | 7—2 | No |  | Non-Toxin | -1 | 945.12 |
| Q1MP64_a7 | KVDGVSIQA | 1.0113 | 1.9933 | Yes | | Several | No |  | Non-Toxin | 0 | 916.17 |
| Q1MP64_a8 | ASSVMTTAM | 1.0179 | 0.616 |  | |  | No |  | Non-Toxin | 0 | 898.17 |
| Q1MP64_a9 | SLNNSSIQI | 1.2005 | 1.0098 |  | |  | No |  | Non-Toxin | 0 | 975.2 |
| Q1MP64_a10 | GTIGGVKSM | 1.3832 | 0.6653 |  | |  | No |  | Non-Toxin | 1 | 849.15 |
| Q1MP64_sla2_a1 | AQMELVKQF | 0.0284 | -0.0172 |  | | 2.00 | No | Y | Non-Toxin | 0 | 1093.44 |
| Q1MP64_sla2_a2 | EEKKLEAQM | 0.5921 | 1.4551 | X | | 5.00 | No | Y | Non-Toxin | -1 | 1105.4 |
| Q1MP64_sla2_a3 | LEKTIMISL | 0.6803 | 0.1038 |  | |  | No | Y | Non-Toxin | 0 | 1047.46 |
| Q1MP64_sla2_a4 | EQSKLMMGL | 1.0371 | 0.0598 |  | |  | No |  | Non-Toxin | 0 | 1036.41 |
| Q1MP64_sla2_a5 | SVMTTAMGF | 1.3364 | 0.2473 |  | |  | No | Y | Non-Toxin | 0 | 944.25 |
| Q1MS98_a1 | SSGSSGSHF | 0.393 | 1.7727 | Yes | | No | No | Y | Non-Toxin | 0.5 | 851.94 |
| Q1MS98_a2 | ATDDNDKNL | 0.4658 | 0.9606 |  | | Several |  |  | Non-Toxin | -2 | 1005.12 |
| Q1MS98_a3 | GVRKGIEAY | 0.6067 | 0.6905 |  | | Several | No | Y | Non-Toxin | 1 | 992.27 |
| Q1MS98_a4 | GSSGSHFVF | 0.8093 | 1.0857 |  | |  | No | Y | Non-Toxin | 0.5 | 924.1 |
| Q1MS98_a5 | KNSGGVNSF | 1.4182 | 1.4856 |  | |  | No | Y | Non-Toxin | 1 | 909.09 |
| Q1MS98_a6 | RIALAVLTL | 1.6294 | 0.8752 |  | |  | No |  | Non-Toxin | 1 | 969.37 |
| Q1MS98_a7 | QVKSEGARM | 1.9196 | 1.6648 |  | | Several | No |  | Non-Toxin | 1 | 1005.28 |
| Q1MS98_a8 | LWETGKPSF | 2.8964 | -0.4501 |  | |  | No | Y | Non-Toxin | 0 | 1064.33 |
| Q1MS98_a9 | SSGSHFVFH | 4.5246 | 0.6368 |  | |  | No | Y | Non-Toxin | 1 | 1004.19 |
| Q1MS98_a10 | FVFHDSPQK | 4.8405 | 0.5972 |  | |  | No | Y | Non-Toxin | 0.5 | 1104.35 |
| Q1MS98_sla2_a1 | EGARMGKLW | 0.6178 | 0.5006 |  | |  | No | Y | Non-Toxin | 1 | 1047.37 |
| Q1MS98_sla2_a2 | KNSGGVNSF | 0.6652 | 1.4856 |  | |  | No | Y | Non-Toxin | 1 | 909.09 |
| Q1MS98_sla2_a3 | GVRKGIEAY | 1.1652 | 0.6905 |  | |  | No |  | Non-Toxin | 1 | 992.27 |
| Q1MS98_sla2_a4 | GSSGSHFVF | 1.8782 | 1.0857 |  | | 2.00 | No | Y | Non-Toxin | 0.5 | 924.1 |
| Q1MS98_sla2_a5 | MNCKKGLKL | 3.1459 | 1.5054 |  | |  | No |  | Non-Toxin | 3 | 1034.48 |
| Q1MPX4_a1 | SITPLKNSY | 0.0959 | -0.0427 |  | | 3.00 | Y |  | Non-Toxin | 1 | 1022.29 |
| Q1MPX4_a2 | YLSSNPISW | 0.1445 | 0.1889 |  | | 3.00 |  | Y | Non-Toxin | 0 | 1066.3 |
| Q1MPX4_a3 | QVESMMAWY | 0.2552 | 0.4226 |  | |  | No | Y | Non-Toxin | -1 | 1144.45 |
| Q1MPX4_a4 | ISFSTIISY | 0.4593 | 0.2703 |  | |  | No | Y | Non-Toxin | 0 | 1030.31 |
| Q1MPX4_a5 | RTQKNNNYL | 0.7282 | 0.8067 | Yes | | Several | No |  | Non-Toxin | 2 | 1150.39 |
| Q1MPX4_a6 | FSGGQTSFF | 0.869 | -0.246 |  | |  | No |  | Non-Toxin | 0 | 977.16 |
| Q1MPX4_a7 | VQVESMMAW | 0.8802 | 0.6021 |  | |  | No |  | Non-Toxin | -1 | 1080.41 |
| Q1MPX4_a8 | AVSATMDGM | 0.9703 | 0.6668 |  | | Several | No |  | Non-Toxin | -1 | 882.13 |
| Q1MPX4_a9 | KQFEKIQAL | 1.0346 | -0.5342 |  | |  | No |  | Non-Toxin | 1 | 1104.45 |
| Q1MPX4_a10 | YRDQLQNEF | 1.3542 | 0.482 |  | | Several | No |  | Non-Toxin | -1 | 1212.41 |
| Q1MPX4_a11 | KNSYVSTNY | 1.6386 | 0.8103 |  | |  | No |  | Non-Toxin | 1 | 1075.26 |
| Q1MPX4_sla2_a1 | ISFSTIISY | 0.0293 | 0.2703 |  | | 6\3 | No | Y | Non-Toxin | 0 | 1030.31 |
| Q1MPX4_sla2_a2 | VQVESMMAW | 0.1451 | 0.6021 | X | | No | No |  | Non-Toxin | -1 | 1080.41 |
| Q1MPX4_sla2_a3 | SITPLKNSY | 0.1762 | -0.0427 |  | | 44715.00 | No |  | Non-Toxin | 1 | 1022.29 |
| Q1MPX4_sla2_a4 | KQFEKIQAL | 0.2011 | -0.5342 |  | |  | No |  | Non-Toxin | 1 | 1104.45 |
| Q1MPX4_sla2_a5 | KNSYVSTNY | 0.2361 | 0.8103 |  | | Several | No |  | Non-Toxin | 1 | 1075.26 |
| Q1MP78-a1 | TTSSHHGPY | 0.6263 | 0.5277 |  | | No | No | Y | Non-Toxin | 1 | 986.13 |
| Q1MP78-a2 | SSHHGPYAW | 0.6314 | -0.2024 |  | |  | No | Y | Non-Toxin | 1 | 1041.21 |
| Q1MP78-a3 | RQSSTVGLM | 2.0256 | 0.6957 |  | |  | No | Y | Non-Toxin | 1 | 978.26 |
| Q1MP78-a4 | HTARHTTKV | 2.5906 | 0.6285 |  | |  | No | Y | Non-Toxin | 3 | 1050.31 |
| Q1MP78-a5 | LSPDWSLQL | 2.8856 | 2.4267 | Yes | | 44656.00 | No | Y | Non-Toxin | -1 | 1058.33 |
| Q1MP78-a6 | RVNAFPGLV | 3.6866 | 0.1751 |  | |  | No |  | Non-Toxin | 1 | 972.28 |
| Q1MP78-a7 | HHGPYAWGY | 4.3923 | -0.2342 |  | |  | No | Y | Non-Toxin | 1 | 1087.29 |
| Q1MP78-a8 | SMKSESLVI | 5.2576 | 0.7159 |  | |  | No | Y | Non-Toxin | 0 | 993.31 |
| Q1MP78-a9 | STVGLMIGV | 5.7716 | 0.5637 |  | |  | No | Y | Non-Toxin | 0 | 876.22 |
| Q1MP78-a10 | DQHARVNAF | 5.9752 | 0.9103 |  | |  | No |  | Non-Toxin | 0.5 | 1057.25 |
| Q1MP78-a11 | QSSTVGLMI | 6.785 | 0.8916 |  | |  | No | Y | Non-Toxin | 0 | 935.24 |
| Q1MP78-_sla2a1 | SSHHGPYAW | 0.1016 | -0.2024 |  | | 44744.00 | No | Y | Non-Toxin | 1 | 1041.21 |
| Q1MP78-_sla2a2 | HHGPYAWGY | 0.1348 | -0.2342 |  | | Several | No | Y | Non-Toxin | 1 | 1087.29 |
| Q1MP78-_sla2a3 | DQHARVNAF | 0.2383 | 0.9103 |  | | Several | No |  | Non-Toxin | 0.5 | 1057.25 |
| Q1MP78-_sla2a4 | QKMHLSPDW | 0.5702 | 1.1578 |  | | Several | No | Y | Non-Toxin | 0.5 | 1141.44 |
| Q1MP78-_sla2a5 | TKGSKTARW | 0.6187 | 1.7457 | X | | several segments | No | Y | Non-Toxin | 3 | 1034.3 |
| Q1MP78-_sla2a6 | ARHTTKVGF | 0.6727 | 0.4548 |  | | several segments | No | Y | Non-Toxin | 2.5 | 1016.29 |
| Q1MRZ2_a1 | SIDTIPLQF | 0.0029 | 1.5846 | Yes | | 7-2 | No |  | Non-Toxin | -1 | 1033.32 |
| Q1MRZ2_a2 | TLDFESETM | 0.2517 | 0.9238 |  | | Several | No |  | Non-Toxin | -3 | 1072.27 |
| Q1MRZ2_a3 | PVSQKQLEY | 0.6122 | 0.5163 |  | | Several | No | Y | Non-Toxin | 0 | 1091.36 |
| Q1MRZ2_a4 | SSDNGNTAI | 0.6877 | 1.0956 |  | |  |  | Y | Non-Toxin | -1 | 877.98 |
| Q1MRZ2_a5 | AIFKSTVTL | 0.6896 | -0.5069 |  | |  | No |  | Non-Toxin | 1 | 979.31 |
| Q1MRZ2_a6 | KQLEYIHSL | 1.1733 | -0.185 |  | |  | No | Y | Non-Toxin | 0.5 | 1130.45 |
| Q1MRZ2_a7 | SLSDNAEQI | 1.8687 | 0.1856 |  | | 7—2ch | No | Y | Non-Toxin | -2 | 976.13 |
| Q1MRZ2_a8 | ACNVGTLDF | 2.4939 | 1.1445 |  | |  | No | Y | Non-Toxin | -1 | 939.17 |
| Q1MRZ2_a9 | ASSTVQTAH | 2.8709 | 1.0451 |  | |  | No | Y | Non-Toxin | 0.5 | 901.06 |
| Q1MRZ2_a10 | AMEKFSKDL | 3.0812 | -0.2848 |  | |  | No |  | Non-Toxin | 0 | 1068.37 |
| Q1MRZ2_a11 | SDNGNTAIF | 4.0194 | 0.4229 |  | |  | No |  | Non-Toxin | -1 | 938.08 |
| Q1MRZ2_sla2_a1 | KQLEYIHSL | 0.3552 | -0.185 |  | |  | No |  | Non-Toxin | 0.5 | 1130.45 |
| Q1MRZ2_sla2_a2 | SIDTIPLQF | 0.7842 | 1.5846 |  | | Several | No |  | Non-Toxin | -1 | 1033.32 |
| Q1MRZ2_sla2_a3 | AEQIAMEKF | 1.1438 | 0.5127 |  | | 8—1 | No | Y | Non-Toxin | -1 | 1066.36 |
| Q1MRZ2_sla2_a4 | SDNGNTAIF | 1.8525 | 0.4229 |  | |  | No |  | Non-Toxin | -1 | 938.08 |
| Q1MRZ2_sla2_a5 | SQKQLEYIH | 2.6048 | 0.2273 |  | |  | No |  | Non-Toxin | 0.5 | 1145.42 |
| Q1MNT3_a1 | KIDVSPNEF | 0.0072 | 1.1371 | Yes | | all 7—2 | No |  | Non-Toxin | -1 | 1048.28 |
| Q1MNT3_a2 | RVTQIDNRY | 0.1283 | 0.8855 |  | | Several | No | Y | Non-Toxin | 1 | 1164.41 |
| Q1MNT3_a3 | EIYRPFTLY | 0.2036 | -0.1112 |  | |  | No | Y | Non-Toxin | 0 | 1201.51 |
| Q1MNT3_a4 | SLEGHISSL | 0.2257 | 1.0793 |  | |  | No | Y | Non-Toxin | -0.5 | 942.17 |
| Q1MNT3_a5 | HVKPLIAAY | 0.3211 | 0.3646 |  | |  | No |  | Non-Toxin | 1.5 | 1011.36 |
| Q1MNT3_a6 | SAFQELMPY | 0.4018 | 0.2711 |  | |  | No |  | Non-Toxin | -1 | 1085.36 |
| Q1MNT3_a7 | ASIRSPEIY | 0.4661 | 1.0315 |  | |  | No |  | Non-Toxin | 0 | 1035.28 |
| Q1MNT3_a8 | SVGLAYNRY | 0.5236 | 0.4960 |  | |  | No |  | Non-Toxin | 1 | 1042.28 |
| Q1MNT3_a9 | TSEAGSHSL | 0.5275 | 1.3605 |  | |  | No | Y | Non-Toxin | -0.5 | 888.02 |
| Q1MNT3_a10 | FTDTKDTNL | 0.5442 | 1.4584 |  | | Several | No | Y | Non-Toxin | -1 | 1054.24 |
| Q1MNT3_a11 | LIAAYDSKY | 0.6042 | 0.4105 |  | |  | No | Y | Non-Toxin | 0 | 1043.3 |
| Q1MNT3_sla2_a1 | HVKPLIAAY | 0.0089 | 0.3646 |  | |  | No | Y | Non-Toxin | 1.5 | 1011.36 |
| Q1MNT3_sla2_a2 | HQVNVHFQY | 0.0089 | 1.5146 | X | | cymo 7 — 2 | No |  | Non-Toxin | 1 | 1171.42 |
| Q1MNT3_sla2_a3 | TERSCIQSW | 0.046 | 0.3457 |  | | cym6/3 | No | Y | Non-Toxin | 0 | 1109.34 |
| Q1MNT3_sla2_a4 | TKAELGMSY | 0.0526 | 1.0472 | X | | chymo 7/2 | No | Y | Non-Toxin | 0 | 999.27 |
| Q1MNT3_sla2_a5 | EIYRPFTLY | 0.079 | -0.1112 |  | | cym5/4 | No | Y | Non-Toxin | 0 | 1201.51 |
| Q1MPE6_a1 | SVNESLIGF | 0.0417 | 0.4103 | Yes | | No | NO- |  | Non-Toxin | -1 | 965.2 |
| Q1MPE6_a2 | GISSSSISF | 0.206 | 1.0459 |  | |  |  |  | Non-Toxin | 0 | 884.07 |
| Q1MPE6_a3 | KAVASLYSY | 0.4555 | 0.1885 |  | | Several | No |  | Non-Toxin | 1 | 1001.26 |
| Q1MPE6_a4 | QVASQANQM | 0.5594 | 0.6383 |  | | No | No | Y | Non-Toxin | 0 | 976.2 |
| Q1MPE6_a5 | DTDATGLRL | 0.7455 | 2.5289 |  | | Several | No |  | Non-Toxin | -1 | 961.16 |
| Q1MPE6_a6 | SQKAVASLY | 0.7536 | 0.4233 |  | |  | No |  | Non-Toxin | 1 | 966.22 |
| Q1MPE6_a7 | ETLPSSLSL | 0.8494 | 0.3893 |  | |  | No | Y | Non-Toxin | -1 | 946.19 |
| Q1MPE6_a8 | AQETLPSSL | 1.1348 | 0.2406 |  | |  | No | Y | Non-Toxin | -1 | 945.16 |
| Q1MPE6_a9 | TLPSSLSLM | 1.1689 | 0.1735 |  | |  | No |  | Non-Toxin | 0 | 948.27 |
| Q1MPE6_a10 | KSINKIGNF | 1.2014 | -1.0421 |  | |  | No |  | Non-Toxin | 2 | 1020.33 |
| Q1MPE6_a11 | SLYSYNDKL | 2.0026 | 0.3304 |  | |  | No |  | Non-Toxin | 0 | 1102.33 |
| Q1MPE6_sla2_a1 | SQKAVASLY | 0.0369 | 0.4233 |  | | Several | No |  | Non-Toxin | 1 | 966.22 |
| Q1MPE6_sla2_a2 | KAVASLYSY | 0.3388 | 0.1885 |  | |  | No |  | Non-Toxin | 1 | 1001.26 |
| Q1MPE6_sla2_a3 | SVNESLIGF | 0.552 | 0.4103 |  | | No | No | Y | Non-Toxin | -1 | 965.2 |
| Q1MPE6_sla2_a4 | TNTPIQNNL | 0.7229 | 0.1083 |  | |  | No | Y | Non-Toxin | 0 | 1014.24 |
| Q1MPE6_sla2_a5 | EQKKEVEEL | 1.2016 | 0.7596 |  | |  | No |  | Non-Toxin | -2 | 1131.38 |
| Q1MP58_a1 | FLASGTAAY | 0.0906 | -0.1122 |  | |  | check 1-20 | | Non-Toxin | 0 | 900.11 |
| Q1MP58_a2 | KTEGKDEAF | 0.1847 | 1.0482 | Yes | | Several | check | Y | Non-Toxin | -1 | 1024.21 |
| Q1MP58_a3 | MSNTKISTY | 0.2743 | 0.555 |  | | Several | check | Y | Non-Toxin | 1 | 1044.31 |
| Q1MP58_a4 | GLDKQITPM | 0.299 | 0.4946 |  | | Several | check |  | Non-Toxin | 0 | 1002.33 |
| Q1MP58_a5 | KHDDYPNNY | 0.5384 | 0.0622 |  | |  | check | Y | Non-Toxin | -0.5 | 1165.3 |
| Q1MP58_a6 | QINSPTIDL | 0.9951 | 1.526 |  | | asp7-2 | check | Y | Non-Toxin | -1 | 1000.25 |
| Q1MP58_a7 | AQDLLKRRL | 1.3517 | 0.3389 |  | |  | check | Y | Non-Toxin | 2 | 1112.47 |
| Q1MP58_a8 | VVEGKSVNI | 1.3863 | 1.8617 |  | | 5—4 | check |  | Non-Toxin | 0 | 944.23 |
| Q1MP58_a9 | KQITPMMLL | 1.7695 | 0.8407 |  | | 1—8 | check | Y | Non-Toxin | 1 | 1074.55 |
| Q1MP58_a10 | NTKISTYSF | 1.8447 | 1.2298 |  | | Several | check |  | Non-Toxin | 1 | 1060.29 |
| Q1MP58_a11 | QIANAQDLL | 2.1283 | -0.3084 |  | |  | check |  | Non-Toxin | -1 | 985.24 |
| Q1MP58_a12 | FGDKPGSTI | 2.1492 | 1.2373 |  | | Several | check |  | Non-Toxin | 0 | 921.14 |
| Q1MP58_sla2_a1 | MSNTKISTY | 0.0699 | 0.555 |  | | Several | check | Y | Non-Toxin | 1 | 1044.31 |
| Q1MP58_sla2_a2 | NTKISTYSF | 0.264 | 1.2298 | X | | Several | check | Y | Non-Toxin | 1 | 1060.29 |
| Q1MP58_sla2_a3 | VRITLAQGF | 0.376 | 0.4452 |  | |  | Y |  | Non-Toxin | 1 | 1004.33 |
| Q1MP58_sla2_a4 | KHDDYPNNY | 0.6387 | 0.0622 |  | |  | check | Y | Non-Toxin | -0.5 | 1165.3 |
| Q1MP58_sla2_a5 | KEGMLLVNF | 1.2761 | 0.0741 |  | |  | check |  | Non-Toxin | 0 | 1050.42 |
| Q1MSA8_a1 | NLQGGYKTY | 0.4124 | 0.7673 |  | |  | No | Y | Non-Toxin | 1 | 1043.28 |
| Q1MSA8_a2 | QVDTSTNNI | 0.4173 | 1.3258 |  | |  | No | Y | Non-Toxin | -1 | 991.15 |
| Q1MSA8_a3 | IISREATPF | 0.7846 | -0.8852 |  | |  | No |  | Non-Toxin | 0 | 1033.31 |
| Q1MSA8_a4 | STQQSSINI | 1.7304 | 1.4944 | Yes | | No | No |  | Non-Toxin | 0 | 977.17 |
| Q1MSA8_a5 | EVDFFEKMM | 1.7315 | -0.2551 |  | |  | check1-20 | | Non-Toxin | -2 | 1175.5 |
| Q1MSA8_a6 | FLSKEEVDF | 1.8696 | -0.4237 |  | |  | No |  | Non-Toxin | -2 | 1113.35 |
| Q1MSA8_a7 | KMMQKNINL | 2.0264 | 0.9067 |  | |  | No | Y | Non-Toxin | 2 | 1119.55 |
| Q1MSA8_a8 | LSKEEVDFF | 2.3388 | -0.5281 |  | |  | No | Y | Non-Toxin | -2 | 1113.35 |
| Q1MSA8_a9 | TIQQTTPNI | 2.4051 | 0.7842 |  | |  | No |  | Non-Toxin | 0 | 1015.27 |
| Q1MSA8_a10 | KIDNAPSVQ | 2.4736 | 1.0089 |  | |  | No |  | Non-Toxin | 0 | 971.2 |
| Q1MSA8_sla2_a1 | EEVDFFEKM | 0.2573 | -0.8203 |  | |  | No |  | Non-Toxin | -3 | 1173.42 |
| Q1MSA8_sla2_a2 | DNAPSVQSM | 0.3223 | 0.4717 |  | |  | No |  | Non-Toxin | -1 | 948.13 |
| Q1MSA8_sla2_a3 | KNINLQGGY | 0.5342 | 1.3536 |  | | 8—1 | No | Y | Non-Toxin | 1 | 1006.27 |
| Q1MSA8_sla2_a4 | QKNTNSSTF | 1.2398 | 1.2475 |  | |  | No |  | Non-Toxin | 1 | 1026.19 |
| Q1MSA8_sla2_a5 | NLQGGYKTY | 2.0315 | 0.7673 |  | |  | No |  | Non-Toxin | 1 | 1043.28 |
| Q1MQK8_a1 | KQEPIYVNY | 0.0882 | 0.7440 | Yes | | Several |  |  | Non-Toxin | 0 | 1153.43 |
| Q1MQK8_a2 | AAIIHGVQY | 0.3556 | -0.3104 |  | |  | No | Y | Non-Toxin | 0.5 | 971.26 |
| Q1MQK8_a3 | QSSNSTYVY | 0.5464 | 1.1286 |  | |  | No | Y | Non-Toxin | 0 | 1048.19 |
| Q1MQK8_a4 | QVQSEGSSL | 0.7334 | 1.4263 |  | |  | No |  | Non-Toxin | -1 | 934.1 |
| Q1MQK8_a5 | TTGDRGVRF | 0.7796 | 1.928 |  | | Several | No |  | Non-Toxin | 1 | 1008.22 |
| Q1MQK8_a6 | MVTSIQSSL | 1.3976 | 0.0882 |  | | No | No | Y | Non-Toxin | 0 | 965.26 |
| Q1MQK8_a7 | ILKKTKSSF | 1.8409 | -0.8149 |  | | No | No |  | Non-Toxin | 3 | 1051.42 |
| Q1MQK8_a8 | AIIHGVQYV | 1.9124 | -0.811 |  | |  | No |  | Non-Toxin | 0.5 | 999.32 |
| Q1MQK8_a9 | YLDVSGTQQ | 1.9203 | 1.3762 |  | | Several | No | Y | Non-Toxin | -1 | 1010.2 |
| Q1MQK8_a10 | FNNGPRVTF | 1.9508 | 0.3366 |  | |  | No | Y | Non-Toxin | 1 | 1051.29 |
| Q1MQK8_a11 | SVQDNSKAH | 1.9631 | 1.1924 |  | |  | No | Y | Non-Toxin | 0.5 | 985.14 |
| Q1MQK8_sl2_a1 | ENSNSGYSY | 0.0869 | 1.282 | X | | cymo 7/2 | No | Y | Non-Toxin | -1 | 1020.09 |
| Q1MQK8_sl2_a2 | KQEPIYVNY | 0.1252 | 0.7440 |  | | Several | No |  | Non-Toxin | 0 | 1153.43 |
| Q1MQK8_sl2_a3 | FNNGPRVTF | 0.1613 | 0.3366 |  | | Several | No |  | Non-Toxin | 1 | 1051.29 |
| Q1MQK8_sl2_a4 | AAIIHGVQY | 0.1825 | -0.3104 |  | | No | No |  | Non-Toxin | 0.5 | 971.26 |
| Q1MQK8_sl2_a5 | QSSNSTYVY | 0.2468 | 1.1286 |  | |  | No |  | Non-Toxin | 0 | 1048.19 |
| QMQK7_a1 | PVDSPDTIY | 0.1036 | 0.0639 |  | | Several | No |  | Non-Toxin | -2 | 1006.19 |
| QMQK7_a2 | SSEPIYAEL | 0.3912 | 0.4236 |  | |  | No | Y | Non-Toxin | -2 | 1008.21 |
| QMQK7_a3 | RVKHSTMKM | 0.9779 | 1.1633 | Yes | | several segments | No | Y | Non-Toxin | 3.5 | 1117.52 |
| QMQK7_a4 | RTHQALKDY | 1.0879 | 0.4888 |  | | Several | No |  | Non-Toxin | 1.5 | 1131.38 |
| QMQK7_a5 | TVVHPSTPL | 1.3515 | -0.3114 |  | | No | No |  | Non-Toxin | 0.5 | 950.23 |
| QMQK7_a6 | VTEPIYAEI | 1.4225 | 0.2754 |  | | Several | No |  | Non-Toxin | -2 | 1034.3 |
| QMQK7_a7 | ILSKLRASW | 1.4986 | -0.1235 |  | | Several | No |  | Non-Toxin | 2 | 1073.43 |
| QMQK7_a8 | YAEIKTTSI | 2.4537 | 0.662 |  | | Several | No |  | Non-Toxin | 0 | 1025.29 |
| QMQK7_a9 | KSVSPGPHV | 2.6932 | 0.5703 |  | | Several | No | y | Non-Toxin | 1.5 | 907.16 |
| QMQK7_a10 | STPRIVPRH | 3.2531 | 0.449 |  | | Several | No |  | Non-Toxin | 2.5 | 1062.36 |
| QMQK7_a11 | IQFKHSSSH | 3.8681 | 1.2459 |  | |  | No | Y | Non-Toxin | 2 | 1070.3 |
| QMQK7_sla2_a1 | KESSILSKL | 0.5947 | 0.2039 |  | |  | No | Y | Non-Toxin | 1 | 1004.32 |
| QMQK7_sla2_a2 | ILSKLRASW | 0.6559 | -0.1235 |  | |  | No |  | Non-Toxin | 2 | 1073.43 |
| QMQK7_sla2_a3 | NNPSSEPIY | 1.7176 | 0.1972 |  | |  | No |  | Non-Toxin | -1 | 1020.18 |
| QMQK7_sla2_a4 | RTHQALKDY | 1.8628 | 0.4888 |  | | Several | No |  | Non-Toxin | 1.5 | 1131.38 |
| QMQK7_sla2_a5 | IQFKHSSSH | 2.3375 | 1.2459 |  | |  | No |  | Non-Toxin | 2 | 1070.3 |
| Q1MP04_a1 | IMVEAIEEY | 0.2015 | 0.0700 |  | | No | No | Y | Non-Toxin | -3 | 1096.39 |
| Q1MP04_a2 | YVEPPKCHL | 0.3704 | -0.3849 |  | |  | No |  | Non-Toxin | 0.5 | 1085.41 |
| Q1MP04_a3 | KTLSNTTSV | 1.3852 | 0.6007 |  | |  | No |  | Non-Toxin | 1 | 950.18 |
| Q1MP04_a4 | LSNTTSVKL | 2.1433 | 1.245 |  | | 1---8 | No | Y | Non-Toxin | 1 | 962.24 |
| Q1MP04_a5 | MVEAIEEYI | 3.4122 | -0.3577 |  | |  | No |  | Non-Toxin | -3 | 1096.39 |
| Q1MP04_a6 | YNDYLNNGL | 3.9994 | -0.2062 |  | |  | No |  | Non-Toxin | -1 | 1085.27 |
| Q1MP04_a7 | FRQEGINVY | 4.152 | 0.4863 |  | |  | No |  | Non-Toxin | 0 | 1125.38 |
| Q1MP04_a8 | KLEDSVKTR | 4.5116 | 1.8415 | Yes | | Several | No |  | Non-Toxin | 1 | 1075.35 |
| Q1MP04_a9 | LNELANGNY | 4.55 | 0.021 |  | |  | No |  | Non-Toxin | -1 | 1007.2 |
| Q1MP04_a10 | GINVYNDYL | 5.4983 | 0.6166 |  | |  | No |  | Non-Toxin | -1 | 1070.3 |
| Q1MP04_a11 | LPVEEAIAW | 6.877 | -0.3704 |  | |  | No |  | Non-Toxin | -2 | 1027.31 |
| Q1MP04_sla2_a1 | FRQEGINVY | 0.3479 | 0.4863 |  | | Several | No |  | Non-Toxin | 0 | 1125.38 |
| Q1MP04_sla2_a2 | EGINVYNDY | 0.4432 | 0.1607 |  | | Several | No |  | Non-Toxin | -2 | 1086.25 |
| Q1MP04_sla2_a3 | IMVEAIEEY | 0.7615 | 0.07 |  | | No | No | Y | Non-Toxin | -3 | 1096.39 |
| Q1MP04_sla2_a4 | DKAEKREAF | 0.7939 | 0.7170 |  | | Several | No |  | Non-Toxin | 0 | 1093.31 |
| Q1MP04_sla2_a5 | LPVEEAIAW | 2.0043 | -0.3704 |  | |  | No |  | Non-Toxin | -2 | 1027.31 |
| CAJ210_a1 | HYDASGLRF | 0.3867 | 1.6163 | Yes | | Several | No |  | Non-Toxin | 0.5 | 1065.27 |
| CAJ210_a2 | QLNVIEGHY | 0.6781 | 0.641 |  | | No | No | Y | Non-Toxin | -0.5 | 1072.33 |
| CAJ210_a3 | TTDTIEQAI | 0.7838 | 0.3584 |  | |  | No | Y | Non-Toxin | -2 | 991.19 |
| CAJ210_a4 | GSDATLAVL | 0.8403 | 0.9717 |  | | 7—2 | No | Y | Non-Toxin | -1 | 846.07 |
| CAJ210_a5 | IIRGSTPHF | 0.9134 | -0.0037 |  | |  | No | Y | Non-Toxin | 1.5 | 1027.32 |
| CAJ210_a6 | LQTDVPIGF | 1.321 | 0.4273 |  | |  | No | Y | Non-Toxin | -1 | 989.27 |
| CAJ210_a7 | RGSTPHFDY | 1.5391 | 0.8285 |  | | sev | No | Y | Non-Toxin | 0.5 | 1079.25 |
| CAJ210_a8 | ILASRFNDF | 1.9898 | 0.0703 |  | |  | No | Y | Non-Toxin | 0 | 1082.34 |
| CAJ210_a9 | RFAILASRF | 2.3732 | 0.7842 |  | | Several | No | Y | Non-Toxin | 2 | 1080.41 |
| CAJ210_a10 | AIIRGSTPH | 2.9539 | -0.1853 |  | |  | No | Y | Non-Toxin | 1.5 | 951.22 |
| CAJ210_a11 | AVLESIRVI | 3.5478 | -0.4188 |  | |  | No | Y | Non-Toxin | 0 | 999.35 |
| CAJ210_sla2_a1 | KKLSLTSKF | 0.544 | 0.3235 |  | |  | No | Y | Non-Toxin | 3 | 1051.42 |
| CAJ210_sla2_a2 | LQTDVPIGF | 0.5809 | 0.4273 |  | | Several | No | Y | Non-Toxin | -1 | 989.27 |
| CAJ210_sla2_a3 | ESIRVIQKI | 0.7527 | -0.6259 |  | |  | No | Y | Non-Toxin | 1 | 1085.45 |
| CAJ210_sla2_a4 | SKFDGIIIL | 0.8582 | -0.3638 |  | |  | No | Y | Non-Toxin | 0 | 1005.36 |
| CAJ210_sla2_a5 | QLNVIEGHY | 2.1999 | 0.6410 |  | | No | No | Y | Non-Toxin | -0.5 | 1072.33 |
| CAJ55195_a1 | PIDNIHHMY | 0.0585 | -0.5207 |  | | 7/2asn | No | Y | Non-Toxin | 0 | 1139.43 |
| CAJ55195_a2 | SLLGVAGEY | 0.2437 | -0.0225 |  | |  | No | Y | Non-Toxin | -1 | 908.15 |
| CAJ55195_a3 | RVIHEDIQW | 0.3597 | 0.3754 |  | | Several | No | Y | Non-Toxin | -0.5 | 1195.48 |
| CAJ55195_a4 | VTDKYIQSI | 0.4499 | 0.8085 | Yes | | Several | No |  | Non-Toxin | 0 | 1066.35 |
| CAJ55195_a5 | SLLNITKNF | 0.7208 | -1.1489 |  | | 6—3 | No | Y | Non-Toxin | 1 | 1049.37 |
| CAJ55195_a6 | YIIGLFKLY | 0.8322 | 0.0357 |  | | Several | No |  | Non-Toxin | 1 | 1129.55 |
| CAJ55195_a7 | KVVSGVIKL | 0.9061 | 0.1602 |  | | Several | No |  | Non-Toxin | 2 | 942.35 |
| CAJ55195_a8 | SMFTLICNY | 1.3952 | 0.8114 |  | | Several | No | Y | Non-Toxin | 0 | 1091.43 |
| CAJ55195_a9 | GFQLGSQLY | 1.4827 | 0.7278 |  | | Several | No | Y | Non-Toxin | 0 | 1012.27 |
| CAJ55195_a10 | ITDVFQRKV | 2.2206 | 0.4148 |  | | Several | No | Y | Non-Toxin | 1 | 1105.43 |
| CAJ55195_a11 | EVVRKVPIF | 2.4221 | 0.1135 |  | | Several | No | Y | Non-Toxin | 1 | 1086.47 |
| CAJ55195_sla2_a1 | YIIGLFKLY | 0.4028 | 0.0357 |  | | Several | No |  | Non-Toxin | 1 | 1129.55 |
| CAJ55195_sla2_a2 | RVIHEDIQW | 0.7048 | .3754 |  | | Several | No |  | Non-Toxin | -0.5 | 1195.48 |
| CAJ55195_sla2_a3 | SMFTLICNY | 0.7059 | 0.8114 |  | | Several | No |  | Non-Toxin | 0 | 1091.43 |
| CAJ55195_sla2_a4 | IERIITDVF | 0.8392 | -0.4405 |  | | Several | No |  | Non-Toxin | -1 | 1105.43 |
| CAJ55195_sla2_a5 | EVVRKVPIF | 0.8608 | 0.1135 |  | | Several | No |  | Non-Toxin | 1 | 1086.47 |
| CAJ45511_a1 | KVVESIVKY | 0.0305 | -0.3012 |  | | Several | No |  | Non-Toxin | 1 | 1064.42 |
| CAJ45511_a2 | SSDKDKEYF | 0.0638 | .3302 |  | |  | No |  | Non-Toxin | -1 | 1118.27 |
| CAJ45511_a3 | YVSPDAFVY | 0.0856 | 0.7211 | Yes | | Several | No |  | Non-Toxin | -1 | 1060.28 |
| CAJ45511_a4 | SIVKYQQTF | 0.2473 | -0.4067 |  | | Several | No |  | Non-Toxin | 1 | 1113.41 |
| CAJ45511_a5 | ITMEELKEY | 0.3342 | 0.2086 |  | | 7—2 | No | Y | Non-Toxin | -2 | 1155.46 |
| CAJ45511_a6 | FVYQVDNEF | 0.376 | 0.6099 |  | | Several | No |  | Non-Toxin | -2 | 1160.37 |
| CAJ45511_a7 | QVDNEFIIL | 0.4283 | 0.5921 |  | |  | No |  | Non-Toxin | -2 | 1090.38 |
| CAJ45511_a8 | EMSSLEARY | 0.6378 | 1.4253 |  | | 8—1 | No | Y | Non-Toxin | -1 | 1085.31 |
| CAJ45511_a9 | YIATPFGIF | 0.6844 | -0.0431 |  | |  | No | Y | Non-Toxin | 0 | 1028.34 |
| CAJ45511_a10 | LIQIEVLGY | 0.9522 | 0.6002 |  | |  | No | Y | Non-Toxin | -1 | 1047.41 |
| CAJ45511_a11 | SMDIPSSSR | 0.9841 | 0.302 |  | |  | No | Y | Non-Toxin | 0 | 979.18 |
| CAJ45511_sla2_a1 | KVVESIVKY | 0.0968 | -0.3012 |  | |  | No |  | Non-Toxin | 1 | 1064.42 |
| CAJ45511_sla2_a2 | SEKIRSAAW | 0.102 | 0.5349 |  | |  | No |  | Non-Toxin | 1 | 1047.29 |
| CAJ45511_sla2_a3 | TEHDPSEAY | 0.1095 | 0.6936 | X | | Asn6/3 | No |  | Non-Toxin | -2.5 | 1048.14 |
| CAJ45511_sla2_a4 | ITMEELKEY | 0.2715 | 0.2086 |  | |  | No |  | Non-Toxin | -2 | 1155.46 |
| CAJ45511_sla2_a5 | TNKYIATPF | 0.3195 | 0.2338 |  | | Several | No |  | Non-Toxin | 1 | 1054.33 |
| CAJ54315_a1 | HPFEEVTHF | 2.4748 | -1.1105 |  | |  | No |  | Non-Toxin | -1 | 1142.36 |
| CAJ54315_a2 | VTHFESISL | 2.5785 | 0.2776 |  | |  | No |  | Non-Toxin | -0.5 | 1032.29 |
| CAJ54315_a3 | ISDSDCKSH | 2.7769 | 1.2429 | Yes | | Several | No |  | Non-Toxin | -0.5 | 991.15 |
| CAJ54315_a4 | SSQNTQRIL | 3.692 | 0.4399 |  | |  | No |  | Non-Toxin | 1 | 1046.28 |
| CAJ54315_a5 | KSHFSPNSI | 4.4559 | 0.2015 |  | |  | No |  | Non-Toxin | 1.5 | 1016.24 |
| CAJ54315_a6 | SDSDCKSHF | 6.2809 | 0.7207 |  | | Several | No |  | Non-Toxin | -0.5 | 1025.16 |
| CAJ54315_a7 | CSSQNTQRI | 7.5582 | 1.0920 |  | |  | No |  | Non-Toxin | 1 | 1036.25 |
| CAJ54315_a8 | ICIQCGHPF | 7.7231 | 0.7778 |  | | No | No | Y | Non-Toxin | 0.5 | 1017.36 |
| CAJ54315_a9 | AANIKRVTI | 10.0514 | 0.3499 |  | |  | No |  | Non-Toxin | 2 | 985.32 |
| CAJ54315_a10 | RILSAANIK | 10.53 | 0.6315 |  | |  | No |  | Non-Toxin | 2 | 985.32 |
| CAJ54315_a11 | ILSAANIKR | 11.3806 | 0.2112 |  | |  | No |  | Non-Toxin | 2 | 985.32 |
| CAJ54315_sla2_a1 | HPFEEVTHF | 0.1448 | -1.1105 | X | | cymo6//3 | No |  | Non-Toxin | -1 | 1142.36 |
| CAJ54315_sla2_a2 | EEVTHFESI | 1.2259 | -0.2731 |  | |  | No |  | Non-Toxin | -2.5 | 1090.28 |
| CAJ54315_sla2_a3 | SDSDCKSHF | 2.9005 | 0.7207 |  | | Several | No |  | Non-Toxin | -0.5 | 1025.16 |
| CAJ54315_sla2_a4 | QRILSAANI | 5.812 | -0.1807 |  | |  | No |  | Non-Toxin | 1 | 985.28 |
| CAJ54302_a1 | NVETGREFY | 0.1418 | 0.7103 | Yes | | Several | No |  | Non-Toxin | -1 | 1114.3 |
| CAJ54302_a2 | NIVPDSLQF | 0.2173 | 0.4796 |  | | Several | No |  | Non-Toxin | -1 | 1032.29 |
| CAJ54302_a3 | KLQRVKVCY | 0.5144 | 0.3031 |  | |  | No |  | Non-Toxin | 3 | 1136.55 |
| CAJ54302_a4 | SLEGAILEI | 0.7529 | 1.1080 |  | | No | No | Y | Non-Toxin | -2 | 944.23 |
| CAJ54302_a5 | KIFTEGTSL | 0.767 | -0.2938 |  | |  | No | Y | Non-Toxin | 0 | 995.27 |
| CAJ54302_a6 | VPDSLQFAF | 1.0915 | 0.7737 |  | |  | No | Y | Non-Toxin | -1 | 1023.27 |
| CAJ54302_a7 | EIEKIPLML | 1.8788 | 0.6124 |  | |  | No | Y | Non-Toxin | -1 | 1085.51 |
| CAJ54302_a8 | SLQFAFKIF | 1.9067 | 2.1348 |  | |  | No | Y | Non-Toxin | 1 | 1100.45 |
| CAJ54302_a9 | ILSIIQEEM | 2.0409 | -0.4515 |  | |  | No | Y | Non-Toxin | -2 | 1075.43 |
| CAJ54302_a10 | LTNIVPDSL | 2.5927 | -0.1454 |  | |  | No | Y | Non-Toxin | -1 | 971.25 |
| CAJ54302_a11 | SLVTGILSI | 2.6374 | -0.1803 |  | |  | No | Y | Non-Toxin | 0 | 902.24 |
| CAJ54302_a12 | YNVETGREF | 2.6945 | 0.6095 |  | |  | No | Y | Non-Toxin | -1 | 1114.3 |
| CAJ54302_sla2_a1 | YNVETGREF | 0.2735 | 0.6095 |  | Several | | No | Y | Non-Toxin | -1 | 1114.3 |
| CAJ54302_sla2_a2 | LEIEKIPLM | 0.3169 | 0.5400 |  | Several | | No |  | Non-Toxin | -1 | 1085.51 |
| CAJ54302_sla2_a3 | HEMSLVTGI | 0.6716 | -0.0409 |  | |  | No |  | Non-Toxin | -0.5 | 986.29 |
| CAJ54302_sla2_a4 | EDKQKIFFI | 1.4219 | -0.4249 |  | |  | No |  | Non-Toxin | 0 | 1167.5 |
| CAJ54302_sla2_a5 | NIVPDSLQF | 2.0213 | 0.4796 |  | |  | No |  | Non-Toxin | -1 | 1032.29 |
| CTLEs were estimated by NetMHCpan-4.1. Top 286 CTLEs were assessed for antigenicity, toxicities, charge, digestive sensitivities, and signal peptide estimation, from which 25 were finalized. This table show all high ranked organized CTLEs further asses after being selected from several thousand epitopes. The antigenicity was evaluated by VaxiJen with the 0.4 threshold. Digestion by most common enzymes such as trypsin and chymotrypsin were marked with Y but if the cleaved CTLE was still at least 7 AA in length, it was not deemed non-favourable. S-P column indicate whether the epitope is part of the signal peptide or not (N). Docking analyses was not performed for all epitopes that were totally cleaved by digestion enzymes. ToxinPred was used to predict whether highly expressed epitopes were toxic for organisms. | | | | | | | | | | | |

**S3 Table:** Calculated energy term through g_mmpbsa and their standard deviation

| **CTLE identifier** | **van der Waal energy** | **Electrostattic energy** | **Polar solvation energy** | **SASA energy** | **Binding energy** |
| --- | --- | --- | --- | --- | --- |
|  |  |  |  |  |  |
| **C2** | -258.640 +/- 1.120 kJ/mol | -54.637 +/- 2.204 kJ/mol | 280.939 +/- 2.917 kJ/mol | -32.049 +/- 0.111 kJ/mol | -64.360 +/- 1.455 kJ/mol |
| **C3** | -305.097 +/- 0.821 kJ/mol | -416.286 +/- 2.476 kJ/mol | 676.788 +/- 2.839 kJ/mol | -38.231 +/- 0.053 kJ/mol | -82.787 +/- 1.468 kJ/mol |
| **C8** | -222.418 +/- 1.267 kJ/mol | -299.444 +/- 3.766 kJ/mol | 459.338 +/- 5.206 kJ/mol | -32.140 +/- 0.177 kJ/mol | -94.802 +/- 2.309 kJ/mol |
| **C9** | -288.335 +/- 1.123 kJ/mol | -373.766 +/- 4.096 kJ/mol | 482.701 +/- 4.898 kJ/mol | -40.306 +/- 0.102 kJ/mol | -219.579 +/- 2.396 kJ/mol |
|  |  |  |  |  |  |
| **C11** | -292.229 +/- 1.558 kJ/mol | -250.534 +/- 4.752 kJ/mol | 490.101 +/- 6.251 kJ/mol | -39.580 +/- 0.152 kJ/mol | -92.606 +/- 2.108 kJ/mol |
| **C12** | -291.569 +/- 1.041 kJ/mol | -473.894 +/- 2.343 kJ/mol | 666.458 +/- 3.199 kJ/mol | -40.301 +/- 0.094 kJ/mol | -139.531 +/- 1.658 kJ/mol |
| **C13** | -150.050 +/- 1.471 kJ/mol | -304.565 +/- 6.178 kJ/mol | 435.991 +/- 6.784 kJ/mol | -27.037 +/- 0.190 kJ/mol | -45.812 +/- 3.067 kJ/mol |
| **C14** | -220.529 +/- 0.881 kJ/mol | -82.806 +/- 2.312 kJ/mol | 264.880 +/- 2.888 kJ/mol | -28.142 +/- 0.099 kJ/mol | -66.530 +/- 1.496 kJ/mol |
| **C15** | -208.452 +/- 1.183 kJ/mol | -318.890 +/- 3.040 kJ/mol | 492.874 +/- 4.178 kJ/mol | -28.842 +/- 0.132 kJ/mol | -63.267 +/- 1.898 kJ/mol |
| **D2** | -282.537 +/- 0.985 kJ/mol | -279.862 +/- 2.742 kJ/mol | 462.122 +/- 3.369 kJ/mol | -37.693 +/- 0.108 kJ/mol | -137.880 +/- 1.621 kJ/mol |
| **D4** | -250.621 +/- 0.853 kJ/mol | -537.849 +/- 1.379 kJ/mol | 718.603 +/- 1.603 kJ/mol | -34.403 +/- 0.070 kJ/mol | -104.283 +/- 1.156 kJ/mol |
| **D5** | -207.637 +/- 0.940 kJ/mol | -372.101 +/- 6.886 kJ/mol | 301.940 +/- 6.052 kJ/mol | -27.669 +/- 0.127 kJ/mol | -305.634 +/- 2.254 kJ/mol |
| **D6** | -269.008 +/- 1.549 kJ/mol | -237.315 +/- 2.474 kJ/mol | 356.655 +/- 3.143 kJ/mol | -35.623 +/- 0.168 kJ/mol | -185.182 +/- 1.782 kJ/mol |
| **D7** | -280.749 +/- 1.198 kJ/mol | -137.502 +/- 2.477 kJ/mol | 238.707 +/- 2.447 kJ/mol | -35.505 +/- 0.113 kJ/mol | -214.851 +/- 1.817 kJ/mol |
| These evalutions are from 250 ns – 300 ns with dt set to 100 ps. | | | | | |

**S4 Table:** Top three cluster data of CTLEs docking into SLA peptides binding grooves

| **Cluster Name** | **Cluster Density** | **Average RMSD** | **Max RMSD** | **# of Elements** | **CTL Seq** | **SLA** |
| --- | --- | --- | --- | --- | --- | --- |
| cluster_1.pdb | 37.9799 | 1.71143 | 5.83354 | 65 | ALEQQIHLM | SLA 1 |
| cluster_2.pdb | 31.1483 | 3.82043 | 9.18302 | 119 | ALEQQIHLM | SLA 1 |
| cluster_3.pdb | 30.2772 | 4.19458 | 28.6531 | 127 | ALEQQIHLM | SLA 1 |
| cluster_1.pdb | 53.8895 | 2.82059 | 7.31439 | 152 | YVQSTAAMF | SLA 1 |
| cluster_2.pdb | 41.9634 | 2.64516 | 31.8727 | 111 | YVQSTAAMF | SLA 1 |
| cluster_3.pdb | 29.4038 | 1.93853 | 3.39908 | 57 | YVQSTAAMF | SLA 1 |
| cluster_1.pdb | 69.4393 | 2.693 | 5.30779 | 187 | SSGSSGSHF | SLA 1 |
| cluster_2.pdb | 60.5852 | 3.33415 | 13.8591 | 202 | SSGSSGSHF | SLA 1 |
| cluster_3.pdb | 36.1775 | 2.12839 | 4.89553 | 77 | SSGSSGSHF | SLA 1 |
| cluster_1.pdb | 60.8891 | 2.21715 | 4.62526 | 135 | TTSSHHGPY | SLA 1 |
| cluster_2.pdb | 43.3755 | 1.42938 | 3.22503 | 62 | TTSSHHGPY | SLA 1 |
| cluster_3.pdb | 40.0819 | 2.74438 | 6.59369 | 110 | TTSSHHGPY | SLA 1 |
| cluster_1.pdb | 61.9226 | 3.16524 | 8.07913 | 196 | SIDTIPLQF | SLA 1 |
| cluster_2.pdb | 45.5492 | 2.78819 | 8.77319 | 127 | SIDTIPLQF | SLA 1 |
| cluster_3.pdb | 36.7037 | 3.16044 | 6.31498 | 116 | SIDTIPLQF | SLA 1 |
| cluster_1.pdb | 57.6175 | 4.21747 | 16.2398 | 243 | KIDVSPNEF | SLA 1 |
| cluster_2.pdb | 35.3692 | 4.80644 | 13.7011 | 170 | KIDVSPNEF | SLA 1 |
| cluster_3.pdb | 17.595 | 6.64961 | 17.5668 | 117 | KIDVSPNEF | SLA 1 |
| cluster_1.pdb | 59.0844 | 2.08177 | 4.97761 | 123 | TSEAGSHSL | SLA 1 |
| cluster_2.pdb | 38.0006 | 3.0789 | 8.32823 | 117 | TSEAGSHSL | SLA 1 |
| cluster_3.pdb | 37.8648 | 5.01786 | 12.7471 | 190 | TSEAGSHSL | SLA 1 |
| cluster_1.pdb | 66.443 | 1.88131 | 11.8778 | 125 | QVASQANQM | SLA 1 |
| cluster_2.pdb | 53.7677 | 2.5108 | 7.04884 | 135 | QVASQANQM | SLA 1 |
| cluster_3.pdb | 36.2849 | 6.14581 | 19.46 | 223 | QVASQANQM | SLA 1 |
| cluster_1.pdb | 36.6322 | 2.75714 | 11.196 | 101 | KQITPMMLL | SLA 1 |
| cluster_2.pdb | 31.5612 | 4.62593 | 11.4371 | 146 | KQITPMMLL | SLA 1 |
| cluster_3.pdb | 25.0373 | 4.47332 | 24.8783 | 112 | KQITPMMLL | SLA 1 |
| cluster_1.pdb | 57.4852 | 2.59197 | 7.20091 | 149 | QVDTSTNNI | SLA 1 |
| cluster_2.pdb | 40.6828 | 3.8837 | 11.255 | 158 | QVDTSTNNI | SLA 1 |
| cluster_3.pdb | 38.9167 | 3.41755 | 23.9563 | 133 | QVDTSTNNI | SLA 1 |
| cluster_1.pdb | 52.7851 | 1.9892 | 8.4235 | 105 | MVTSIQSSL | SLA 1 |
| cluster_2.pdb | 42.3718 | 2.59607 | 21.737 | 110 | MVTSIQSSL | SLA 1 |
| cluster_3.pdb | 26.4031 | 6.77951 | 18.5314 | 179 | MVTSIQSSL | SLA 1 |
| cluster_1.pdb | 78.9373 | 0.684087 | 3.20552 | 54 | TVVHPSTPL | SLA 1 |
| cluster_2.pdb | 50.4218 | 2.04277 | 13.0371 | 103 | TVVHPSTPL | SLA 1 |
| cluster_3.pdb | 44.8679 | 3.74432 | 10.3353 | 168 | TVVHPSTPL | SLA 1 |
| cluster_1.pdb | 52.7254 | 3.20529 | 19.3228 | 169 | LSNTTSVKL | SLA 1 |
| cluster_2.pdb | 47.5216 | 4.22966 | 11.1837 | 201 | LSNTTSVKL | SLA 1 |
| cluster_3.pdb | 29.5842 | 3.6844 | 13.5665 | 109 | LSNTTSVKL | SLA 1 |
| cluster_1.pdb | 47.8979 | 2.19216 | 17.2013 | 105 | HYDASGLRF | SLA 1 |
| cluster_2.pdb | 40.5707 | 2.07046 | 5.14955 | 84 | HYDASGLRF | SLA 1 |
| cluster_3.pdb | 27.4215 | 4.01145 | 25.0527 | 110 | HYDASGLRF | SLA 1 |
| cluster_1.pdb | 91.2806 | 1.99385 | 5.61223 | 182 | ICIQCGHPF | SLA 1 |
| cluster_2.pdb | 80.6309 | 1.61229 | 3.5875 | 130 | ICIQCGHPF | SLA 1 |
| cluster_3.pdb | 70.046 | 1.28487 | 4.91053 | 90 | ICIQCGHPF | SLA 1 |
| cluster_1.pdb | 69.0581 | 2.93956 | 15.0048 | 203 | SLEGAILEI | SLA 1 |
| cluster_2.pdb | 60.1334 | 3.84146 | 27.2517 | 231 | SLEGAILEI | SLA 1 |
| cluster_3.pdb | 28.8925 | 2.42278 | 14.7316 | 70 | SLEGAILEI | SLA 1 |
| cluster_1.pdb | 54.9485 | 2.23846 | 10.8933 | 123 | SVNESLIGF | SLA 1 |
| cluster_2.pdb | 54.0253 | 3.53538 | 8.42602 | 191 | SVNESLIGF | SLA 1 |
| cluster_3.pdb | 46.9951 | 2.42579 | 11.519 | 114 | SVNESLIGF | SLA 1 |
| cluster_1.pdb | 96.8292 | 1.17733 | 6.26831 | 114 | QLNVIEGHY | SLA 1 |
| cluster_2.pdb | 42.9209 | 5.40529 | 30.4337 | 232 | QLNVIEGHY | SLA 1 |
| cluster_3.pdb | 27.1538 | 1.69406 | 4.5452 | 46 | QLNVIEGHY | SLA 1 |
| cluster_1.pdb | 166.792 | 0.407694 | 2.8834 | 68 | QLAPTPLLY | SLA 2 |
| cluster_2.pdb | 43.3515 | 2.5374 | 10.7399 | 110 | QLAPTPLLY | SLA 2 |
| cluster_3.pdb | 34.406 | 2.0636 | 21.8692 | 71 | QLAPTPLLY | SLA 2 |
| cluster_1.pdb | 71.7447 | 1.40777 | 13.0984 | 101 | ENSNSGYSY | SLA 2 |
| cluster_2.pdb | 47.0987 | 5.28677 | 15.8037 | 249 | ENSNSGYSY | SLA 2 |
| cluster_3.pdb | 28.4376 | 5.13404 | 16.0806 | 146 | ENSNSGYSY | SLA 2 |
| cluster_1.pdb | 27.2331 | 2.68057 | 8.07069 | 73 | ITMEELKEY | SLA 2 |
| cluster_2.pdb | 25.4988 | 7.41213 | 32.6749 | 189 | ITMEELKEY | SLA 2 |
| cluster_3.pdb | 19.5932 | 11.1773 | 57.6165 | 219 | ITMEELKEY | SLA 2 |
| cluster_1.pdb | 69.482 | 1.55436 | 10.434 | 108 | ISFSTIISY | SLA 2 |
| cluster_2.pdb | 49.1984 | 3.78061 | 23.8435 | 186 | ISFSTIISY | SLA 2 |
| cluster_3.pdb | 32.721 | 2.44492 | 7.22019 | 80 | ISFSTIISY | SLA 2 |
| cluster_1.pdb | 52.5205 | 1.9421 | 9.9575 | 102 | KNINLQGGY | SLA 2 |
| cluster_2.pdb | 49.8496 | 2.327 | 6.43581 | 116 | KNINLQGGY | SLA 2 |
| cluster_3.pdb | 37.1563 | 2.90664 | 11.4785 | 108 | KNINLQGGY | SLA 2 |
| cluster_1.pdb | 55.553 | 4.2482 | 20.2506 | 236 | VRITLAQGF | SLA 2 |
| cluster_2.pdb | 43.4373 | 2.71656 | 16.6837 | 118 | VRITLAQGF | SLA 2 |
| cluster_3.pdb | 16.8125 | 6.18589 | 16.4847 | 104 | VRITLAQGF | SLA 2 |
| cluster_1.pdb | 56.208 | 2.81099 | 15.0775 | 158 | HQVNVHFQY | SLA 2 |
| cluster_2.pdb | 39.3168 | 3.30648 | 16.1878 | 130 | HQVNVHFQY | SLA 2 |
| cluster_3.pdb | 29.2305 | 5.43952 | 32.0218 | 159 | HQVNVHFQY | SLA 2 |
| CABS-dock provides 10 high ranked models. For each cluster, the density is defined as the number of elements divided by average cluster RMSD. The average RMSDs are calculated for pairwise Cα atoms of models that are grouped in a specific cluster. The number of elements indicates the number of the models grouped in each cluster. | | | | | | |

**S5 Table:** B-cell epitope prediction, probability of being a helix, loop, or sheet for **Q1MPW1** through BepiPred - 2.0.

| **Entry** | **Position** | **Resn** | **Exposed/Buried** | **Helix** | **Sheet** | **Coil** | **Epitope** |
| --- | --- | --- | --- | --- | --- | --- | --- |
| Sequence | 1 | M | E | 0.003 | 0.003 | **0.994** | 0.207222 |
| Sequence | 2 | G | E | 0.018 | 0.088 | **0.893** | 0.238444 |
| Sequence | 3 | K | E | 0.021 | 0.279 | **0.699** | 0.259889 |
| Sequence | 4 | I | B | 0.021 | **0.756** | 0.223 | 0.292222 |
| Sequence | 5 | I | B | 0.018 | **0.846** | 0.136 | 0.325444 |
| Sequence | 6 | G | B | 0.011 | **0.918** | 0.071 | 0.338444 |
| Sequence | 7 | I | B | 0.011 | **0.918** | 0.071 | 0.333444 |
| Sequence | 8 | D | B | 0.021 | **0.756** | 0.223 | 0.328333 |
| Sequence | 9 | L | B | 0.021 | 0.279 | **0.699** | 0.324889 |
| Sequence | 10 | G | B | 0.052 | 0.084 | **0.864** | 0.315 |
| Sequence | 11 | T | B | 0.053 | 0.043 | **0.903** | 0.311111 |
| Sequence | 12 | T | B | 0.056 | 0.142 | **0.802** | 0.319222 |
| Sequence | 13 | N | B | 0.022 | **0.552** | 0.426 | 0.322778 |
| Sequence | 14 | S | B | 0.002 | **0.816** | 0.182 | 0.327444 |
| Sequence | 15 | C | B | 0.001 | **0.959** | 0.04 | 0.326333 |
| Sequence | 16 | V | B | 0.001 | **0.959** | 0.04 | 0.343667 |
| Sequence | 17 | Y | B | 0 | **0.983** | 0.017 | 0.347889 |
| Sequence | 18 | V | B | 0 | **0.983** | 0.017 | 0.364556 |
| Sequence | 19 | M | B | 0.001 | **0.959** | 0.04 | 0.396667 |
| Sequence | 20 | E | E | 0.004 | **0.616** | 0.381 | 0.416444 |
| Sequence | 21 | G | E | 0.003 | 0.003 | **0.994** | 0.442556 |
| Sequence | 22 | K | E | 0.003 | 0.003 | **0.994** | 0.454 |
| Sequence | 23 | E | E | 0.004 | 0.42 | **0.576** | 0.445111 |
| Sequence | 24 | P | B | 0.003 | **0.718** | 0.279 | 0.458556 |
| Sequence | 25 | K | E | 0.001 | **0.959** | 0.04 | 0.453778 |
| Sequence | 26 | C | B | 0.001 | **0.959** | 0.04 | 0.47 |
| Sequence | 27 | I | B | 0.001 | **0.9** | 0.099 | 0.476222 |
| Sequence | 28 | T | E | 0.003 | **0.718** | 0.279 | 0.475333 |
| Sequence | 29 | N | B | 0.004 | 0.197 | **0.799** | **0.491111** |
| Sequence | 30 | P | E | 0.018 | 0.047 | **0.935** | **0.491** |
| Sequence | 31 | N | E | 0.018 | 0.019 | **0.964** | **0.495778** |
| Sequence | 32 | G | E | 0.018 | 0.019 | **0.964** | **0.512889** |
| Sequence | 33 | G | E | 0.005 | 0.045 | **0.951** | 0.482333 |
| Sequence | 34 | R | E | 0.005 | 0.262 | **0.733** | 0.459111 |
| Sequence | 35 | T | B | 0.004 | **0.514** | 0.481 | 0.411889 |
| Sequence | 36 | T | B | 0.004 | **0.514** | 0.481 | 0.371444 |
| Sequence | 37 | P | B | 0.004 | **0.514** | 0.481 | 0.345222 |
| Sequence | 38 | S | B | 0.001 | **0.9** | 0.099 | 0.316 |
| Sequence | 39 | V | B | 0.001 | **0.959** | 0.04 | 0.308556 |
| Sequence | 40 | V | B | 0.001 | **0.959** | 0.04 | 0.310444 |
| Sequence | 41 | A | B | 0.001 | **0.959** | 0.04 | 0.322889 |
| Sequence | 42 | F | B | 0.001 | **0.9** | 0.099 | 0.357889 |
| Sequence | 43 | T | E | 0.004 | 0.42 | **0.576** | 0.390111 |
| Sequence | 44 | D | E | 0.005 | 0.015 | **0.979** | 0.424222 |
| Sequence | 45 | K | E | 0.016 | 0.005 | **0.979** | 0.443111 |
| Sequence | 46 | D | E | 0.018 | 0.088 | **0.893** | 0.450222 |
| Sequence | 47 | R | E | 0.023 | **0.655** | 0.322 | 0.475778 |
| Sequence | 48 | L | B | 0.018 | **0.846** | 0.136 | 0.475667 |
| Sequence | 49 | V | B | 0.021 | **0.756** | 0.223 | 0.464556 |
| Sequence | 50 | G | B | 0.453 | 0.248 | 0.299 | 0.456667 |
| Sequence | 51 | D | E | **0.751** | 0.05 | 0.199 | 0.459111 |
| Sequence | 52 | A | E | **0.802** | 0.014 | 0.185 | 0.450333 |
| Sequence | 53 | A | B | **0.879** | 0.01 | 0.111 | 0.437778 |
| Sequence | 54 | K | E | **0.879** | 0.01 | 0.111 | 0.456667 |
| Sequence | 55 | R | E | **0.879** | 0.01 | 0.111 | 0.473778 |
| Sequence | 56 | Q | B | **0.782** | 0.003 | 0.216 | 0.47 |
| Sequence | 57 | A | B | **0.694** | 0.003 | 0.303 | 0.476333 |
| Sequence | 58 | I | E | **0.694** | 0.003 | 0.303 | **0.491333** |
| Sequence | 59 | T | E | **0.522** | 0.016 | 0.462 | **0.496222** |
| Sequence | 60 | N | E | 0.109 | 0.005 | **0.886** | 0.479 |
| Sequence | 61 | S | B | 0.109 | 0.005 | **0.886** | 0.459778 |
| Sequence | 62 | A | E | 0.321 | 0.003 | **0.675** | 0.466667 |
| Sequence | 63 | R | B | **0.622** | 0.015 | 0.363 | 0.438778 |
| Sequence | 64 | T | B | **0.831** | 0.044 | 0.125 | 0.403778 |
| Sequence | 65 | I | B | **0.87** | 0.077 | 0.053 | 0.402 |
| Sequence | 66 | F | B | **0.87** | 0.077 | 0.053 | 0.409444 |
| Sequence | 67 | A | B | **0.911** | 0.033 | 0.057 | 0.404889 |
| Sequence | 68 | V | B | **0.879** | 0.01 | 0.111 | 0.387556 |
| Sequence | 69 | K | B | **0.923** | 0.002 | 0.076 | 0.408333 |
| Sequence | 70 | R | E | **0.858** | 0.002 | 0.139 | 0.443444 |
| Sequence | 71 | L | B | **0.858** | 0.002 | 0.139 | 0.466444 |
| Sequence | 72 | M | B | **0.694** | 0.003 | 0.303 | 0.487556 |
| Sequence | 73 | G | E | 0.246 | 0.004 | **0.75** | **0.528333** |
| Sequence | 74 | R | E | 0.053 | 0.005 | **0.942** | **0.533222** |
| Sequence | 75 | R | E | 0.018 | 0.019 | **0.964** | **0.534** |
| Sequence | 76 | A | B | 0.018 | 0.019 | **0.964** | **0.543556** |
| Sequence | 77 | D | E | 0.016 | 0.005 | **0.979** | **0.559667** |
| Sequence | 78 | S | E | 0.176 | 0.004 | **0.82** | **0.569111** |
| Sequence | 79 | P | E | **0.782** | 0.003 | 0.216 | **0.558778** |
| Sequence | 80 | E | E | **0.782** | 0.003 | 0.216 | **0.547556** |
| Sequence | 81 | V | B | **0.802** | 0.014 | 0.185 | **0.560556** |
| Sequence | 82 | V | E | **0.879** | 0.01 | 0.111 | **0.569667** |
| Sequence | 83 | H | E | **0.802** | 0.014 | 0.185 | **0.571222** |
| Sequence | 84 | W | B | **0.802** | 0.014 | 0.185 | **0.561667** |
| Sequence | 85 | K | E | **0.717** | 0.014 | 0.269 | **0.558111** |
| Sequence | 86 | E | E | **0.522** | 0.016 | 0.462 | **0.565111** |
| Sequence | 87 | H | E | 0.339 | 0.016 | **0.645** | **0.552** |
| Sequence | 88 | A | B | 0.058 | 0.017 | **0.925** | **0.548** |
| Sequence | 89 | P | E | 0.018 | 0.047 | **0.935** | **0.540778** |
| Sequence | 90 | Y | B | 0.021 | 0.279 | **0.699** | **0.528556** |
| Sequence | 91 | K | E | 0.022 | **0.552** | 0.426 | **0.513** |
| Sequence | 92 | I | B | 0.021 | **0.756** | 0.223 | **0.515333** |
| Sequence | 93 | V | B | 0.021 | **0.756** | 0.223 | **0.521** |
| Sequence | 94 | A | E | 0.022 | **0.552** | 0.426 | **0.513667** |
| Sequence | 95 | A | E | 0.018 | 0.088 | **0.893** | **0.515444** |
| Sequence | 96 | S | E | 0.018 | 0.019 | **0.964** | **0.507222** |
| Sequence | 97 | N | E | 0.018 | 0.019 | **0.964** | **0.509** |
| Sequence | 98 | G | E | 0.018 | 0.047 | **0.935** | **0.509556** |
| Sequence | 99 | D | E | 0.022 | 0.359 | **0.619** | **0.507556** |
| Sequence | 100 | A | B | 0.021 | **0.756** | 0.223 | **0.506111** |
| Sequence | 101 | A | E | 0.018 | **0.846** | 0.136 | **0.511444** |
| Sequence | 102 | V | E | 0.018 | **0.846** | 0.136 | **0.508** |
| Sequence | 103 | E | E | 0.023 | **0.655** | 0.322 | **0.517556** |
| Sequence | 104 | I | B | 0.022 | 0.359 | **0.619** | **0.509222** |
| Sequence | 105 | D | E | 0.019 | 0.141 | **0.84** | **0.507111** |
| Sequence | 106 | G | E | 0.018 | 0.088 | **0.893** | **0.508889** |
| Sequence | 107 | R | E | 0.022 | 0.359 | **0.619** | **0.495889** |
| Sequence | 108 | E | E | 0.004 | **0.514** | 0.481 | **0.497333** |
| Sequence | 109 | Y | B | 0.022 | 0.411 | **0.567** | **0.495333** |
| Sequence | 110 | S | E | 0.004 | 0.085 | **0.91** | 0.461889 |
| Sequence | 111 | P | B | **0.923** | 0.002 | 0.076 | 0.434111 |
| Sequence | 112 | Q | E | **0.97** | 0.001 | 0.03 | 0.405333 |
| Sequence | 113 | E | B | **0.97** | 0.001 | 0.03 | 0.396111 |
| Sequence | 114 | I | B | **0.975** | 0.003 | 0.022 | 0.376556 |
| Sequence | 115 | S | B | **0.97** | 0.001 | 0.03 | 0.340556 |
| Sequence | 116 | A | B | **0.97** | 0.001 | 0.03 | 0.341667 |
| Sequence | 117 | I | B | **0.97** | 0.001 | 0.03 | 0.327 |
| Sequence | 118 | I | B | **0.988** | 0 | 0.012 | 0.300111 |
| Sequence | 119 | L | B | **0.988** | 0 | 0.012 | 0.305889 |
| Sequence | 120 | S | E | **0.988** | 0 | 0.012 | 0.328778 |
| Sequence | 121 | K | E | **0.988** | 0 | 0.012 | 0.337556 |
| Sequence | 122 | L | B | **0.97** | 0.001 | 0.03 | 0.333222 |
| Sequence | 123 | K | B | **0.97** | 0.001 | 0.03 | 0.361444 |
| Sequence | 124 | A | E | **0.97** | 0.001 | 0.03 | 0.397889 |
| Sequence | 125 | D | E | **0.97** | 0.001 | 0.03 | 0.414556 |
| Sequence | 126 | A | B | **0.97** | 0.001 | 0.03 | 0.425 |
| Sequence | 127 | E | E | **0.97** | 0.001 | 0.03 | 0.461667 |
| Sequence | 128 | A | E | **0.923** | 0.002 | 0.076 | 0.486556 |
| Sequence | 129 | Y | E | **0.858** | 0.002 | 0.139 | **0.493444** |
| Sequence | 130 | L | B | **0.502** | 0.002 | **0.495** | **0.500111** |
| Sequence | 131 | G | E | 0.053 | 0.005 | **0.942** | **0.511222** |
| Sequence | 132 | E | E | 0.016 | 0.005 | **0.979** | **0.503778** |
| Sequence | 133 | S | E | 0.018 | 0.047 | **0.935** | 0.477778 |
| Sequence | 134 | V | B | 0.004 | 0.085 | **0.91** | 0.447778 |
| Sequence | 135 | S | E | 0.004 | 0.085 | **0.91** | 0.410556 |
| Sequence | 136 | E | E | 0.005 | 0.262 | **0.733** | 0.372778 |
| Sequence | 137 | A | B | 0.002 | **0.816** | 0.182 | 0.335778 |
| Sequence | 138 | V | B | 0.001 | **0.959** | 0.04 | 0.326444 |
| Sequence | 139 | I | B | 0 | **0.983** | 0.017 | 0.334667 |
| Sequence | 140 | T | B | 0.001 | **0.959** | 0.04 | 0.353333 |
| Sequence | 141 | V | B | 0.002 | **0.816** | 0.182 | 0.357667 |
| Sequence | 142 | P | B | 0.005 | 0.262 | **0.733** | 0.389111 |
| Sequence | 143 | A | B | 0.004 | 0.085 | **0.91** | 0.43 |
| Sequence | 144 | Y | E | 0.018 | 0.088 | **0.893** | 0.476556 |
| Sequence | 145 | F | B | 0.018 | 0.047 | **0.935** | **0.510222** |
| Sequence | 146 | N | E | 0.018 | 0.019 | **0.964** | **0.531222** |
| Sequence | 147 | D | E | **0.923** | 0.002 | 0.076 | **0.552333** |
| Sequence | 148 | A | E | **0.97** | 0.001 | 0.03 | **0.541556** |
| Sequence | 149 | Q | B | **0.97** | 0.001 | 0.03 | **0.506778** |
| Sequence | 150 | R | B | **0.97** | 0.001 | 0.03 | **0.504222** |
| Sequence | 151 | Q | E | **0.97** | 0.001 | 0.03 | **0.494** |
| Sequence | 152 | A | B | **0.97** | 0.001 | 0.03 | 0.466333 |
| Sequence | 153 | T | B | **0.97** | 0.001 | 0.03 | 0.444222 |
| Sequence | 154 | K | E | **0.97** | 0.001 | 0.03 | 0.436556 |
| Sequence | 155 | D | E | **0.97** | 0.001 | 0.03 | 0.442333 |
| Sequence | 156 | A | B | **0.97** | 0.001 | 0.03 | 0.422111 |
| Sequence | 157 | G | B | **0.97** | 0.001 | 0.03 | 0.430667 |
| Sequence | 158 | R | E | **0.923** | 0.002 | 0.076 | 0.445889 |
| Sequence | 159 | I | E | **0.858** | 0.002 | 0.139 | 0.448111 |
| Sequence | 160 | A | B | 0.321 | 0.003 | **0.675** | 0.447556 |
| Sequence | 161 | G | E | 0.016 | 0.005 | **0.979** | 0.462778 |
| Sequence | 162 | L | B | 0.003 | 0.003 | **0.994** | 0.476 |
| Sequence | 163 | D | E | 0.018 | 0.047 | **0.935** | 0.461556 |
| Sequence | 164 | V | B | 0.079 | **0.592** | 0.329 | 0.441444 |
| Sequence | 165 | K | B | 0.087 | **0.683** | 0.23 | 0.439667 |
| Sequence | 166 | R | B | 0.085 | **0.775** | 0.14 | 0.427556 |
| Sequence | 167 | I | B | 0.085 | **0.775** | 0.14 | 0.417 |
| Sequence | 168 | I | B | 0.159 | **0.516** | 0.325 | 0.396556 |
| Sequence | 169 | N | B | 0.307 | 0.165 | **0.527** | 0.370444 |
| Sequence | 170 | E | B | 0.43 | 0.016 | **0.555** | 0.339222 |
| Sequence | 171 | P | B | **0.802** | 0.014 | 0.185 | 0.307556 |
| Sequence | 172 | T | B | **0.938** | 0.007 | 0.055 | 0.298111 |
| Sequence | 173 | A | B | **0.938** | 0.007 | 0.055 | 0.296444 |
| Sequence | 174 | A | B | **0.975** | 0.003 | 0.022 | 0.289111 |
| Sequence | 175 | S | B | **0.975** | 0.003 | 0.022 | 0.281778 |
| Sequence | 176 | L | B | **0.938** | 0.007 | 0.055 | 0.290222 |
| Sequence | 177 | A | B | **0.938** | 0.007 | 0.055 | 0.313 |
| Sequence | 178 | Y | B | **0.802** | 0.014 | 0.185 | 0.353778 |
| Sequence | 179 | G | B | **0.751** | 0.05 | 0.199 | 0.397556 |
| Sequence | 180 | F | B | **0.561** | 0.047 | 0.393 | 0.433889 |
| Sequence | 181 | D | E | 0.386 | 0.097 | **0.517** | 0.465111 |
| Sequence | 182 | K | E | 0.184 | 0.043 | **0.773** | 0.483667 |
| Sequence | 183 | K | E | 0.053 | 0.043 | **0.903** | **0.49** |
| Sequence | 184 | A | E | 0.018 | 0.019 | **0.964** | **0.499** |
| Sequence | 185 | N | E | 0.005 | 0.045 | **0.951** | 0.484222 |
| Sequence | 186 | E | E | 0.005 | 0.336 | **0.66** | 0.456556 |
| Sequence | 187 | K | B | 0.003 | **0.718** | 0.279 | 0.426333 |
| Sequence | 188 | I | B | 0.001 | **0.9** | 0.099 | 0.390333 |
| Sequence | 189 | A | B | 0.001 | **0.959** | 0.04 | 0.366778 |
| Sequence | 190 | V | B | 0 | **0.983** | 0.017 | 0.359556 |
| Sequence | 191 | F | B | 0.001 | **0.959** | 0.04 | 0.356 |
| Sequence | 192 | D | B | 0.001 | **0.9** | 0.099 | 0.359444 |
| Sequence | 193 | L | B | 0.004 | **0.514** | 0.481 | 0.364556 |
| Sequence | 194 | G | B | 0.004 | 0.085 | **0.91** | 0.374333 |
| Sequence | 195 | G | E | 0.003 | 0.003 | **0.994** | 0.392889 |
| Sequence | 196 | G | E | 0.005 | 0.045 | **0.951** | 0.384889 |
| Sequence | 197 | T | B | 0.004 | **0.616** | 0.381 | 0.387889 |
| Sequence | 198 | F | B | 0.001 | **0.9** | 0.099 | 0.390667 |
| Sequence | 199 | D | B | 0.001 | **0.9** | 0.099 | 0.368111 |
| Sequence | 200 | V | B | 0.001 | **0.959** | 0.04 | 0.350667 |
| Sequence | 201 | S | B | 0.001 | **0.959** | 0.04 | 0.334889 |
| Sequence | 202 | I | B | 0 | **0.983** | 0.017 | 0.337 |
| Sequence | 203 | L | B | 0 | **0.983** | 0.017 | 0.348222 |
| Sequence | 204 | E | B | 0 | **0.983** | 0.017 | 0.357333 |
| Sequence | 205 | V | B | 0.001 | **0.959** | 0.04 | 0.375667 |
| Sequence | 206 | G | E | 0.005 | 0.336 | **0.66** | 0.366222 |
| Sequence | 207 | D | E | 0.003 | 0.003 | **0.994** | 0.365111 |
| Sequence | 208 | S | E | 0.005 | 0.045 | **0.951** | 0.35 |
| Sequence | 209 | V | E | 0.002 | **0.816** | 0.182 | 0.350444 |
| Sequence | 210 | V | B | 0.001 | **0.959** | 0.04 | 0.334222 |
| Sequence | 211 | E | B | 0.001 | **0.959** | 0.04 | 0.317778 |
| Sequence | 212 | V | B | 0.001 | **0.959** | 0.04 | 0.308556 |
| Sequence | 213 | L | B | 0.001 | **0.959** | 0.04 | 0.298333 |
| Sequence | 214 | A | B | 0.001 | **0.9** | 0.099 | 0.304 |
| Sequence | 215 | T | B | 0.002 | **0.816** | 0.182 | 0.341556 |
| Sequence | 216 | N | B | 0.004 | 0.42 | **0.576** | 0.365667 |
| Sequence | 217 | G | B | 0.018 | 0.088 | **0.893** | 0.405556 |
| Sequence | 218 | D | B | 0.018 | 0.047 | **0.935** | 0.422444 |
| Sequence | 219 | T | B | 0.053 | 0.043 | **0.903** | 0.451889 |
| Sequence | 220 | F | E | 0.053 | 0.043 | **0.903** | 0.468556 |
| Sequence | 221 | L | B | 0.053 | 0.043 | **0.903** | 0.475667 |
| Sequence | 222 | G | B | 0.115 | 0.016 | **0.868** | 0.476667 |
| Sequence | 223 | G | B | **0.782** | 0.003 | 0.216 | 0.471333 |
| Sequence | 224 | E | E | **0.858** | 0.002 | 0.139 | 0.462111 |
| Sequence | 225 | D | B | **0.923** | 0.002 | 0.076 | 0.455333 |
| Sequence | 226 | F | B | **0.97** | 0.001 | 0.03 | 0.441333 |
| Sequence | 227 | D | B | **0.97** | 0.001 | 0.03 | 0.435667 |
| Sequence | 228 | Q | E | **0.97** | 0.001 | 0.03 | 0.447333 |
| Sequence | 229 | R | B | **0.97** | 0.001 | 0.03 | 0.448 |
| Sequence | 230 | I | B | **0.97** | 0.001 | 0.03 | 0.426222 |
| Sequence | 231 | I | B | **0.97** | 0.001 | 0.03 | 0.423778 |
| Sequence | 232 | N | E | **0.97** | 0.001 | 0.03 | 0.441333 |
| Sequence | 233 | Y | B | **0.923** | 0.002 | 0.076 | 0.437222 |
| Sequence | 234 | L | B | **0.923** | 0.002 | 0.076 | 0.418222 |
| Sequence | 235 | V | B | **0.97** | 0.001 | 0.03 | 0.450667 |
| Sequence | 236 | E | E | **0.97** | 0.001 | 0.03 | 0.465667 |
| Sequence | 237 | E | E | **0.97** | 0.001 | 0.03 | 0.474778 |
| Sequence | 238 | F | B | **0.923** | 0.002 | 0.076 | **0.493** |
| Sequence | 239 | K | E | **0.923** | 0.002 | 0.076 | **0.528556** |
| Sequence | 240 | K | E | **0.858** | 0.002 | 0.139 | **0.537333** |
| Sequence | 241 | E | E | **0.782** | 0.003 | 0.216 | **0.537556** |
| Sequence | 242 | Q | E | 0.406 | 0.004 | **0.59** | **0.547778** |
| Sequence | 243 | G | E | 0.053 | 0.005 | **0.942** | **0.576444** |
| Sequence | 244 | I | B | 0.016 | 0.005 | **0.979** | **0.567** |
| Sequence | 245 | D | E | 0.005 | 0.015 | **0.979** | **0.578333** |
| Sequence | 246 | L | B | 0.058 | 0.017 | **0.925** | **0.577889** |
| Sequence | 247 | S | E | 0.257 | 0.016 | **0.727** | **0.568556** |
| Sequence | 248 | K | E | 0.257 | 0.016 | **0.727** | **0.553778** |
| Sequence | 249 | D | E | 0.181 | 0.016 | **0.803** | **0.546778** |
| Sequence | 250 | N | E | **0.923** | 0.002 | 0.076 | **0.546444** |
| Sequence | 251 | M | E | **0.97** | 0.001 | 0.03 | **0.549** |
| Sequence | 252 | A | B | **0.97** | 0.001 | 0.03 | **0.524556** |
| Sequence | 253 | L | B | **0.97** | 0.001 | 0.03 | **0.511778** |
| Sequence | 254 | Q | E | **0.97** | 0.001 | 0.03 | **0.508** |
| Sequence | 255 | R | E | **0.97** | 0.001 | 0.03 | 0.488444 |
| Sequence | 256 | L | B | **0.97** | 0.001 | 0.03 | 0.463222 |
| Sequence | 257 | K | B | **0.988** | 0 | 0.012 | 0.47 |
| Sequence | 258 | D | E | **0.988** | 0 | 0.012 | 0.469556 |
| Sequence | 259 | S | B | **0.988** | 0 | 0.012 | 0.451333 |
| Sequence | 260 | A | B | **0.97** | 0.001 | 0.03 | 0.437444 |
| Sequence | 261 | E | E | **0.97** | 0.001 | 0.03 | 0.462889 |
| Sequence | 262 | N | E | **0.97** | 0.001 | 0.03 | 0.471889 |
| Sequence | 263 | A | B | **0.923** | 0.002 | 0.076 | 0.443889 |
| Sequence | 264 | K | B | **0.782** | 0.003 | 0.216 | 0.459333 |
| Sequence | 265 | K | E | **0.802** | 0.014 | 0.185 | **0.493222** |
| Sequence | 266 | E | E | **0.802** | 0.014 | 0.185 | **0.501222** |
| Sequence | 267 | L | B | **0.522** | 0.016 | 0.462 | **0.514333** |
| Sequence | 268 | S | B | 0.181 | 0.016 | **0.803** | **0.534556** |
| Sequence | 269 | T | E | 0.115 | 0.016 | **0.868** | **0.536556** |
| Sequence | 270 | A | E | 0.053 | 0.043 | **0.903** | **0.530333** |
| Sequence | 271 | M | E | 0.052 | 0.084 | **0.864** | **0.512** |
| Sequence | 272 | E | E | 0.021 | 0.279 | **0.699** | **0.518556** |
| Sequence | 273 | T | B | 0.004 | **0.514** | 0.481 | 0.489 |
| Sequence | 274 | E | E | 0.002 | **0.816** | 0.182 | 0.46 |
| Sequence | 275 | I | B | 0.001 | **0.9** | 0.099 | 0.434556 |
| Sequence | 276 | N | E | 0.001 | **0.9** | 0.099 | 0.416889 |
| Sequence | 277 | L | B | 0.002 | **0.816** | 0.182 | 0.396222 |
| Sequence | 278 | P | E | 0.021 | **0.756** | 0.223 | 0.392556 |
| Sequence | 279 | F | B | 0.018 | **0.846** | 0.136 | 0.394556 |
| Sequence | 280 | I | B | 0.018 | **0.846** | 0.136 | 0.421333 |
| Sequence | 281 | T | B | 0.021 | **0.756** | 0.223 | 0.449444 |
| Sequence | 282 | A | E | 0.022 | **0.552** | 0.426 | 0.486 |
| Sequence | 283 | D | E | 0.02 | 0.205 | **0.775** | **0.506222** |
| Sequence | 284 | Q | E | 0.018 | 0.088 | **0.893** | **0.524444** |
| Sequence | 285 | S | E | 0.018 | 0.088 | **0.893** | **0.538556** |
| Sequence | 286 | G | E | 0.018 | 0.047 | **0.935** | **0.540111** |
| Sequence | 287 | P | E | 0.004 | 0.085 | **0.91** | **0.536222** |
| Sequence | 288 | K | E | 0.005 | 0.262 | **0.733** | **0.519** |
| Sequence | 289 | H | E | 0.004 | **0.616** | 0.381 | 0.**498333** |
| Sequence | 290 | L | B | 0.018 | **0.846** | 0.136 | 0.458889 |
| Sequence | 291 | L | E | 0.011 | **0.918** | 0.071 | 0.440111 |
| Sequence | 292 | I | B | 0.006 | **0.962** | 0.032 | 0.428778 |
| Sequence | 293 | K | E | 0.001 | **0.9** | 0.099 | 0.430111 |
| Sequence | 294 | L | B | 0.003 | **0.718** | 0.279 | 0.431222 |
| Sequence | 295 | T | E | 0.021 | 0.279 | **0.699** | 0.431444 |
| Sequence | 296 | R | B | **0.938** | 0.007 | 0.055 | 0.442444 |
| Sequence | 297 | A | E | **0.97** | 0.001 | 0.03 | 0.458556 |
| Sequence | 298 | K | E | **0.97** | 0.001 | 0.03 | 0.445778 |
| Sequence | 299 | L | B | **0.97** | 0.001 | 0.03 | 0.445667 |
| Sequence | 300 | E | E | **0.97** | 0.001 | 0.03 | 0.467111 |
| Sequence | 301 | Q | E | **0.97** | 0.001 | 0.03 | 0.473556 |
| Sequence | 302 | L | B | **0.97** | 0.001 | 0.03 | 0.441889 |
| Sequence | 303 | V | B | **0.923** | 0.002 | 0.076 | 0.427556 |
| Sequence | 304 | M | E | **0.923** | 0.002 | 0.076 | 0.44 |
| Sequence | 305 | D | E | **0.858** | 0.002 | 0.139 | 0.439111 |
| Sequence | 306 | L | B | **0.858** | 0.002 | 0.139 | 0.426889 |
| Sequence | 307 | V | B | **0.923** | 0.002 | 0.076 | 0.455444 |
| Sequence | 308 | G | E | **0.923** | 0.002 | 0.076 | 0.487444 |
| Sequence | 309 | R | E | **0.923** | 0.002 | 0.076 | 0.457667 |
| Sequence | 310 | T | B | **0.97** | 0.001 | 0.03 | 0.433444 |
| Sequence | 311 | I | B | **0.97** | 0.001 | 0.03 | 0.458444 |
| Sequence | 312 | E | E | **0.97** | 0.001 | 0.03 | 0.471 |
| Sequence | 313 | P | B | **0.97** | 0.001 | 0.03 | 0.460222 |
| Sequence | 314 | C | B | **0.97** | 0.001 | 0.03 | 0.440778 |
| Sequence | 315 | S | E | **0.97** | 0.001 | 0.03 | 0.444556 |
| Sequence | 316 | K | E | **0.97** | 0.001 | 0.03 | 0.443778 |
| Sequence | 317 | A | B | **0.97** | 0.001 | 0.03 | 0.436222 |
| Sequence | 318 | L | B | **0.923** | 0.002 | 0.076 | 0.461444 |
| Sequence | 319 | E | E | **0.858** | 0.002 | 0.139 | 0.489222 |
| Sequence | 320 | D | E | **0.782** | 0.003 | 0.216 | **0.494889** |
| Sequence | 321 | A | B | 0.406 | 0.004 | **0.59** | **0.496222** |
| Sequence | 322 | G | E | 0.109 | 0.005 | **0.886** | **0.509778** |
| Sequence | 323 | L | E | 0.053 | 0.005 | **0.942** | **0.536667** |
| Sequence | 324 | Q | E | 0.018 | 0.019 | **0.964** | **0.536222** |
| Sequence | 325 | T | E | 0.181 | 0.016 | **0.803** | **0.529444** |
| Sequence | 326 | S | E | 0.115 | 0.016 | **0.868** | **0.518** |
| Sequence | 327 | N | E | 0.053 | 0.043 | **0.903** | 0.482111 |
| Sequence | 328 | I | B | 0.052 | 0.084 | **0.864** | 0.453556 |
| Sequence | 329 | D | E | 0.019 | 0.141 | **0.84** | 0.417333 |
| Sequence | 330 | E | B | 0.021 | **0.756** | 0.223 | 0.385444 |
| Sequence | 331 | V | B | 0.011 | **0.918** | 0.071 | 0.351778 |
| Sequence | 332 | I | B | 0.006 | **0.962** | 0.032 | 0.321778 |
| Sequence | 333 | L | B | 0.011 | **0.918** | 0.071 | 0.314889 |
| Sequence | 334 | V | B | 0.022 | **0.552** | 0.426 | 0.308 |
| Sequence | 335 | G | B | 0.02 | 0.205 | **0.775** | 0.314222 |
| Sequence | 336 | G | B | 0.052 | 0.084 | **0.864** | 0.331556 |
| Sequence | 337 | M | B | 0.113 | 0.043 | **0.844** | 0.358667 |
| Sequence | 338 | T | B | 0.115 | 0.016 | **0.868** | 0.389778 |
| Sequence | 339 | R | B | 0.181 | 0.016 | **0.803** | 0.391556 |
| Sequence | 340 | M | B | 0.406 | 0.004 | **0.59** | 0.417667 |
| Sequence | 341 | P | E | **0.858** | 0.002 | 0.139 | 0.448556 |
| Sequence | 342 | L | B | **0.923** | 0.002 | 0.076 | 0.461889 |
| Sequence | 343 | V | B | **0.97** | 0.001 | 0.03 | 0.457333 |
| Sequence | 344 | Q | B | **0.97** | 0.001 | 0.03 | 0.462444 |
| Sequence | 345 | K | E | **0.988** | 0 | 0.012 | 0.468444 |
| Sequence | 346 | K | B | **0.988** | 0 | 0.012 | 0.472 |
| Sequence | 347 | V | B | **0.988** | 0 | 0.012 | 0.465 |
| Sequence | 348 | A | E | **0.97** | 0.001 | 0.03 | **0.505778** |
| Sequence | 349 | E | E | **0.97** | 0.001 | 0.03 | **0.519333** |
| Sequence | 350 | F | E | **0.858** | 0.002 | 0.139 | **0.520556** |
| Sequence | 351 | F | B | **0.502** | 0.002 | **0.495** | **0.523556** |
| Sequence | 352 | G | E | 0.016 | 0.005 | **0.979** | **0.544444** |
| Sequence | 353 | K | E | 0.018 | 0.019 | **0.964** | **0.568333** |
| Sequence | 354 | E | E | 0.004 | 0.085 | **0.91** | **0.577** |
| Sequence | 355 | P | B | 0.005 | 0.262 | **0.733** | **0.578111** |
| Sequence | 356 | N | E | 0.005 | 0.336 | **0.66** | **0.590667** |
| Sequence | 357 | R | E | 0.021 | 0.279 | **0.699** | **0.568556** |
| Sequence | 358 | S | E | 0.019 | 0.141 | **0.84** | **0.560333** |
| Sequence | 359 | V | B | 0.018 | 0.088 | **0.893** | **0.551778** |
| Sequence | 360 | N | B | 0.018 | 0.019 | **0.964** | **0.525556** |
| Sequence | 361 | P | B | 0.115 | 0.016 | **0.868** | **0.494778** |
| Sequence | 362 | D | E | 0.257 | 0.016 | **0.727** | 0.436556 |
| Sequence | 363 | E | B | 0.455 | 0.046 | **0.498** | 0.408 |
| Sequence | 364 | V | B | **0.538** | 0.173 | 0.289 | 0.368667 |
| Sequence | 365 | V | B | **0.538** | 0.173 | 0.289 | 0.334556 |
| Sequence | 366 | A | B | **0.538** | 0.173 | 0.289 | 0.317111 |
| Sequence | 367 | M | B | **0.502** | 0.102 | 0.396 | 0.288667 |
| Sequence | 368 | G | B | **0.502** | 0.102 | 0.396 | 0.278556 |
| Sequence | 369 | A | B | 0.428 | 0.171 | 0.402 | 0.281667 |
| Sequence | 370 | A | B | 0.321 | 0.252 | 0.427 | 0.296 |
| Sequence | 371 | I | B | 0.352 | 0.332 | 0.316 | 0.320667 |
| Sequence | 372 | Q | B | 0.453 | 0.248 | 0.299 | 0.34 |
| Sequence | 373 | G | B | **0.502** | 0.102 | 0.396 | 0.378111 |
| Sequence | 374 | G | B | **0.502** | 0.102 | 0.396 | 0.418889 |
| Sequence | 375 | I | E | **0.502** | 0.102 | 0.396 | 0.459222 |
| Sequence | 376 | L | B | 0.455 | 0.046 | **0.498** | **0.491556** |
| Sequence | 377 | A | E | 0.268 | 0.043 | **0.689** | **0.517444** |
| Sequence | 378 | G | E | 0.184 | 0.043 | **0.773** | **0.542** |
| Sequence | 379 | D | E | 0.191 | 0.086 | **0.723** | **0.548778** |
| Sequence | 380 | V | E | 0.113 | 0.087 | **0.8** | **0.567444** |
| Sequence | 381 | K | E | 0.118 | 0.15 | **0.732** | **0.57** |
| Sequence | 382 | D | E | 0.066 | 0.296 | **0.638** | **0.566667** |
| Sequence | 383 | V | B | 0.069 | 0.386 | **0.545** | **0.559556** |
| Sequence | 384 | L | E | 0.074 | 0.484 | 0.442 | **0.543222** |
| Sequence | 385 | L | E | 0.074 | 0.484 | 0.442 | **0.535333** |
| Sequence | 386 | L | B | 0.021 | 0.451 | **0.528** | **0.520111** |
| Sequence | 387 | D | E | 0.021 | 0.451 | **0.528** | **0.512889** |
| Sequence | 388 | V | B | 0.021 | 0.451 | **0.528** | **0.513778** |
| Sequence | 389 | T | B | 0.022 | 0.359 | **0.619** | **0.499778** |
| Sequence | 390 | P | B | 0.022 | 0.359 | **0.619** | **0.492556** |
| Sequence | 391 | L | B | 0.069 | 0.386 | **0.545** | 0.489556 |
| Sequence | 392 | S | B | 0.074 | 0.484 | 0.442 | **0.493889** |
| Sequence | 393 | L | B | 0.074 | 0.484 | 0.442 | **0.5** |
| Sequence | 394 | G | B | 0.069 | 0.386 | **0.545** | **0.508222** |
| Sequence | 395 | I | B | 0.148 | 0.418 | 0.435 | **0.521444** |
| Sequence | 396 | E | E | 0.135 | 0.317 | **0.548** | **0.527778** |
| Sequence | 397 | T | B | 0.216 | 0.235 | **0.548** | **0.533111** |
| Sequence | 398 | M | E | 0.113 | 0.087 | **0.8** | **0.544111** |
| Sequence | 399 | G | E | 0.053 | 0.043 | **0.903** | **0.554667** |
| Sequence | 400 | G | E | 0.056 | 0.142 | **0.802** | **0.558333** |
| Sequence | 401 | V | E | 0.022 | 0.359 | **0.619** | **0.547222** |
| Sequence | 402 | F | B | 0.021 | 0.451 | **0.528** | **0.526778** |
| Sequence | 403 | T | E | 0.023 | **0.655** | 0.322 | **0.530333** |
| Sequence | 404 | K | E | 0.021 | **0.756** | 0.223 | **0.534222** |
| Sequence | 405 | L | B | 0.023 | **0.655** | 0.322 | **0.542556** |
| Sequence | 406 | I | B | 0.023 | **0.655** | 0.322 | **0.549111** |
| Sequence | 407 | D | E | 0.021 | 0.451 | **0.528** | **0.556667** |
| Sequence | 408 | R | E | 0.019 | 0.141 | **0.84** | **0.570333** |
| Sequence | 409 | N | E | 0.018 | 0.047 | **0.935** | **0.582** |
| Sequence | 410 | T | B | 0.018 | 0.088 | **0.893** | **0.593333** |
| Sequence | 411 | T | E | 0.02 | 0.205 | **0.775** | **0.631** |
| Sequence | 412 | I | E | 0.019 | 0.141 | **0.84** | **0.631778** |
| Sequence | 413 | P | E | 0.019 | 0.141 | **0.84** | **0.616333** |
| Sequence | 414 | T | E | 0.022 | 0.359 | **0.619** | **0.602333** |
| Sequence | 415 | R | E | 0.021 | 0.451 | **0.528** | **0.581333** |
| Sequence | 416 | K | E | 0.004 | 0.42 | **0.576** | **0.545111** |
| Sequence | 417 | S | E | 0.004 | **0.514** | 0.481 | **0.522556** |
| Sequence | 418 | Q | B | 0.002 | **0.816** | 0.182 | **0.496556** |
| Sequence | 419 | T | B | 0.001 | **0.9** | 0.099 | 0.485222 |
| Sequence | 420 | F | B | 0.001 | **0.9** | 0.099 | 0.468556 |
| Sequence | 421 | T | E | 0.002 | **0.816** | 0.182 | 0.467222 |
| Sequence | 422 | T | B | 0.003 | **0.718** | 0.279 | 0.472556 |
| Sequence | 423 | A | B | 0.005 | 0.336 | **0.66** | 0.483222 |
| Sequence | 424 | A | E | 0.004 | 0.197 | **0.799** | **0.499333** |
| Sequence | 425 | D | E | 0.004 | 0.085 | **0.91** | **0.529111** |
| Sequence | 426 | N | E | 0.018 | 0.047 | **0.935** | **0.506889** |
| Sequence | 427 | Q | E | 0.004 | 0.085 | **0.91** | **0.505333** |
| Sequence | 428 | P | E | 0.004 | 0.42 | **0.576** | 0.472111 |
| Sequence | 429 | S | E | 0.002 | **0.816** | 0.182 | 0.454222 |
| Sequence | 430 | V | B | 0.001 | **0.9** | 0.099 | 0.424778 |
| Sequence | 431 | S | B | 0.001 | **0.959** | 0.04 | 0.414 |
| Sequence | 432 | I | B | 0.001 | **0.959** | 0.04 | 0.417778 |
| Sequence | 433 | H | E | 0.001 | **0.9** | 0.099 | 0.418778 |
| Sequence | 434 | V | B | 0.021 | **0.756** | 0.223 | 0.432444 |
| Sequence | 435 | L | B | 0.022 | **0.552** | 0.426 | 0.486556 |
| Sequence | 436 | Q | E | 0.066 | 0.296 | **0.638** | **0.52** |
| Sequence | 437 | G | E | 0.052 | 0.084 | **0.864** | **0.578111** |
| Sequence | 438 | E | E | 0.052 | 0.084 | **0.864** | **0.606222** |
| Sequence | 439 | R | E | 0.052 | 0.084 | **0.864** | **0.641444** |
| Sequence | 440 | P | E | 0.053 | 0.043 | **0.903** | **0.652667** |
| Sequence | 441 | M | E | 0.113 | 0.043 | **0.844** | **0.643778** |
| Sequence | 442 | A | E | 0.191 | 0.086 | **0.723** | **0.636444** |
| Sequence | 443 | S | E | 0.191 | 0.086 | **0.723** | **0.615** |
| Sequence | 444 | D | E | 0.113 | 0.087 | **0.8** | **0.572444** |
| Sequence | 445 | N | B | 0.113 | 0.087 | **0.8** | **0.536889** |
| Sequence | 446 | M | E | 0.066 | 0.296 | **0.638** | **0.509222** |
| Sequence | 447 | T | E | 0.074 | 0.484 | 0.442 | 0.471778 |
| Sequence | 448 | L | B | 0.022 | **0.552** | 0.426 | 0.443333 |
| Sequence | 449 | A | B | 0.021 | **0.756** | 0.223 | 0.413111 |
| Sequence | 450 | R | E | 0.011 | **0.918** | 0.071 | 0.418444 |
| Sequence | 451 | F | B | 0.011 | **0.918** | 0.071 | 0.423111 |
| Sequence | 452 | D | E | 0.018 | **0.846** | 0.136 | 0.417889 |
| Sequence | 453 | L | B | 0.004 | **0.616** | 0.381 | 0.447556 |
| Sequence | 454 | T | E | 0.004 | **0.514** | 0.481 | 0.472889 |
| Sequence | 455 | G | E | 0.005 | 0.336 | **0.66** | 0.484444 |
| Sequence | 456 | I | B | 0.005 | 0.262 | **0.733** | **0.522333** |
| Sequence | 457 | P | E | 0.019 | 0.141 | **0.84** | **0.555667** |
| Sequence | 458 | P | E | 0.018 | 0.088 | **0.893** | **0.579** |
| Sequence | 459 | A | E | 0.018 | 0.019 | **0.964** | **0.568667** |
| Sequence | 460 | P | E | 0.018 | 0.019 | **0.964** | **0.563889** |
| Sequence | 461 | R | E | 0.018 | 0.047 | **0.935** | **0.573667** |
| Sequence | 462 | G | E | 0.004 | 0.085 | **0.91** | **0.531** |
| Sequence | 463 | V | E | 0.004 | 0.197 | **0.799** | **0.507778** |
| Sequence | 464 | P | E | 0.004 | **0.514** | 0.481 | 0.476333 |
| Sequence | 465 | Q | E | 0.001 | **0.9** | 0.099 | 0.443667 |
| Sequence | 466 | I | B | 0.001 | **0.959** | 0.04 | 0.396444 |
| Sequence | 467 | E | E | 0.001 | **0.959** | 0.04 | 0.381444 |
| Sequence | 468 | V | B | 0.001 | **0.959** | 0.04 | 0.365667 |
| Sequence | 469 | A | B | 0.001 | **0.959** | 0.04 | 0.361333 |
| Sequence | 470 | F | B | 0.001 | **0.959** | 0.04 | 0.350222 |
| Sequence | 471 | N | E | 0.001 | **0.959** | 0.04 | 0.378333 |
| Sequence | 472 | I | B | 0.001 | **0.9** | 0.099 | 0.375333 |
| Sequence | 473 | D | E | 0.005 | 0.262 | **0.733** | 0.393778 |
| Sequence | 474 | A | E | 0.005 | 0.045 | **0.951** | 0.378 |
| Sequence | 475 | N | E | 0.005 | 0.045 | **0.951** | 0.384111 |
| Sequence | 476 | G | B | 0.004 | **0.514** | 0.481 | 0.378556 |
| Sequence | 477 | I | B | 0.001 | **0.9** | 0.099 | 0.385667 |
| Sequence | 478 | V | B | 0.001 | **0.959** | 0.04 | 0.369889 |
| Sequence | 479 | N | B | 0.001 | **0.959** | 0.04 | 0.380444 |
| Sequence | 480 | V | B | 0.001 | **0.959** | 0.04 | 0.387444 |
| Sequence | 481 | S | E | 0.001 | **0.9** | 0.099 | 0.415778 |
| Sequence | 482 | A | B | 0.011 | **0.918** | 0.071 | 0.433 |
| Sequence | 483 | K | E | 0.001 | **0.9** | 0.099 | 0.478444 |
| Sequence | 484 | D | E | 0.003 | **0.718** | 0.279 | **0.508333** |
| Sequence | 485 | L | E | 0.021 | 0.451 | **0.528** | **0.534** |
| Sequence | 486 | G | E | 0.02 | 0.205 | **0.775** | **0.552** |
| Sequence | 487 | T | E | 0.019 | 0.141 | **0.84** | **0.573** |
| Sequence | 488 | G | E | 0.005 | 0.262 | **0.733** | **0.565778** |
| Sequence | 489 | K | E | 0.004 | **0.514** | 0.481 | **0.557333** |
| Sequence | 490 | E | E | 0.002 | **0.816** | 0.182 | **0.543778** |
| Sequence | 491 | Q | E | 0.002 | **0.816** | 0.182 | **0.521444** |
| Sequence | 492 | S | E | 0.002 | **0.816** | 0.182 | **0.520444** |
| Sequence | 493 | I | E | 0.001 | **0.9** | 0.099 | **0.523889** |
| Sequence | 494 | Q | E | 0.001 | **0.9** | 0.099 | **0.531889** |
| Sequence | 495 | I | B | 0.018 | **0.846** | 0.136 | **0.543** |
| Sequence | 496 | T | E | 0.023 | **0.655** | 0.322 | **0.559556** |
| Sequence | 497 | A | E | 0.022 | 0.359 | **0.619** | **0.574111** |
| Sequence | 498 | S | E | 0.056 | 0.142 | **0.802** | **0.580222** |
| Sequence | 499 | S | E | 0.056 | 0.142 | **0.802** | **0.589667** |
| Sequence | 500 | G | E | 0.118 | 0.15 | **0.732** | **0.603667** |
| Sequence | 501 | L | E | 0.199 | 0.152 | **0.649** | **0.605444** |
| Sequence | 502 | S | E | 0.278 | 0.093 | **0.628** | **0.586778** |
| Sequence | 503 | E | E | **0.622** | 0.015 | 0.363 | **0.586667** |
| Sequence | 504 | A | E | **0.879** | 0.01 | 0.111 | **0.574444** |
| Sequence | 505 | D | E | **0.923** | 0.002 | 0.076 | **0.560556** |
| Sequence | 506 | I | B | **0.923** | 0.002 | 0.076 | **0.558778** |
| Sequence | 507 | E | E | **0.97** | 0.001 | 0.03 | **0.567333** |
| Sequence | 508 | K | E | **0.97** | 0.001 | 0.03 | **0.546889** |
| Sequence | 509 | L | B | **0.923** | 0.002 | 0.076 | **0.527889** |
| Sequence | 510 | I | B | **0.923** | 0.002 | 0.076 | **0.529889** |
| Sequence | 511 | R | E | **0.923** | 0.002 | 0.076 | **0.533778** |
| Sequence | 512 | E | E | **0.923** | 0.002 | 0.076 | **0.521667** |
| Sequence | 513 | A | B | **0.923** | 0.002 | 0.076 | **0.511333** |
| Sequence | 514 | E | E | **0.858** | 0.002 | 0.139 | **0.515333** |
| Sequence | 515 | S | E | **0.782** | 0.003 | 0.216 | **0.509111** |
| Sequence | 516 | H | B | **0.6** | 0.003 | 0.397 | **0.501111** |
| Sequence | 517 | A | E | **0.694** | 0.003 | 0.303 | **0.523778** |
| Sequence | 518 | S | E | **0.694** | 0.003 | 0.303 | **0.549333** |
| Sequence | 519 | E | E | **0.6** | 0.003 | 0.397 | **0.544889** |
| Sequence | 520 | D | E | **0.782** | 0.003 | 0.216 | **0.555889** |
| Sequence | 521 | K | E | **0.923** | 0.002 | 0.076 | **0.563** |
| Sequence | 522 | K | E | **0.97** | 0.001 | 0.03 | **0.578222** |
| Sequence | 523 | K | E | **0.923** | 0.002 | 0.076 | **0.584111** |
| Sequence | 524 | Q | E | **0.97** | 0.001 | 0.03 | **0.584111** |
| Sequence | 525 | E | E | **0.923** | 0.002 | 0.076 | **0.582444** |
| Sequence | 526 | I | B | **0.97** | 0.001 | 0.03 | **0.577444** |
| Sequence | 527 | I | B | **0.97** | 0.001 | 0.03 | **0.572778** |
| Sequence | 528 | E | E | **0.97** | 0.001 | 0.03 | **0.568556** |
| Sequence | 529 | V | B | **0.97** | 0.001 | 0.03 | **0.544** |
| Sequence | 530 | R | E | **0.97** | 0.001 | 0.03 | **0.531222** |
| Sequence | 531 | N | E | **0.97** | 0.001 | 0.03 | **0.514778** |
| Sequence | 532 | H | E | **0.97** | 0.001 | 0.03 | **0.495444** |
| Sequence | 533 | A | B | **0.97** | 0.001 | 0.03 | 0.469778 |
| Sequence | 534 | D | E | **0.97** | 0.001 | 0.03 | 0.47 |
| Sequence | 535 | G | E | **0.97** | 0.001 | 0.03 | 0.455556 |
| Sequence | 536 | L | B | **0.97** | 0.001 | 0.03 | 0.438333 |
| Sequence | 537 | I | B | **0.97** | 0.001 | 0.03 | 0.436222 |
| Sequence | 538 | Y | E | **0.97** | 0.001 | 0.03 | 0.454667 |
| Sequence | 539 | S | E | **0.97** | 0.001 | 0.03 | 0.458556 |
| Sequence | 540 | T | B | **0.97** | 0.001 | 0.03 | 0.463556 |
| Sequence | 541 | E | E | **0.97** | 0.001 | 0.03 | 0.480222 |
| Sequence | 542 | K | E | **0.97** | 0.001 | 0.03 | **0.504222** |
| Sequence | 543 | S | B | **0.923** | 0.002 | 0.076 | **0.502222** |
| Sequence | 544 | I | B | **0.923** | 0.002 | 0.076 | **0.520778** |
| Sequence | 545 | K | E | **0.858** | 0.002 | 0.139 | **0.537556** |
| Sequence | 546 | D | E | **0.782** | 0.003 | 0.216 | **0.540667** |
| Sequence | 547 | L | B | **0.502** | 0.002 | **0.495** | **0.528** |
| Sequence | 548 | E | E | 0.406 | 0.004 | **0.59** | **0.531222** |
| Sequence | 549 | G | E | 0.321 | 0.003 | **0.675** | **0.530778** |
| Sequence | 550 | K | E | 0.246 | 0.004 | **0.75** | **0.522222** |
| Sequence | 551 | I | B | 0.018 | 0.019 | **0.964** | **0.518889** |
| Sequence | 552 | D | E | 0.018 | 0.019 | **0.964** | **0.512889** |
| Sequence | 553 | A | E | **0.923** | 0.002 | 0.076 | **0.495333** |
| Sequence | 554 | E | E | **0.97** | 0.001 | 0.03 | **0.493556** |
| Sequence | 555 | L | B | **0.97** | 0.001 | 0.03 | 0.481 |
| Sequence | 556 | Q | B | **0.97** | 0.001 | 0.03 | 0.497444 |
| Sequence | 557 | A | E | **0.97** | 0.001 | 0.03 | 0.486889 |
| Sequence | 558 | D | E | **0.97** | 0.001 | 0.03 | 0.483111 |
| Sequence | 559 | I | B | **0.97** | 0.001 | 0.03 | 0.461111 |
| Sequence | 560 | T | E | **0.97** | 0.001 | 0.03 | 0.471667 |
| Sequence | 561 | S | E | **0.97** | 0.001 | 0.03 | 0.473667 |
| Sequence | 562 | K | B | **0.97** | 0.001 | 0.03 | 0.460667 |
| Sequence | 563 | I | B | **0.97** | 0.001 | 0.03 | 0.470667 |
| Sequence | 564 | E | E | **0.97** | 0.001 | 0.03 | 0.480778 |
| Sequence | 565 | A | E | **0.97** | 0.001 | 0.03 | 0.461444 |
| Sequence | 566 | L | B | **0.97** | 0.001 | 0.03 | 0.449 |
| Sequence | 567 | K | E | **0.97** | 0.001 | 0.03 | 0.458444 |
| Sequence | 568 | K | E | **0.97** | 0.001 | 0.03 | 0.480778 |
| Sequence | 569 | V | B | **0.923** | 0.002 | 0.076 | 0.474111 |
| Sequence | 570 | M | B | **0.858** | 0.002 | 0.139 | 0.475111 |
| Sequence | 571 | E | E | **0.694** | 0.003 | 0.303 | **0.497333** |
| Sequence | 572 | G | E | 0.406 | 0.004 | **0.59** | **0.491778** |
| Sequence | 573 | E | E | 0.053 | 0.005 | **0.942** | 0.489778 |
| Sequence | 574 | D | E | 0.016 | 0.005 | **0.979** | 0.489111 |
| Sequence | 575 | S | E | **0.923** | 0.002 | 0.076 | **0.521778** |
| Sequence | 576 | A | E | **0.97** | 0.001 | 0.03 | **0.516** |
| Sequence | 577 | A | E | **0.975** | 0.003 | 0.022 | **0.523** |
| Sequence | 578 | I | B | **0.97** | 0.001 | 0.03 | **0.534667** |
| Sequence | 579 | K | E | **0.97** | 0.001 | 0.03 | **0.547** |
| Sequence | 580 | K | E | **0.97** | 0.001 | 0.03 | **0.555111** |
| Sequence | 581 | A | B | **0.97** | 0.001 | 0.03 | **0.540111** |
| Sequence | 582 | T | B | **0.97** | 0.001 | 0.03 | **0.544222** |
| Sequence | 583 | D | E | **0.97** | 0.001 | 0.03 | **0.558556** |
| Sequence | 584 | E | E | **0.97** | 0.001 | 0.03 | **0.542556** |
| Sequence | 585 | L | B | **0.988** | 0 | 0.012 | **0.527** |
| Sequence | 586 | A | E | **0.97** | 0.001 | 0.03 | **0.523333** |
| Sequence | 587 | S | E | **0.97** | 0.001 | 0.03 | **0.517111** |
| Sequence | 588 | A | B | **0.97** | 0.001 | 0.03 | 0.488444 |
| Sequence | 589 | S | B | **0.97** | 0.001 | 0.03 | 0.474444 |
| Sequence | 590 | H | E | **0.97** | 0.001 | 0.03 | 0.49 |
| Sequence | 591 | K | E | **0.97** | 0.001 | 0.03 | 0.474889 |
| Sequence | 592 | L | B | **0.97** | 0.001 | 0.03 | 0.466 |
| Sequence | 593 | A | B | **0.97** | 0.001 | 0.03 | 0.472778 |
| Sequence | 594 | E | E | **0.97** | 0.001 | 0.03 | 0.495222 |
| Sequence | 595 | Q | E | **0.97** | 0.001 | 0.03 | 0.489667 |
| Sequence | 596 | L | B | **0.97** | 0.001 | 0.03 | 0.482222 |
| Sequence | 597 | Y | E | **0.97** | 0.001 | 0.03 | **0.505556** |
| Sequence | 598 | K | E | **0.923** | 0.002 | 0.076 | **0.518** |
| Sequence | 599 | Q | E | **0.858** | 0.002 | 0.139 | **0.520333** |
| Sequence | 600 | T | E | **0.782** | 0.003 | 0.216 | **0.545111** |
| Sequence | 601 | Q | E | **0.694** | 0.003 | 0.303 | **0.564667** |
| Sequence | 602 | E | E | **0.6** | 0.003 | 0.397 | **0.570556** |
| Sequence | 603 | T | E | **0.502** | 0.002 | **0.495** | **0.573333** |
| Sequence | 604 | S | E | 0.246 | 0.004 | **0.75** | **0.577889** |
| Sequence | 605 | G | E | 0.109 | 0.005 | **0.886** | **0.587222** |
| Sequence | 606 | A | E | 0.115 | 0.016 | **0.868** | **0.584778** |
| Sequence | 607 | S | E | 0.058 | 0.017 | **0.925** | **0.583333** |
| Sequence | 608 | G | E | 0.058 | 0.017 | **0.925** | **0.578111** |
| Sequence | 609 | D | E | 0.058 | 0.017 | **0.925** | **0.560333** |
| Sequence | 610 | P | E | 0.058 | 0.017 | **0.925** | **0.551111** |
| Sequence | 611 | T | E | 0.058 | 0.017 | **0.925** | **0.551667** |
| Sequence | 612 | D | E | 0.115 | 0.016 | **0.868** | **0.554333** |
| Sequence | 613 | T | E | 0.115 | 0.016 | **0.868** | **0.553889** |
| Sequence | 614 | S | E | 0.115 | 0.016 | **0.868** | **0.55** |
| Sequence | 615 | A | E | 0.115 | 0.016 | **0.868** | **0.557444** |
| Sequence | 616 | S | E | 0.115 | 0.016 | **0.868** | **0.556111** |
| Sequence | 617 | S | E | 0.058 | 0.017 | **0.925** | **0.552889** |
| Sequence | 618 | S | E | 0.058 | 0.017 | **0.925** | **0.559889** |
| Sequence | 619 | K | E | 0.058 | 0.017 | **0.925** | **0.564333** |
| Sequence | 620 | S | E | 0.053 | 0.005 | **0.942** | **0.555556** |
| Sequence | 621 | G | E | 0.016 | 0.005 | **0.979** | **0.545889** |
| Sequence | 622 | D | E | 0.018 | 0.019 | **0.964** | **0.548889** |
| Sequence | 623 | D | E | 0.018 | 0.088 | **0.893** | **0.540111** |
| Sequence | 624 | V | B | 0.004 | 0.42 | **0.576** | **0.528111** |
| Sequence | 625 | V | E | 0.004 | **0.616** | 0.381 | **0.513778** |
| Sequence | 626 | D | E | 0.004 | **0.616** | 0.381 | **0.509667** |
| Sequence | 627 | A | B | 0.004 | **0.514** | 0.481 | **0.496667** |
| Sequence | 628 | D | E | 0.003 | **0.718** | 0.279 | 0.486778 |
| Sequence | 629 | F | B | 0.002 | **0.816** | 0.182 | 0.483889 |
| Sequence | 630 | T | E | 0.001 | **0.9** | 0.099 | 0.426 |
| Sequence | 631 | E | E | 0.002 | **0.816** | 0.182 | 0.362556 |
| Sequence | 632 | V | E | 0.004 | **0.616** | 0.381 | 0.310111 |
| Sequence | 633 | K | E | 0.018 | 0.019 | **0.964** | 0.254778 |
| All predictions above 0.49 are in bold font for probabilityof helix/sheet, loop, and B-cell antigenicity respectively. | | | | | | | |

**S6 Table:** B-cell epitope prediction, probability of being a helix, loop, or sheet for **Q1MPM8** through BepiPred - 2.0.

| **Entry** | **Position** | **Resn** | **Exposed/Buried** | **Helix** | **Sheet** | **Coil** | **Epitope** |
| --- | --- | --- | --- | --- | --- | --- | --- |
| Sequence | 1 | M | E | 0.016 | 0.005 | **0.979** | 0.230667 |
| Sequence | 2 | K | E | **0.717** | 0.014 | 0.269 | 0.279222 |
| Sequence | 3 | R | E | **0.923** | 0.002 | 0.076 | 0.308444 |
| Sequence | 4 | L | E | **0.923** | 0.002 | 0.076 | 0.331444 |
| Sequence | 5 | L | B | **0.975** | 0.003 | 0.022 | 0.351889 |
| Sequence | 6 | L | B | **0.97** | 0.001 | 0.03 | 0.340778 |
| Sequence | 7 | C | B | **0.97** | 0.001 | 0.03 | 0.318 |
| Sequence | 8 | I | E | **0.97** | 0.001 | 0.03 | 0.3 |
| Sequence | 9 | I | E | **0.988** | 0 | 0.012 | 0.279333 |
| Sequence | 10 | T | B | **0.988** | 0 | 0.012 | 0.260667 |
| Sequence | 11 | C | B | **0.97** | 0.001 | 0.03 | 0.247444 |
| Sequence | 12 | V | B | **0.97** | 0.001 | 0.03 | 0.260556 |
| Sequence | 13 | I | B | **0.923** | 0.002 | 0.076 | 0.274667 |
| Sequence | 14 | V | B | **0.858** | 0.002 | 0.139 | 0.309222 |
| Sequence | 15 | S | B | **0.694** | 0.003 | 0.303 | 0.339111 |
| Sequence | 16 | S | B | 0.406 | 0.004 | **0.59** | 0.379 |
| Sequence | 17 | C | B | 0.246 | 0.004 | **0.75** | 0.417 |
| Sequence | 18 | S | B | 0.115 | 0.016 | **0.868** | 0.46 |
| Sequence | 19 | F | B | 0.018 | 0.019 | **0.964** | **0.490889** |
| Sequence | 20 | A | B | 0.018 | 0.019 | **0.964** | **0.527222** |
| Sequence | 21 | P | E | 0.018 | 0.019 | **0.964** | **0.548556** |
| Sequence | 22 | D | E | 0.018 | 0.019 | **0.964** | **0.576556** |
| Sequence | 23 | Y | B | 0.018 | 0.019 | **0.964** | **0.589** |
| Sequence | 24 | N | E | 0.018 | 0.019 | **0.964** | **0.59** |
| Sequence | 25 | R | E | 0.018 | 0.019 | **0.964** | **0.589111** |
| Sequence | 26 | P | E | 0.018 | 0.019 | **0.964** | **0.570111** |
| Sequence | 27 | H | E | 0.018 | 0.047 | **0.935** | **0.545** |
| Sequence | 28 | L | E | 0.053 | 0.043 | **0.903** | **0.546889** |
| Sequence | 29 | E | E | 0.113 | 0.043 | **0.844** | **0.527222** |
| Sequence | 30 | L | E | 0.181 | 0.016 | **0.803** | **0.513** |
| Sequence | 31 | P | E | 0.181 | 0.016 | **0.803** | **0.511** |
| Sequence | 32 | E | E | 0.257 | 0.016 | **0.727** | **0.504222** |
| Sequence | 33 | V | E | 0.257 | 0.016 | **0.727** | **0.508556** |
| Sequence | 34 | W | B | 0.257 | 0.016 | **0.727** | **0.518111** |
| Sequence | 35 | V | E | 0.184 | 0.043 | **0.773** | **0.535556** |
| Sequence | 36 | S | E | 0.115 | 0.016 | **0.868** | **0.555556** |
| Sequence | 37 | S | E | 0.115 | 0.016 | **0.868** | **0.559444** |
| Sequence | 38 | P | E | 0.058 | 0.017 | **0.925** | **0.567222** |
| Sequence | 39 | E | E | 0.058 | 0.017 | **0.925** | **0.584** |
| Sequence | 40 | T | E | 0.058 | 0.017 | **0.925** | **0.583** |
| Sequence | 41 | G | E | 0.058 | 0.017 | **0.925** | **0.584556** |
| Sequence | 42 | V | E | 0.058 | 0.017 | **0.925** | **0.587222** |
| Sequence | 43 | P | E | 0.115 | 0.016 | **0.868** | **0.582** |
| Sequence | 44 | A | E | 0.181 | 0.016 | **0.803** | **0.571444** |
| Sequence | 45 | S | E | 0.339 | 0.016 | **0.645** | **0.565333** |
| Sequence | 46 | M | E | **0.694** | 0.003 | 0.303 | **0.559556** |
| Sequence | 47 | Q | E | **0.782** | 0.003 | 0.216 | **0.567111** |
| Sequence | 48 | W | B | **0.858** | 0.002 | 0.139 | **0.553** |
| Sequence | 49 | W | B | **0.879** | 0.01 | 0.111 | **0.554778** |
| Sequence | 50 | K | E | **0.782** | 0.003 | 0.216 | **0.546667** |
| Sequence | 51 | R | E | **0.6** | 0.003 | 0.397 | **0.550667** |
| Sequence | 52 | F | B | 0.246 | 0.004 | **0.75** | **0.547556** |
| Sequence | 53 | N | E | 0.016 | 0.005 | **0.979** | **0.533** |
| Sequence | 54 | D | B | 0.246 | 0.004 | **0.75** | **0.535778** |
| Sequence | 55 | S | E | **0.858** | 0.002 | 0.139 | **0.530556** |
| Sequence | 56 | T | E | **0.858** | 0.002 | 0.139 | **0.490444** |
| Sequence | 57 | L | B | **0.97** | 0.001 | 0.03 | 0.475556 |
| Sequence | 58 | D | E | **0.97** | 0.001 | 0.03 | 0.464111 |
| Sequence | 59 | I | E | **0.97** | 0.001 | 0.03 | 0.451111 |
| Sequence | 60 | L | B | **0.97** | 0.001 | 0.03 | 0.423222 |
| Sequence | 61 | V | B | **0.988** | 0 | 0.012 | 0.422222 |
| Sequence | 62 | A | E | **0.97** | 0.001 | 0.03 | 0.431889 |
| Sequence | 63 | E | E | **0.97** | 0.001 | 0.03 | 0.426556 |
| Sequence | 64 | A | B | **0.975** | 0.003 | 0.022 | 0.424778 |
| Sequence | 65 | L | B | **0.938** | 0.007 | 0.055 | 0.461667 |
| Sequence | 66 | Q | E | **0.694** | 0.003 | 0.303 | 0.484333 |
| Sequence | 67 | H | E | 0.246 | 0.004 | **0.75** | 0.476333 |
| Sequence | 68 | N | B | 0.003 | 0.003 | **0.994** | **0.497889** |
| Sequence | 69 | R | E | **0.6** | 0.003 | 0.397 | **0.517222** |
| Sequence | 70 | D | E | **0.879** | 0.01 | 0.111 | **0.490444** |
| Sequence | 71 | L | B | **0.975** | 0.003 | 0.022 | 0.489333 |
| Sequence | 72 | I | E | **0.975** | 0.003 | 0.022 | 0.483889 |
| Sequence | 73 | A | E | **0.97** | 0.001 | 0.03 | 0.485556 |
| Sequence | 74 | A | B | **0.97** | 0.001 | 0.03 | 0.459667 |
| Sequence | 75 | V | E | **0.97** | 0.001 | 0.03 | 0.465444 |
| Sequence | 76 | A | E | **0.97** | 0.001 | 0.03 | 0.478778 |
| Sequence | 77 | R | E | **0.97** | 0.001 | 0.03 | 0.458667 |
| Sequence | 78 | V | B | **0.97** | 0.001 | 0.03 | 0.465 |
| Sequence | 79 | D | E | **0.97** | 0.001 | 0.03 | 0.486 |
| Sequence | 80 | Y | E | **0.97** | 0.001 | 0.03 | **0.494111** |
| Sequence | 81 | A | B | **0.97** | 0.001 | 0.03 | **0.496778** |
| Sequence | 82 | Q | E | **0.97** | 0.001 | 0.03 | **0.502333** |
| Sequence | 83 | A | E | **0.97** | 0.001 | 0.03 | **0.515111** |
| Sequence | 84 | Q | E | **0.97** | 0.001 | 0.03 | **0.507556** |
| Sequence | 85 | L | B | **0.923** | 0.002 | 0.076 | **0.517** |
| Sequence | 86 | G | E | **0.923** | 0.002 | 0.076 | **0.539111** |
| Sequence | 87 | V | E | **0.923** | 0.002 | 0.076 | **0.547111** |
| Sequence | 88 | A | B | **0.879** | 0.01 | 0.111 | **0.556444** |
| Sequence | 89 | R | E | **0.717** | 0.014 | 0.269 | **0.560889** |
| Sequence | 90 | S | B | 0.43 | 0.016 | **0.555** | **0.566556** |
| Sequence | 91 | D | E | 0.181 | 0.016 | **0.803** | **0.567778** |
| Sequence | 92 | L | B | 0.053 | 0.043 | **0.903** | **0.576667** |
| Sequence | 93 | F | B | 0.018 | 0.088 | **0.893** | **0.579556** |
| Sequence | 94 | P | B | 0.004 | 0.138 | **0.858** | **0.567333** |
| Sequence | 95 | H | B | 0.004 | **0.616** | 0.381 | **0.559778** |
| Sequence | 96 | F | E | 0.002 | **0.816** | 0.182 | **0.547** |
| Sequence | 97 | S | B | 0.002 | **0.816** | 0.182 | **0.545889** |
| Sequence | 98 | G | E | 0.002 | **0.816** | 0.182 | **0.543889** |
| Sequence | 99 | N | B | 0.002 | **0.816** | 0.182 | **0.538222** |
| Sequence | 100 | A | E | 0.004 | **0.616** | 0.381 | **0.538444** |
| Sequence | 101 | Q | B | 0.021 | 0.451 | **0.528** | **0.545111** |
| Sequence | 102 | A | E | 0.021 | 0.451 | **0.528** | **0.549111** |
| Sequence | 103 | T | B | 0.022 | **0.552** | 0.426 | **0.556444** |
| Sequence | 104 | P | E | 0.021 | 0.451 | **0.528** | **0.569111** |
| Sequence | 105 | V | E | 0.021 | 0.451 | **0.528** | **0.583** |
| Sequence | 106 | W | E | 0.022 | 0.359 | **0.619** | **0.602111** |
| Sequence | 107 | V | E | 0.02 | 0.205 | **0.775** | **0.622222** |
| Sequence | 108 | D | E | 0.018 | 0.088 | **0.893** | **0.641889** |
| Sequence | 109 | H | E | 0.018 | 0.047 | **0.935** | **0.650778** |
| Sequence | 110 | K | E | 0.018 | 0.047 | **0.935** | **0.661667** |
| Sequence | 111 | R | E | 0.053 | 0.043 | **0.903** | **0.664333** |
| Sequence | 112 | V | E | 0.018 | 0.047 | **0.935** | **0.684222** |
| Sequence | 113 | T | E | 0.018 | 0.047 | **0.935** | **0.680111** |
| Sequence | 114 | D | E | 0.018 | 0.047 | **0.935** | **0.674111** |
| Sequence | 115 | G | E | 0.018 | 0.047 | **0.935** | **0.648333** |
| Sequence | 116 | Q | E | 0.019 | 0.141 | **0.84** | **0.630444** |
| Sequence | 117 | S | E | 0.004 | 0.197 | **0.799** | **0.599667** |
| Sequence | 118 | P | B | 0.004 | 0.42 | **0.576** | **0.585111** |
| Sequence | 119 | Y | E | 0.004 | **0.616** | 0.381 | **0.563111** |
| Sequence | 120 | S | B | 0.002 | **0.816** | 0.182 | **0.552889** |
| Sequence | 121 | A | E | 0.001 | **0.9** | 0.099 | **0.524889** |
| Sequence | 122 | N | B | 0.001 | **0.9** | 0.099 | **0.509667** |
| Sequence | 123 | F | E | 0.001 | **0.9** | 0.099 | **0.502667** |
| Sequence | 124 | S | B | 0.001 | **0.9** | 0.099 | **0.498778** |
| Sequence | 125 | A | E | 0.001 | **0.9** | 0.099 | 0.486111 |
| Sequence | 126 | S | B | 0.011 | **0.918** | 0.071 | **0.499333** |
| Sequence | 127 | W | B | 0.018 | **0.846** | 0.136 | **0.497667** |
| Sequence | 128 | E | B | 0.018 | **0.846** | 0.136 | **0.507222** |
| Sequence | 129 | I | B | 0.087 | **0.683** | 0.23 | **0.506889** |
| Sequence | 130 | D | B | 0.231 | 0.33 | 0.439 | **0.515** |
| Sequence | 131 | I | B | 0.386 | 0.097 | **0.517** | **0.525889** |
| Sequence | 132 | W | B | 0.43 | 0.016 | **0.555** | **0.532444** |
| Sequence | 133 | G | E | **0.522** | 0.016 | 0.462 | **0.530667** |
| Sequence | 134 | K | E | **0.717** | 0.014 | 0.269 | **0.538889** |
| Sequence | 135 | I | B | **0.802** | 0.014 | 0.185 | **0.537333** |
| Sequence | 136 | R | E | **0.858** | 0.002 | 0.139 | **0.537111** |
| Sequence | 137 | N | E | **0.923** | 0.002 | 0.076 | **0.522778** |
| Sequence | 138 | A | B | **0.97** | 0.001 | 0.03 | **0.515444** |
| Sequence | 139 | K | B | **0.97** | 0.001 | 0.03 | **0.512556** |
| Sequence | 140 | D | E | **0.97** | 0.001 | 0.03 | **0.499** |
| Sequence | 141 | A | B | **0.97** | 0.001 | 0.03 | 0.487889 |
| Sequence | 142 | A | B | **0.923** | 0.002 | 0.076 | 0.488778 |
| Sequence | 143 | F | E | **0.923** | 0.002 | 0.076 | **0.493556** |
| Sequence | 144 | S | E | **0.923** | 0.002 | 0.076 | **0.490778** |
| Sequence | 145 | Q | E | **0.923** | 0.002 | 0.076 | **0.490111** |
| Sequence | 146 | L | B | **0.97** | 0.001 | 0.03 | **0.498** |
| Sequence | 147 | M | E | **0.97** | 0.001 | 0.03 | **0.502667** |
| Sequence | 148 | A | B | **0.97** | 0.001 | 0.03 | **0.512333** |
| Sequence | 149 | T | B | **0.923** | 0.002 | 0.076 | **0.520778** |
| Sequence | 150 | E | E | **0.97** | 0.001 | 0.03 | **0.538111** |
| Sequence | 151 | A | E | **0.97** | 0.001 | 0.03 | **0.539889** |
| Sequence | 152 | E | E | **0.97** | 0.001 | 0.03 | **0.516222** |
| Sequence | 153 | K | B | **0.97** | 0.001 | 0.03 | **0.515444** |
| Sequence | 154 | E | E | **0.97** | 0.001 | 0.03 | **0.501222** |
| Sequence | 155 | G | E | **0.97** | 0.001 | 0.03 | 0.476333 |
| Sequence | 156 | V | B | **0.97** | 0.001 | 0.03 | 0.446778 |
| Sequence | 157 | F | B | **0.97** | 0.001 | 0.03 | 0.400444 |
| Sequence | 158 | L | B | **0.97** | 0.001 | 0.03 | 0.377556 |
| Sequence | 159 | S | E | **0.988** | 0 | 0.012 | 0.352667 |
| Sequence | 160 | I | B | **0.988** | 0 | 0.012 | 0.340667 |
| Sequence | 161 | A | B | **0.988** | 0 | 0.012 | 0.336 |
| Sequence | 162 | A | B | **0.988** | 0 | 0.012 | 0.333667 |
| Sequence | 163 | Q | E | **0.988** | 0 | 0.012 | 0.342222 |
| Sequence | 164 | T | B | **0.988** | 0 | 0.012 | 0.345889 |
| Sequence | 165 | A | B | **0.988** | 0 | 0.012 | 0.367667 |
| Sequence | 166 | N | E | **0.97** | 0.001 | 0.03 | 0.387667 |
| Sequence | 167 | A | B | **0.97** | 0.001 | 0.03 | 0.385889 |
| Sequence | 168 | Y | B | **0.988** | 0 | 0.012 | 0.386889 |
| Sequence | 169 | F | B | **0.97** | 0.001 | 0.03 | 0.39 |
| Sequence | 170 | L | E | **0.97** | 0.001 | 0.03 | 0.392 |
| Sequence | 171 | L | B | **0.97** | 0.001 | 0.03 | 0.396667 |
| Sequence | 172 | R | B | **0.97** | 0.001 | 0.03 | 0.403222 |
| Sequence | 173 | S | B | **0.97** | 0.001 | 0.03 | 0.415667 |
| Sequence | 174 | L | B | **0.97** | 0.001 | 0.03 | 0.411444 |
| Sequence | 175 | D | E | **0.97** | 0.001 | 0.03 | 0.424667 |
| Sequence | 176 | L | E | **0.97** | 0.001 | 0.03 | 0.445667 |
| Sequence | 177 | Q | B | **0.97** | 0.001 | 0.03 | 0.428667 |
| Sequence | 178 | C | B | **0.97** | 0.001 | 0.03 | 0.445111 |
| Sequence | 179 | S | E | **0.97** | 0.001 | 0.03 | 0.465889 |
| Sequence | 180 | I | B | **0.97** | 0.001 | 0.03 | 0.463111 |
| Sequence | 181 | A | B | **0.97** | 0.001 | 0.03 | 0.468333 |
| Sequence | 182 | E | E | **0.97** | 0.001 | 0.03 | 0.483556 |
| Sequence | 183 | R | E | **0.97** | 0.001 | 0.03 | **0.505444** |
| Sequence | 184 | T | B | **0.97** | 0.001 | 0.03 | **0.514222** |
| Sequence | 185 | V | B | **0.97** | 0.001 | 0.03 | **0.526444** |
| Sequence | 186 | K | E | **0.97** | 0.001 | 0.03 | **0.545778** |
| Sequence | 187 | T | E | **0.97** | 0.001 | 0.03 | **0.543111** |
| Sequence | 188 | R | B | **0.97** | 0.001 | 0.03 | **0.544667** |
| Sequence | 189 | E | E | **0.97** | 0.001 | 0.03 | **0.534778** |
| Sequence | 190 | D | E | **0.97** | 0.001 | 0.03 | **0.536444** |
| Sequence | 191 | A | B | **0.97** | 0.001 | 0.03 | **0.513222** |
| Sequence | 192 | L | B | **0.97** | 0.001 | 0.03 | **0.504778** |
| Sequence | 193 | S | E | **0.97** | 0.001 | 0.03 | 0.486444 |
| Sequence | 194 | I | B | **0.97** | 0.001 | 0.03 | 0.477 |
| Sequence | 195 | Y | B | **0.97** | 0.001 | 0.03 | 0.484889 |
| Sequence | 196 | T | E | **0.988** | 0 | 0.012 | **0.492444** |
| Sequence | 197 | A | E | **0.988** | 0 | 0.012 | **0.498889** |
| Sequence | 198 | Q | B | **0.97** | 0.001 | 0.03 | **0.517** |
| Sequence | 199 | Y | B | **0.923** | 0.002 | 0.076 | **0.517** |
| Sequence | 200 | Q | E | **0.858** | 0.002 | 0.139 | **0.535** |
| Sequence | 201 | K | E | **0.502** | 0.002 | **0.495** | **0.545556** |
| Sequence | 202 | G | E | 0.176 | 0.004 | **0.82** | **0.565556** |
| Sequence | 203 | F | E | 0.109 | 0.005 | **0.886** | **0.576667** |
| Sequence | 204 | I | B | 0.181 | 0.016 | **0.803** | **0.586667** |
| Sequence | 205 | N | E | 0.115 | 0.016 | **0.868** | **0.576778** |
| Sequence | 206 | K | E | **0.879** | 0.01 | 0.111 | **0.574** |
| Sequence | 207 | L | E | **0.879** | 0.01 | 0.111 | **0.572667** |
| Sequence | 208 | D | E | **0.938** | 0.007 | 0.055 | **0.557778** |
| Sequence | 209 | L | B | **0.97** | 0.001 | 0.03 | **0.553333** |
| Sequence | 210 | T | E | **0.97** | 0.001 | 0.03 | **0.540889** |
| Sequence | 211 | R | E | **0.97** | 0.001 | 0.03 | **0.525** |
| Sequence | 212 | A | B | **0.97** | 0.001 | 0.03 | **0.505222** |
| Sequence | 213 | K | E | **0.97** | 0.001 | 0.03 | **0.504222** |
| Sequence | 214 | T | E | **0.97** | 0.001 | 0.03 | **0.503111** |
| Sequence | 215 | E | E | **0.97** | 0.001 | 0.03 | 0.486333 |
| Sequence | 216 | V | B | **0.97** | 0.001 | 0.03 | 0.485 |
| Sequence | 217 | E | E | **0.97** | 0.001 | 0.03 | **0.499333** |
| Sequence | 218 | T | E | **0.97** | 0.001 | 0.03 | **0.501889** |
| Sequence | 219 | A | B | **0.97** | 0.001 | 0.03 | **0.509** |
| Sequence | 220 | R | E | **0.97** | 0.001 | 0.03 | **0.524** |
| Sequence | 221 | T | E | **0.923** | 0.002 | 0.076 | **0.537889** |
| Sequence | 222 | A | E | **0.782** | 0.003 | 0.216 | **0.525333** |
| Sequence | 223 | L | B | **0.923** | 0.002 | 0.076 | **0.537778** |
| Sequence | 224 | Y | E | **0.97** | 0.001 | 0.03 | **0.558556** |
| Sequence | 225 | Q | E | **0.97** | 0.001 | 0.03 | **0.554444** |
| Sequence | 226 | K | B | **0.97** | 0.001 | 0.03 | **0.549778** |
| Sequence | 227 | R | E | **0.97** | 0.001 | 0.03 | **0.558** |
| Sequence | 228 | I | E | **0.97** | 0.001 | 0.03 | **0.56** |
| Sequence | 229 | A | E | **0.97** | 0.001 | 0.03 | **0.543667** |
| Sequence | 230 | Q | B | **0.97** | 0.001 | 0.03 | **0.538** |
| Sequence | 231 | E | E | **0.97** | 0.001 | 0.03 | **0.539667** |
| Sequence | 232 | N | E | **0.97** | 0.001 | 0.03 | **0.526333** |
| Sequence | 233 | A | B | **0.97** | 0.001 | 0.03 | 0.483889 |
| Sequence | 234 | E | E | **0.97** | 0.001 | 0.03 | 0.480333 |
| Sequence | 235 | T | E | **0.97** | 0.001 | 0.03 | 0.476667 |
| Sequence | 236 | A | B | **0.988** | 0 | 0.012 | 0.444556 |
| Sequence | 237 | L | B | **0.988** | 0 | 0.012 | 0.416 |
| Sequence | 238 | S | B | **0.97** | 0.001 | 0.03 | 0.431778 |
| Sequence | 239 | V | E | **0.975** | 0.003 | 0.022 | 0.440556 |
| Sequence | 240 | L | B | **0.938** | 0.007 | 0.055 | 0.447111 |
| Sequence | 241 | L | B | **0.717** | 0.014 | 0.269 | 0.449778 |
| Sequence | 242 | G | E | 0.109 | 0.005 | **0.886** | **0.498** |
| Sequence | 243 | R | E | 0.016 | 0.005 | **0.979** | **0.511444** |
| Sequence | 244 | S | E | 0.018 | 0.019 | **0.964** | **0.519** |
| Sequence | 245 | P | B | 0.058 | 0.017 | **0.925** | **0.552444** |
| Sequence | 246 | R | E | 0.058 | 0.017 | **0.925** | **0.591** |
| Sequence | 247 | L | E | 0.181 | 0.016 | **0.803** | **0.585444** |
| Sequence | 248 | I | B | 0.257 | 0.016 | **0.727** | **0.590222** |
| Sequence | 249 | M | E | 0.257 | 0.016 | **0.727** | **0.603444** |
| Sequence | 250 | D | E | 0.257 | 0.016 | **0.727** | **0.617889** |
| Sequence | 251 | T | E | 0.268 | 0.043 | **0.689** | **0.623111** |
| Sequence | 252 | A | E | 0.268 | 0.043 | **0.689** | **0.632** |
| Sequence | 253 | I | E | 0.268 | 0.043 | **0.689** | **0.644222** |
| Sequence | 254 | E | E | 0.268 | 0.043 | **0.689** | **0.647889** |
| Sequence | 255 | R | E | 0.181 | 0.016 | **0.803** | **0.637556** |
| Sequence | 256 | G | E | 0.115 | 0.016 | **0.868** | **0.653889** |
| Sequence | 257 | V | E | 0.113 | 0.043 | **0.844** | **0.652556** |
| Sequence | 258 | S | E | 0.115 | 0.016 | **0.868** | **0.630333** |
| Sequence | 259 | M | E | 0.115 | 0.016 | **0.868** | **0.632778** |
| Sequence | 260 | K | E | 0.115 | 0.016 | **0.868** | **0.606889** |
| Sequence | 261 | D | E | 0.115 | 0.016 | **0.868** | **0.585333** |
| Sequence | 262 | L | E | 0.181 | 0.016 | **0.803** | **0.579333** |
| Sequence | 263 | S | E | 0.181 | 0.016 | **0.803** | **0.569778** |
| Sequence | 264 | C | E | 0.115 | 0.016 | **0.868** | **0.568111** |
| Sequence | 265 | I | B | 0.115 | 0.016 | **0.868** | **0.552333** |
| Sequence | 266 | P | E | 0.181 | 0.016 | **0.803** | **0.554444** |
| Sequence | 267 | V | E | 0.181 | 0.016 | **0.803** | **0.558667** |
| Sequence | 268 | I | B | 0.181 | 0.016 | **0.803** | **0.537667** |
| Sequence | 269 | P | E | 0.181 | 0.016 | **0.803** | **0.542444** |
| Sequence | 270 | Q | E | 0.339 | 0.016 | **0.645** | **0.549667** |
| Sequence | 271 | G | E | 0.257 | 0.016 | **0.727** | **0.544333** |
| Sequence | 272 | I | B | 0.339 | 0.016 | **0.645** | **0.526222** |
| Sequence | 273 | P | B | **0.879** | 0.01 | 0.111 | **0.523111** |
| Sequence | 274 | S | E | **0.923** | 0.002 | 0.076 | **0.524667** |
| Sequence | 275 | E | E | **0.97** | 0.001 | 0.03 | **0.517667** |
| Sequence | 276 | L | B | **0.923** | 0.002 | 0.076 | **0.522889** |
| Sequence | 277 | L | B | **0.858** | 0.002 | 0.139 | **0.530222** |
| Sequence | 278 | E | E | **0.694** | 0.003 | 0.303 | **0.538667** |
| Sequence | 279 | R | E | 0.321 | 0.003 | **0.675** | **0.521556** |
| Sequence | 280 | R | B | 0.053 | 0.005 | **0.942** | **0.524556** |
| Sequence | 281 | P | E | **0.694** | 0.003 | 0.303 | **0.538333** |
| Sequence | 282 | D | E | **0.802** | 0.014 | 0.185 | **0.531** |
| Sequence | 283 | I | B | **0.97** | 0.001 | 0.03 | **0.534111** |
| Sequence | 284 | R | E | **0.975** | 0.003 | 0.022 | **0.535111** |
| Sequence | 285 | Q | E | **0.97** | 0.001 | 0.03 | **0.528556** |
| Sequence | 286 | A | B | **0.97** | 0.001 | 0.03 | **0.514889** |
| Sequence | 287 | E | E | **0.97** | 0.001 | 0.03 | **0.506222** |
| Sequence | 288 | Y | E | **0.97** | 0.001 | 0.03 | **0.515778** |
| Sequence | 289 | T | E | **0.97** | 0.001 | 0.03 | **0.506889** |
| Sequence | 290 | L | B | **0.97** | 0.001 | 0.03 | **0.507778** |
| Sequence | 291 | K | E | **0.97** | 0.001 | 0.03 | **0.514333** |
| Sequence | 292 | A | B | **0.97** | 0.001 | 0.03 | **0.506111** |
| Sequence | 293 | T | B | **0.97** | 0.001 | 0.03 | **0.502444** |
| Sequence | 294 | S | E | **0.923** | 0.002 | 0.076 | **0.507222** |
| Sequence | 295 | A | E | **0.858** | 0.002 | 0.139 | **0.507** |
| Sequence | 296 | N | E | **0.858** | 0.002 | 0.139 | **0.502556** |
| Sequence | 297 | I | B | **0.923** | 0.002 | 0.076 | **0.522444** |
| Sequence | 298 | G | E | **0.923** | 0.002 | 0.076 | **0.522556** |
| Sequence | 299 | V | E | **0.938** | 0.007 | 0.055 | **0.524778** |
| Sequence | 300 | A | B | **0.938** | 0.007 | 0.055 | **0.529778** |
| Sequence | 301 | R | E | **0.879** | 0.01 | 0.111 | **0.532667** |
| Sequence | 302 | A | E | **0.717** | 0.014 | 0.269 | **0.532444** |
| Sequence | 303 | A | E | 0.257 | 0.016 | **0.727** | **0.532** |
| Sequence | 304 | W | E | 0.115 | 0.016 | **0.868** | **0.533889** |
| Sequence | 305 | L | B | 0.018 | 0.047 | **0.935** | **0.534889** |
| Sequence | 306 | P | B | 0.018 | 0.088 | **0.893** | **0.509222** |
| Sequence | 307 | S | E | 0.005 | 0.336 | **0.66** | **0.505778** |
| Sequence | 308 | I | B | 0.003 | **0.718** | 0.279 | **0.503111** |
| Sequence | 309 | S | B | 0.002 | **0.816** | 0.182 | **0.496667** |
| Sequence | 310 | L | B | 0.001 | **0.9** | 0.099 | **0.504778** |
| Sequence | 311 | T | B | 0.021 | **0.756** | 0.223 | **0.507556** |
| Sequence | 312 | G | E | 0.023 | **0.655** | 0.322 | **0.504333** |
| Sequence | 313 | L | B | 0.021 | 0.451 | **0.528** | **0.525556** |
| Sequence | 314 | F | E | 0.021 | 0.451 | **0.528** | **0.535** |
| Sequence | 315 | G | B | 0.022 | 0.359 | **0.619** | **0.565333** |
| Sequence | 316 | I | E | 0.064 | 0.216 | **0.721** | **0.588222** |
| Sequence | 317 | V | E | 0.056 | 0.142 | **0.802** | **0.587444** |
| Sequence | 318 | S | E | 0.056 | 0.142 | **0.802** | **0.601333** |
| Sequence | 319 | P | E | 0.052 | 0.084 | **0.864** | **0.602444** |
| Sequence | 320 | H | E | 0.113 | 0.087 | **0.8** | **0.597222** |
| Sequence | 321 | L | B | 0.113 | 0.043 | **0.844** | **0.609444** |
| Sequence | 322 | S | E | 0.115 | 0.016 | **0.868** | **0.603889** |
| Sequence | 323 | D | E | 0.113 | 0.043 | **0.844** | **0.612556** |
| Sequence | 324 | L | B | 0.113 | 0.043 | **0.844** | **0.606333** |
| Sequence | 325 | L | B | 0.113 | 0.087 | **0.8** | **0.590889** |
| Sequence | 326 | K | E | 0.052 | 0.084 | **0.864** | **0.593222** |
| Sequence | 327 | N | E | 0.053 | 0.043 | **0.903** | **0.575111** |
| Sequence | 328 | P | E | 0.058 | 0.017 | **0.925** | **0.558222** |
| Sequence | 329 | L | B | 0.053 | 0.043 | **0.903** | **0.549333** |
| Sequence | 330 | K | E | 0.125 | 0.227 | **0.648** | **0.531778** |
| Sequence | 331 | T | B | 0.074 | 0.484 | 0.442 | **0.512222** |
| Sequence | 332 | W | E | 0.087 | **0.683** | 0.23 | **0.498778** |
| Sequence | 333 | S | B | 0.085 | **0.775** | 0.14 | 0.475111 |
| Sequence | 334 | Y | E | 0.085 | **0.775** | 0.14 | 0.459333 |
| Sequence | 335 | G | B | 0.085 | **0.775** | 0.14 | 0.450333 |
| Sequence | 336 | E | E | 0.087 | **0.683** | 0.23 | 0.448333 |
| Sequence | 337 | T | B | 0.085 | **0.775** | 0.14 | 0.453333 |
| Sequence | 338 | G | E | 0.085 | **0.775** | 0.14 | 0.456333 |
| Sequence | 339 | T | B | 0.087 | **0.683** | 0.23 | 0.463111 |
| Sequence | 340 | V | B | 0.169 | **0.612** | 0.219 | 0.479889 |
| Sequence | 341 | P | B | 0.231 | 0.33 | 0.439 | **0.491** |
| Sequence | 342 | I | B | **0.502** | 0.102 | 0.396 | **0.509667** |
| Sequence | 343 | L | B | 0.455 | 0.046 | **0.498** | **0.529** |
| Sequence | 344 | D | E | 0.43 | 0.016 | **0.555** | **0.543111** |
| Sequence | 345 | F | E | 0.406 | 0.004 | **0.59** | **0.564556** |
| Sequence | 346 | G | E | 0.406 | 0.004 | **0.59** | **0.565333** |
| Sequence | 347 | Q | E | **0.502** | 0.002 | **0.495** | **0.569444** |
| Sequence | 348 | V | E | **0.782** | 0.003 | 0.216 | **0.566778** |
| Sequence | 349 | Y | E | **0.923** | 0.002 | 0.076 | **0.572222** |
| Sequence | 350 | Y | B | **0.97** | 0.001 | 0.03 | **0.558222** |
| Sequence | 351 | N | E | **0.97** | 0.001 | 0.03 | **0.550222** |
| Sequence | 352 | V | B | **0.97** | 0.001 | 0.03 | **0.548111** |
| Sequence | 353 | E | E | **0.97** | 0.001 | 0.03 | **0.546333** |
| Sequence | 354 | A | E | **0.97** | 0.001 | 0.03 | **0.542889** |
| Sequence | 355 | A | B | **0.97** | 0.001 | 0.03 | **0.552111** |
| Sequence | 356 | Q | E | **0.97** | 0.001 | 0.03 | **0.570444** |
| Sequence | 357 | A | B | **0.97** | 0.001 | 0.03 | **0.577111** |
| Sequence | 358 | K | E | **0.97** | 0.001 | 0.03 | **0.568111** |
| Sequence | 359 | E | B | **0.97** | 0.001 | 0.03 | **0.572556** |
| Sequence | 360 | R | E | **0.97** | 0.001 | 0.03 | **0.570111** |
| Sequence | 361 | E | E | **0.97** | 0.001 | 0.03 | **0.566556** |
| Sequence | 362 | A | B | **0.97** | 0.001 | 0.03 | **0.552111** |
| Sequence | 363 | L | B | **0.97** | 0.001 | 0.03 | **0.552444** |
| Sequence | 364 | A | E | **0.97** | 0.001 | 0.03 | **0.551** |
| Sequence | 365 | N | E | **0.97** | 0.001 | 0.03 | **0.543667** |
| Sequence | 366 | Y | B | **0.97** | 0.001 | 0.03 | **0.536111** |
| Sequence | 367 | E | E | **0.97** | 0.001 | 0.03 | **0.538444** |
| Sequence | 368 | K | E | **0.988** | 0 | 0.012 | **0.527778** |
| Sequence | 369 | T | B | **0.988** | 0 | 0.012 | **0.51** |
| Sequence | 370 | V | B | **0.988** | 0 | 0.012 | **0.506444** |
| Sequence | 371 | Q | E | **0.988** | 0 | 0.012 | **0.519111** |
| Sequence | 372 | N | E | **0.988** | 0 | 0.012 | **0.500889** |
| Sequence | 373 | A | B | **0.988** | 0 | 0.012 | 0.468333 |
| Sequence | 374 | F | B | **0.988** | 0 | 0.012 | 0.468778 |
| Sequence | 375 | K | E | **0.97** | 0.001 | 0.03 | 0.47 |
| Sequence | 376 | D | E | **0.988** | 0 | 0.012 | 0.451556 |
| Sequence | 377 | I | B | **0.988** | 0 | 0.012 | 0.440889 |
| Sequence | 378 | H | E | **0.988** | 0 | 0.012 | 0.455 |
| Sequence | 379 | D | E | **0.97** | 0.001 | 0.03 | 0.457444 |
| Sequence | 380 | A | B | **0.97** | 0.001 | 0.03 | 0.449889 |
| Sequence | 381 | L | B | **0.97** | 0.001 | 0.03 | 0.463444 |
| Sequence | 382 | I | E | **0.97** | 0.001 | 0.03 | **0.493778** |
| Sequence | 383 | R | E | **0.97** | 0.001 | 0.03 | **0.490778** |
| Sequence | 384 | Q | B | **0.97** | 0.001 | 0.03 | **0.508222** |
| Sequence | 385 | Y | E | **0.97** | 0.001 | 0.03 | **0.524778** |
| Sequence | 386 | E | E | **0.923** | 0.002 | 0.076 | **0.551222** |
| Sequence | 387 | S | B | **0.97** | 0.001 | 0.03 | **0.554333** |
| Sequence | 388 | K | E | **0.97** | 0.001 | 0.03 | **0.558222** |
| Sequence | 389 | N | E | **0.97** | 0.001 | 0.03 | **0.574556** |
| Sequence | 390 | I | E | **0.97** | 0.001 | 0.03 | **0.563667** |
| Sequence | 391 | V | B | **0.97** | 0.001 | 0.03 | **0.563333** |
| Sequence | 392 | N | E | **0.97** | 0.001 | 0.03 | **0.573111** |
| Sequence | 393 | S | E | **0.97** | 0.001 | 0.03 | **0.563556** |
| Sequence | 394 | L | B | **0.97** | 0.001 | 0.03 | **0.566778** |
| Sequence | 395 | E | E | **0.97** | 0.001 | 0.03 | **0.568222** |
| Sequence | 396 | R | E | **0.97** | 0.001 | 0.03 | **0.571333** |
| Sequence | 397 | M | B | **0.97** | 0.001 | 0.03 | **0.546778** |
| Sequence | 398 | V | B | **0.97** | 0.001 | 0.03 | **0.549889** |
| Sequence | 399 | K | E | **0.97** | 0.001 | 0.03 | **0.559111** |
| Sequence | 400 | E | E | **0.97** | 0.001 | 0.03 | **0.532889** |
| Sequence | 401 | L | B | **0.97** | 0.001 | 0.03 | **0.511111** |
| Sequence | 402 | R | E | **0.97** | 0.001 | 0.03 | **0.506667** |
| Sequence | 403 | I | E | **0.97** | 0.001 | 0.03 | **0.493** |
| Sequence | 404 | A | B | **0.97** | 0.001 | 0.03 | 0.459556 |
| Sequence | 405 | V | B | **0.97** | 0.001 | 0.03 | 0.468 |
| Sequence | 406 | H | E | **0.97** | 0.001 | 0.03 | 0.487778 |
| Sequence | 407 | L | B | **0.97** | 0.001 | 0.03 | 0.471111 |
| Sequence | 408 | A | B | **0.97** | 0.001 | 0.03 | 0.461889 |
| Sequence | 409 | R | E | **0.97** | 0.001 | 0.03 | **0.495** |
| Sequence | 410 | T | E | **0.97** | 0.001 | 0.03 | **0.510111** |
| Sequence | 411 | L | B | **0.97** | 0.001 | 0.03 | **0.517333** |
| Sequence | 412 | Y | B | **0.923** | 0.002 | 0.076 | **0.534778** |
| Sequence | 413 | D | E | **0.858** | 0.002 | 0.139 | **0.567444** |
| Sequence | 414 | N | E | **0.6** | 0.003 | 0.397 | **0.570111** |
| Sequence | 415 | G | E | 0.321 | 0.003 | **0.675** | **0.571444** |
| Sequence | 416 | Y | B | 0.321 | 0.003 | **0.675** | **0.573222** |
| Sequence | 417 | T | E | **0.522** | 0.016 | 0.462 | **0.581889** |
| Sequence | 418 | S | E | **0.622** | 0.015 | 0.363 | **0.553** |
| Sequence | 419 | Y | B | **0.802** | 0.014 | 0.185 | **0.536778** |
| Sequence | 420 | L | B | **0.938** | 0.007 | 0.055 | **0.531667** |
| Sequence | 421 | D | E | **0.938** | 0.007 | 0.055 | **0.516778** |
| Sequence | 422 | V | B | **0.97** | 0.001 | 0.03 | **0.503889** |
| Sequence | 423 | L | B | **0.97** | 0.001 | 0.03 | **0.498222** |
| Sequence | 424 | D | E | **0.97** | 0.001 | 0.03 | **0.495667** |
| Sequence | 425 | A | B | **0.97** | 0.001 | 0.03 | 0.488889 |
| Sequence | 426 | E | B | **0.97** | 0.001 | 0.03 | 0.483333 |
| Sequence | 427 | R | E | **0.97** | 0.001 | 0.03 | **0.506889** |
| Sequence | 428 | A | E | **0.97** | 0.001 | 0.03 | **0.508111** |
| Sequence | 429 | L | B | **0.97** | 0.001 | 0.03 | **0.512111** |
| Sequence | 430 | F | B | **0.97** | 0.001 | 0.03 | **0.522889** |
| Sequence | 431 | Q | E | **0.97** | 0.001 | 0.03 | **0.529222** |
| Sequence | 432 | S | B | **0.97** | 0.001 | 0.03 | **0.516111** |
| Sequence | 433 | E | B | **0.97** | 0.001 | 0.03 | **0.511889** |
| Sequence | 434 | L | E | **0.97** | 0.001 | 0.03 | **0.512889** |
| Sequence | 435 | D | E | **0.97** | 0.001 | 0.03 | **0.517778** |
| Sequence | 436 | L | B | **0.97** | 0.001 | 0.03 | **0.511222** |
| Sequence | 437 | A | B | **0.97** | 0.001 | 0.03 | **0.514889** |
| Sequence | 438 | S | E | **0.97** | 0.001 | 0.03 | **0.506889** |
| Sequence | 439 | A | B | **0.97** | 0.001 | 0.03 | **0.506778** |
| Sequence | 440 | W | B | **0.97** | 0.001 | 0.03 | **0.496** |
| Sequence | 441 | S | B | **0.97** | 0.001 | 0.03 | **0.492556** |
| Sequence | 442 | D | E | **0.97** | 0.001 | 0.03 | **0.491556** |
| Sequence | 443 | R | B | **0.97** | 0.001 | 0.03 | 0.476667 |
| Sequence | 444 | L | B | **0.97** | 0.001 | 0.03 | 0.439556 |
| Sequence | 445 | S | E | **0.97** | 0.001 | 0.03 | 0.429222 |
| Sequence | 446 | S | B | **0.97** | 0.001 | 0.03 | 0.397 |
| Sequence | 447 | I | B | **0.988** | 0 | 0.012 | 0.376111 |
| Sequence | 448 | V | B | **0.97** | 0.001 | 0.03 | 0.359222 |
| Sequence | 449 | Q | B | **0.988** | 0 | 0.012 | 0.356556 |
| Sequence | 450 | V | B | **0.988** | 0 | 0.012 | 0.352556 |
| Sequence | 451 | C | B | **0.988** | 0 | 0.012 | 0.365111 |
| Sequence | 452 | L | E | **0.97** | 0.001 | 0.03 | 0.385444 |
| Sequence | 453 | A | B | **0.923** | 0.002 | 0.076 | 0.420667 |
| Sequence | 454 | L | B | **0.782** | 0.003 | 0.216 | 0.432667 |
| Sequence | 455 | G | B | 0.406 | 0.004 | **0.59** | 0.471444 |
| Sequence | 456 | G | E | 0.109 | 0.005 | **0.886** | 0.434667 |
| Sequence | 457 | S | E | 0.058 | 0.017 | **0.925** | 0.389667 |
| Sequence | 458 | W | B | 0.058 | 0.017 | **0.925** | 0.342889 |
| Sequence | 459 | E | E | 0.003 | 0.003 | **0.994** | 0.300222 |
| All predictions above 0.49 are in bold font for probabilityof helix/sheet, loop, and B-cell antigenicity, respectively. | | | | | | | |

**S7 Table:** B-cell epitope prediction, probability of being a helix, loop, or sheet for Q1MQM4 through BepiPred - 2.0.

| **Entry** | **Position** | **Resn** | **Exposed/Buried** | **Helix** | **Sheet** | **Coil** | **Epitope** |
| --- | --- | --- | --- | --- | --- | --- | --- |
| Sequence | 1 | M | E | 0.016 | 0.005 | **0.979** | 0.2632222 |
| Sequence | 2 | A | E | 0.113 | 0.087 | **0.8** | 0.3262222 |
| Sequence | 3 | Y | B | 0.125 | 0.227 | **0.648** | 0.3958889 |
| Sequence | 4 | L | E | 0.135 | 0.317 | **0.548** | 0.458 |
| Sequence | 5 | S | E | 0.135 | 0.317 | **0.548** | **0.519** |
| Sequence | 6 | I | B | 0.231 | 0.33 | 0.439 | **0.5348889** |
| Sequence | 7 | S | E | 0.199 | 0.152 | **0.649** | **0.5285556** |
| Sequence | 8 | K | E | **0.561** | 0.047 | 0.393 | **0.5157778** |
| Sequence | 9 | N | E | **0.522** | 0.016 | 0.462 | **0.501** |
| Sequence | 10 | Q | B | **0.717** | 0.014 | 0.269 | 0.4697778 |
| Sequence | 11 | C | B | **0.802** | 0.014 | 0.185 | 0.43 |
| Sequence | 12 | K | B | **0.879** | 0.01 | 0.111 | 0.3827778 |
| Sequence | 13 | S | B | **0.938** | 0.007 | 0.055 | 0.3417778 |
| Sequence | 14 | F | B | **0.975** | 0.003 | 0.022 | 0.2972222 |
| Sequence | 15 | L | B | **0.975** | 0.003 | 0.022 | 0.2573333 |
| Sequence | 16 | I | B | **0.97** | 0.001 | 0.03 | 0.2381111 |
| Sequence | 17 | T | B | **0.988** | 0 | 0.012 | 0.2046667 |
| Sequence | 18 | L | B | **0.988** | 0 | 0.012 | 0.1796667 |
| Sequence | 19 | V | B | **0.97** | 0.001 | 0.03 | 0.1652222 |
| Sequence | 20 | T | B | **0.97** | 0.001 | 0.03 | 0.1704444 |
| Sequence | 21 | I | B | **0.975** | 0.003 | 0.022 | 0.1903333 |
| Sequence | 22 | F | B | **0.938** | 0.007 | 0.055 | 0.2185556 |
| Sequence | 23 | I | B | **0.879** | 0.01 | 0.111 | 0.2601111 |
| Sequence | 24 | M | B | **0.802** | 0.014 | 0.185 | 0.31 |
| Sequence | 25 | T | B | **0.622** | 0.015 | 0.363 | 0.358 |
| Sequence | 26 | S | E | 0.43 | 0.016 | **0.555** | 0.4032222 |
| Sequence | 27 | I | B | 0.43 | 0.016 | **0.555** | 0.4345556 |
| Sequence | 28 | P | E | 0.43 | 0.016 | **0.555** | 0.478 |
| Sequence | 29 | Q | E | 0.43 | 0.016 | **0.555** | 0.4865556 |
| Sequence | 30 | L | E | **0.561** | 0.047 | 0.393 | 0.4755556 |
| Sequence | 31 | A | B | **0.538** | 0.173 | 0.289 | 0.4775556 |
| Sequence | 32 | E | B | **0.578** | 0.229 | 0.194 | 0.4736667 |
| Sequence | 33 | A | B | **0.594** | 0.285 | 0.121 | 0.452 |
| Sequence | 34 | V | B | **0.594** | 0.285 | 0.121 | 0.4375556 |
| Sequence | 35 | E | B | **0.578** | 0.229 | 0.194 | 0.456 |
| Sequence | 36 | H | B | **0.578** | 0.229 | 0.194 | 0.4662222 |
| Sequence | 37 | F | B | 0.428 | 0.171 | 0.402 | 0.4704444 |
| Sequence | 38 | A | B | 0.278 | 0.093 | **0.628** | **0.4902222** |
| Sequence | 39 | N | E | 0.113 | 0.043 | **0.844** | **0.5153333** |
| Sequence | 40 | G | E | 0.018 | 0.047 | **0.935** | **0.5164444** |
| Sequence | 41 | V | E | 0.019 | 0.141 | **0.84** | **0.5024444** |
| Sequence | 42 | P | E | 0.022 | 0.359 | **0.619** | **0.5187778** |
| Sequence | 43 | T | E | 0.023 | **0.655** | 0.322 | **0.5235556** |
| Sequence | 44 | V | B | 0.018 | **0.846** | 0.136 | **0.4985556** |
| Sequence | 45 | V | B | 0.018 | **0.846** | 0.136 | **0.5172222** |
| Sequence | 46 | Q | E | 0.021 | **0.756** | 0.223 | **0.5233333** |
| Sequence | 47 | D | E | 0.022 | **0.552** | 0.426 | **0.5321111** |
| Sequence | 48 | V | B | 0.021 | 0.279 | **0.699** | **0.5313333** |
| Sequence | 49 | N | E | 0.019 | 0.141 | **0.84** | **0.5436667** |
| Sequence | 50 | V | E | 0.018 | 0.088 | **0.893** | **0.5623333** |
| Sequence | 51 | P | E | 0.053 | 0.043 | **0.903** | **0.5611111** |
| Sequence | 52 | A | B | 0.113 | 0.043 | **0.844** | **0.5506667** |
| Sequence | 53 | D | E | 0.191 | 0.086 | **0.723** | **0.5547778** |
| Sequence | 54 | S | B | 0.307 | 0.165 | **0.527** | **0.533** |
| Sequence | 55 | Y | B | 0.199 | 0.152 | **0.649** | **0.5172222** |
| Sequence | 56 | F | B | 0.199 | 0.152 | **0.649** | **0.5202222** |
| Sequence | 57 | G | B | 0.118 | 0.15 | **0.732** | **0.533** |
| Sequence | 58 | G | B | 0.052 | 0.084 | **0.864** | **0.5403333** |
| Sequence | 59 | A | B | 0.052 | 0.084 | **0.864** | **0.5526667** |
| Sequence | 60 | D | E | 0.052 | 0.084 | **0.864** | **0.5594444** |
| Sequence | 61 | S | E | 0.113 | 0.087 | **0.8** | **0.5752222** |
| Sequence | 62 | A | E | 0.113 | 0.087 | **0.8** | **0.5871111** |
| Sequence | 63 | V | E | 0.053 | 0.043 | **0.903** | **0.5981111** |
| Sequence | 64 | G | B | 0.053 | 0.043 | **0.903** | **0.6083333** |
| Sequence | 65 | P | E | 0.018 | 0.047 | **0.935** | **0.6118889** |
| Sequence | 66 | N | E | 0.018 | 0.047 | **0.935** | **0.6166667** |
| Sequence | 67 | P | E | 0.018 | 0.047 | **0.935** | **0.6094444** |
| Sequence | 68 | I | B | 0.018 | 0.047 | **0.935** | **0.6082222** |
| Sequence | 69 | A | E | 0.018 | 0.047 | **0.935** | **0.5964444** |
| Sequence | 70 | S | E | 0.018 | 0.047 | **0.935** | **0.5915556** |
| Sequence | 71 | T | E | 0.018 | 0.088 | **0.893** | **0.5605556** |
| Sequence | 72 | H | E | 0.019 | 0.141 | **0.84** | **0.5483333** |
| Sequence | 73 | L | B | 0.022 | 0.359 | **0.619** | **0.5292222** |
| Sequence | 74 | T | E | 0.023 | **0.655** | 0.322 | **0.523** |
| Sequence | 75 | I | B | 0.021 | **0.756** | 0.223 | **0.5282222** |
| Sequence | 76 | S | B | 0.087 | **0.683** | 0.23 | **0.5241111** |
| Sequence | 77 | T | B | 0.074 | 0.484 | 0.442 | **0.5148889** |
| Sequence | 78 | T | B | 0.135 | 0.317 | **0.548** | **0.529** |
| Sequence | 79 | Q | E | 0.199 | 0.152 | **0.649** | **0.5324444** |
| Sequence | 80 | G | E | 0.278 | 0.093 | **0.628** | **0.5556667** |
| Sequence | 81 | F | B | 0.278 | 0.093 | **0.628** | **0.5544444** |
| Sequence | 82 | G | B | 0.278 | 0.093 | **0.628** | **0.5456667** |
| Sequence | 83 | Q | E | 0.278 | 0.093 | **0.628** | **0.5281111** |
| Sequence | 84 | N | B | 0.216 | 0.235 | **0.548** | 0.4874444 |
| Sequence | 85 | A | B | 0.148 | 0.418 | 0.435 | 0.4522222 |
| Sequence | 86 | L | B | 0.172 | **0.69** | 0.138 | 0.4333333 |
| Sequence | 87 | E | B | 0.085 | **0.775** | 0.14 | 0.4107778 |
| Sequence | 88 | F | B | 0.085 | **0.775** | 0.14 | 0.3927778 |
| Sequence | 89 | V | B | 0.085 | **0.775** | 0.14 | 0.389 |
| Sequence | 90 | V | B | 0.087 | **0.683** | 0.23 | 0.3988889 |
| Sequence | 91 | G | B | 0.021 | 0.451 | **0.528** | 0.4297778 |
| Sequence | 92 | G | B | 0.02 | 0.205 | **0.775** | 0.458 |
| Sequence | 93 | S | B | 0.066 | 0.296 | **0.638** | 0.4844444 |
| Sequence | 94 | L | B | 0.066 | 0.296 | **0.638** | **0.5365556** |
| Sequence | 95 | A | B | 0.064 | 0.216 | **0.721** | **0.5675556** |
| Sequence | 96 | N | E | 0.064 | 0.216 | **0.721** | **0.6034444** |
| Sequence | 97 | G | B | 0.064 | 0.216 | **0.721** | **0.6277778** |
| Sequence | 98 | N | E | 0.018 | 0.088 | **0.893** | **0.6455556** |
| Sequence | 99 | G | E | 0.018 | 0.047 | **0.935** | **0.6536667** |
| Sequence | 100 | N | E | 0.018 | 0.047 | **0.935** | **0.648** |
| Sequence | 101 | P | E | 0.018 | 0.047 | **0.935** | **0.6387778** |
| Sequence | 102 | A | E | 0.019 | 0.141 | **0.84** | **0.6287778** |
| Sequence | 103 | N | E | 0.022 | 0.359 | **0.619** | **0.6146667** |
| Sequence | 104 | I | B | 0.004 | 0.42 | **0.576** | **0.5802222** |
| Sequence | 105 | N | E | 0.005 | 0.336 | **0.66** | **0.5442222** |
| Sequence | 106 | G | B | 0.004 | 0.197 | **0.799** | **0.4957778** |
| Sequence | 107 | D | E | 0.004 | 0.42 | **0.576** | 0.4495556 |
| Sequence | 108 | I | B | 0.001 | **0.9** | 0.099 | 0.3951111 |
| Sequence | 109 | V | B | 0.001 | **0.959** | 0.04 | 0.3826667 |
| Sequence | 110 | L | B | 0.001 | **0.959** | 0.04 | 0.3621111 |
| Sequence | 111 | I | B | 0.001 | **0.959** | 0.04 | 0.375 |
| Sequence | 112 | V | B | 0.001 | **0.959** | 0.04 | 0.3773333 |
| Sequence | 113 | E | B | 0.001 | **0.9** | 0.099 | 0.3978889 |
| Sequence | 114 | N | B | 0.022 | **0.552** | 0.426 | 0.4135556 |
| Sequence | 115 | T | B | 0.021 | 0.279 | **0.699** | 0.4461111 |
| Sequence | 116 | N | E | 0.066 | 0.296 | **0.638** | 0.4681111 |
| Sequence | 117 | T | B | 0.069 | 0.386 | **0.545** | 0.4805556 |
| Sequence | 118 | Q | B | 0.066 | 0.296 | **0.638** | 0.4637778 |
| Sequence | 119 | N | B | 0.069 | 0.386 | **0.545** | 0.4518889 |
| Sequence | 120 | S | B | 0.087 | **0.683** | 0.23 | 0.4245556 |
| Sequence | 121 | I | B | 0.085 | **0.775** | 0.14 | 0.4078889 |
| Sequence | 122 | I | B | 0.087 | **0.683** | 0.23 | 0.412 |
| Sequence | 123 | G | B | 0.022 | **0.552** | 0.426 | 0.412 |
| Sequence | 124 | G | B | 0.021 | 0.279 | **0.699** | 0.432 |
| Sequence | 125 | S | B | 0.021 | 0.279 | **0.699** | 0.4342222 |
| Sequence | 126 | M | B | 0.02 | 0.205 | **0.775** | 0.4612222 |
| Sequence | 127 | A | B | 0.019 | 0.141 | **0.84** | 0.4861111 |
| Sequence | 128 | N | E | 0.018 | 0.047 | **0.935** | 0.4897778 |
| Sequence | 129 | A | E | 0.018 | 0.047 | **0.935** | **0.4992222** |
| Sequence | 130 | A | B | 0.018 | 0.047 | **0.935** | 0.4762222 |
| Sequence | 131 | P | E | 0.004 | 0.197 | **0.799** | 0.4601111 |
| Sequence | 132 | V | B | 0.003 | **0.718** | 0.279 | 0.4398889 |
| Sequence | 133 | T | B | 0.001 | **0.9** | 0.099 | 0.411 |
| Sequence | 134 | I | B | 0.001 | **0.959** | 0.04 | 0.403 |
| Sequence | 135 | G | B | 0.001 | **0.9** | 0.099 | 0.3907778 |
| Sequence | 136 | G | B | 0.003 | **0.718** | 0.279 | 0.3694444 |
| Sequence | 137 | S | E | 0.002 | **0.816** | 0.182 | 0.3738889 |
| Sequence | 138 | I | B | 0.002 | **0.816** | 0.182 | 0.3686667 |
| Sequence | 139 | F | E | 0.002 | **0.816** | 0.182 | 0.3992222 |
| Sequence | 140 | M | B | 0.002 | **0.816** | 0.182 | 0.4055556 |
| Sequence | 141 | T | E | 0.001 | **0.9** | 0.099 | 0.4143333 |
| Sequence | 142 | L | B | 0.001 | **0.9** | 0.099 | 0.4271111 |
| Sequence | 143 | R | E | 0.018 | **0.846** | 0.136 | 0.4306667 |
| Sequence | 144 | N | E | 0.023 | **0.655** | 0.322 | 0.4352222 |
| Sequence | 145 | V | B | 0.023 | **0.655** | 0.322 | 0.4668889 |
| Sequence | 146 | T | E | 0.023 | **0.655** | 0.322 | 0.4896667 |
| Sequence | 147 | A | B | 0.023 | **0.655** | 0.322 | **0.4985556** |
| Sequence | 148 | V | B | 0.022 | **0.552** | 0.426 | **0.5003333** |
| Sequence | 149 | D | E | 0.069 | 0.386 | **0.545** | **0.5071111** |
| Sequence | 150 | P | B | 0.066 | 0.296 | **0.638** | **0.5114444** |
| Sequence | 151 | I | B | 0.069 | 0.386 | **0.545** | **0.5115556** |
| Sequence | 152 | F | B | 0.022 | 0.359 | **0.619** | **0.511** |
| Sequence | 153 | G | B | 0.021 | 0.279 | **0.699** | **0.5156667** |
| Sequence | 154 | G | E | 0.02 | 0.205 | **0.775** | 0.4816667 |
| Sequence | 155 | S | B | 0.022 | 0.359 | **0.619** | 0.4691111 |
| Sequence | 156 | V | B | 0.004 | **0.616** | 0.381 | 0.4486667 |
| Sequence | 157 | D | B | 0.003 | **0.718** | 0.279 | 0.4323333 |
| Sequence | 158 | V | B | 0.002 | **0.816** | 0.182 | 0.4136667 |
| Sequence | 159 | R | B | 0.001 | **0.9** | 0.099 | 0.4196667 |
| Sequence | 160 | F | B | 0.011 | **0.918** | 0.071 | 0.4297778 |
| Sequence | 161 | F | B | 0.011 | **0.918** | 0.071 | 0.4504444 |
| Sequence | 162 | A | B | 0.021 | **0.756** | 0.223 | 0.4622222 |
| Sequence | 163 | Q | E | 0.022 | **0.552** | 0.426 | **0.5027778** |
| Sequence | 164 | Q | E | 0.022 | 0.359 | **0.619** | **0.5235556** |
| Sequence | 165 | Q | E | 0.019 | 0.141 | **0.84** | **0.5671111** |
| Sequence | 166 | P | B | 0.018 | 0.047 | **0.935** | **0.6078889** |
| Sequence | 167 | N | E | 0.053 | 0.043 | **0.903** | **0.6374444** |
| Sequence | 168 | E | E | 0.053 | 0.043 | **0.903** | **0.6427778** |
| Sequence | 169 | D | E | 0.113 | 0.087 | **0.8** | **0.6398889** |
| Sequence | 170 | Q | E | 0.066 | 0.296 | **0.638** | **0.6293333** |
| Sequence | 171 | L | B | 0.069 | 0.386 | **0.545** | **0.6305556** |
| Sequence | 172 | V | B | 0.066 | 0.296 | **0.638** | **0.6147778** |
| Sequence | 173 | G | E | 0.019 | 0.141 | **0.84** | **0.6062222** |
| Sequence | 174 | G | E | 0.018 | 0.088 | **0.893** | **0.5807778** |
| Sequence | 175 | D | E | 0.021 | 0.279 | **0.699** | **0.5602222** |
| Sequence | 176 | I | B | 0.021 | 0.451 | **0.528** | **0.544** |
| Sequence | 177 | N | E | 0.023 | **0.655** | 0.322 | **0.5442222** |
| Sequence | 178 | I | B | 0.023 | **0.655** | 0.322 | **0.5501111** |
| Sequence | 179 | N | E | 0.021 | 0.451 | **0.528** | **0.5501111** |
| Sequence | 180 | L | B | 0.021 | 0.451 | **0.528** | **0.5512222** |
| Sequence | 181 | E | E | 0.022 | **0.552** | 0.426 | **0.549** |
| Sequence | 182 | N | E | 0.021 | 0.451 | **0.528** | **0.5376667** |
| Sequence | 183 | V | B | 0.022 | 0.359 | **0.619** | **0.5462222** |
| Sequence | 184 | T | E | 0.021 | 0.279 | **0.699** | **0.5357778** |
| Sequence | 185 | T | E | 0.02 | 0.205 | **0.775** | **0.5297778** |
| Sequence | 186 | P | E | 0.021 | 0.451 | **0.528** | **0.5046667** |
| Sequence | 187 | E | B | 0.023 | **0.655** | 0.322 | 0.4715556 |
| Sequence | 188 | F | B | 0.021 | **0.756** | 0.223 | 0.4565556 |
| Sequence | 189 | Y | B | 0.021 | **0.756** | 0.223 | 0.4408889 |
| Sequence | 190 | G | B | 0.021 | **0.756** | 0.223 | 0.4305556 |
| Sequence | 191 | L | B | 0.018 | **0.846** | 0.136 | 0.4271111 |
| Sequence | 192 | G | B | 0.018 | **0.846** | 0.136 | 0.4143333 |
| Sequence | 193 | Y | B | 0.002 | **0.816** | 0.182 | 0.3988889 |
| Sequence | 194 | A | B | 0.003 | **0.718** | 0.279 | 0.3876667 |
| Sequence | 195 | N | E | 0.004 | 0.42 | **0.576** | 0.3977778 |
| Sequence | 196 | G | B | 0.004 | **0.616** | 0.381 | 0.399 |
| Sequence | 197 | V | E | 0.002 | **0.816** | 0.182 | 0.4096667 |
| Sequence | 198 | I | B | 0.001 | **0.9** | 0.099 | 0.41 |
| Sequence | 199 | P | E | 0.002 | **0.816** | 0.182 | 0.4163333 |
| Sequence | 200 | V | B | 0.002 | **0.816** | 0.182 | 0.4233333 |
| Sequence | 201 | N | E | 0.003 | **0.718** | 0.279 | 0.4323333 |
| Sequence | 202 | V | E | 0.023 | **0.655** | 0.322 | 0.4497778 |
| Sequence | 203 | L | B | 0.022 | **0.552** | 0.426 | 0.4592222 |
| Sequence | 204 | N | E | 0.023 | **0.655** | 0.322 | 0.4534444 |
| Sequence | 205 | R | B | 0.021 | **0.756** | 0.223 | 0.4683333 |
| Sequence | 206 | N | E | 0.018 | **0.846** | 0.136 | 0.4562222 |
| Sequence | 207 | F | B | 0.018 | **0.846** | 0.136 | 0.4527778 |
| Sequence | 208 | L | B | 0.018 | **0.846** | 0.136 | 0.4551111 |
| Sequence | 209 | V | B | 0.021 | **0.756** | 0.223 | 0.444 |
| Sequence | 210 | A | B | 0.023 | **0.655** | 0.322 | 0.4342222 |
| Sequence | 211 | V | B | 0.023 | **0.655** | 0.322 | 0.4194444 |
| Sequence | 212 | Q | E | 0.022 | **0.552** | 0.426 | 0.4127778 |
| Sequence | 213 | G | B | 0.021 | 0.451 | **0.528** | 0.4053333 |
| Sequence | 214 | N | E | 0.022 | **0.552** | 0.426 | 0.3912222 |
| Sequence | 215 | I | B | 0.021 | **0.756** | 0.223 | 0.382 |
| Sequence | 216 | T | B | 0.021 | **0.756** | 0.223 | 0.3945556 |
| Sequence | 217 | T | B | 0.018 | **0.846** | 0.136 | 0.4043333 |
| Sequence | 218 | N | B | 0.021 | **0.756** | 0.223 | 0.4205556 |
| Sequence | 219 | I | B | 0.023 | **0.655** | 0.322 | 0.4498889 |
| Sequence | 220 | S | E | 0.021 | 0.451 | **0.528** | 0.454 |
| Sequence | 221 | N | E | 0.019 | 0.141 | **0.84** | 0.4728889 |
| Sequence | 222 | S | B | 0.018 | 0.047 | **0.935** | **0.5015556** |
| Sequence | 223 | N | E | 0.018 | 0.088 | **0.893** | **0.5186667** |
| Sequence | 224 | I | B | 0.021 | 0.279 | **0.699** | **0.5542222** |
| Sequence | 225 | A | E | 0.022 | 0.359 | **0.619** | **0.5531111** |
| Sequence | 226 | T | E | 0.021 | 0.451 | **0.528** | **0.5451111** |
| Sequence | 227 | V | B | 0.021 | 0.451 | **0.528** | **0.5474444** |
| Sequence | 228 | M | E | 0.021 | 0.451 | **0.528** | **0.5355556** |
| Sequence | 229 | L | B | 0.022 | 0.359 | **0.619** | **0.5543333** |
| Sequence | 230 | G | B | 0.02 | 0.205 | **0.775** | **0.5674444** |
| Sequence | 231 | S | E | 0.019 | 0.141 | **0.84** | **0.5645556** |
| Sequence | 232 | H | B | 0.064 | 0.216 | **0.721** | **0.5781111** |
| Sequence | 233 | Y | B | 0.064 | 0.216 | **0.721** | **0.5838889** |
| Sequence | 234 | D | E | 0.066 | 0.296 | **0.638** | **0.5862222** |
| Sequence | 235 | T | B | 0.064 | 0.216 | **0.721** | **0.5986667** |
| Sequence | 236 | T | E | 0.056 | 0.142 | **0.802** | **0.6026667** |
| Sequence | 237 | M | E | 0.056 | 0.142 | **0.802** | **0.5987778** |
| Sequence | 238 | A | B | 0.064 | 0.216 | **0.721** | **0.5976667** |
| Sequence | 239 | V | B | 0.02 | 0.205 | **0.775** | **0.5763333** |
| Sequence | 240 | G | E | 0.019 | 0.141 | **0.84** | **0.562** |
| Sequence | 241 | G | E | 0.019 | 0.141 | **0.84** | **0.5276667** |
| Sequence | 242 | N | E | 0.019 | 0.141 | **0.84** | **0.5034444** |
| Sequence | 243 | G | B | 0.004 | **0.514** | 0.481 | 0.4783333 |
| Sequence | 244 | T | B | 0.002 | **0.816** | 0.182 | 0.4667778 |
| Sequence | 245 | I | B | 0.011 | **0.918** | 0.071 | 0.4606667 |
| Sequence | 246 | N | E | 0.011 | **0.918** | 0.071 | 0.4581111 |
| Sequence | 247 | V | B | 0.003 | **0.718** | 0.279 | 0.4437778 |
| Sequence | 248 | D | E | 0.004 | 0.42 | **0.576** | 0.4348889 |
| Sequence | 249 | N | E | 0.004 | 0.085 | **0.91** | 0.4317778 |
| Sequence | 250 | S | E | 0.019 | 0.141 | **0.84** | 0.436 |
| Sequence | 251 | T | E | 0.004 | **0.514** | 0.481 | 0.4133333 |
| Sequence | 252 | I | B | 0.004 | **0.616** | 0.381 | 0.4188889 |
| Sequence | 253 | G | B | 0.021 | 0.451 | **0.528** | 0.3867778 |
| Sequence | 254 | Y | B | 0.021 | **0.756** | 0.223 | 0.37 |
| Sequence | 255 | L | B | 0.021 | **0.756** | 0.223 | 0.3698889 |
| Sequence | 256 | S | B | 0.021 | **0.756** | 0.223 | 0.3843333 |
| Sequence | 257 | A | B | 0.022 | **0.552** | 0.426 | 0.4038889 |
| Sequence | 258 | S | B | 0.02 | 0.205 | **0.775** | 0.4396667 |
| Sequence | 259 | N | B | 0.019 | 0.141 | **0.84** | 0.4657778 |
| Sequence | 260 | S | E | 0.056 | 0.142 | **0.802** | **0.5102222** |
| Sequence | 261 | S | B | 0.113 | 0.087 | **0.8** | **0.52** |
| Sequence | 262 | D | E | 0.113 | 0.087 | **0.8** | **0.5641111** |
| Sequence | 263 | F | B | 0.064 | 0.216 | **0.721** | **0.5896667** |
| Sequence | 264 | V | E | 0.064 | 0.216 | **0.721** | **0.5845556** |
| Sequence | 265 | N | E | 0.052 | 0.084 | **0.864** | **0.5905556** |
| Sequence | 266 | P | E | 0.113 | 0.043 | **0.844** | **0.584** |
| Sequence | 267 | D | E | 0.113 | 0.043 | **0.844** | **0.5531111** |
| Sequence | 268 | L | B | 0.052 | 0.084 | **0.864** | **0.5423333** |
| Sequence | 269 | T | E | 0.064 | 0.216 | **0.721** | **0.5234444** |
| Sequence | 270 | N | E | 0.021 | 0.451 | **0.528** | **0.5058889** |
| Sequence | 271 | T | B | 0.003 | **0.718** | 0.279 | **0.4912222** |
| Sequence | 272 | V | B | 0.011 | **0.918** | 0.071 | 0.4618889 |
| Sequence | 273 | T | B | 0.011 | **0.918** | 0.071 | 0.4685556 |
| Sequence | 274 | F | B | 0.003 | **0.718** | 0.279 | 0.4697778 |
| Sequence | 275 | N | E | 0.004 | **0.514** | 0.481 | 0.4844444 |
| Sequence | 276 | I | B | 0.004 | 0.138 | **0.858** | **0.5113333** |
| Sequence | 277 | G | E | 0.005 | 0.045 | **0.951** | **0.5338889** |
| Sequence | 278 | P | E | 0.018 | 0.047 | **0.935** | **0.5324444** |
| Sequence | 279 | N | E | 0.058 | 0.017 | **0.925** | **0.5715556** |
| Sequence | 280 | N | B | 0.058 | 0.017 | **0.925** | **0.5897778** |
| Sequence | 281 | R | E | 0.056 | 0.142 | **0.802** | **0.6164444** |
| Sequence | 282 | I | B | 0.066 | 0.296 | **0.638** | **0.6357778** |
| Sequence | 283 | A | E | 0.125 | 0.227 | **0.648** | **0.6438889** |
| Sequence | 284 | N | E | 0.125 | 0.227 | **0.648** | **0.6416667** |
| Sequence | 285 | I | B | 0.125 | 0.227 | **0.648** | **0.6346667** |
| Sequence | 286 | F | E | 0.125 | 0.227 | **0.648** | **0.6218889** |
| Sequence | 287 | A | E | 0.056 | 0.142 | **0.802** | **0.6268889** |
| Sequence | 288 | S | B | 0.018 | 0.047 | **0.935** | **0.607** |
| Sequence | 289 | N | E | 0.018 | 0.047 | **0.935** | **0.581** |
| Sequence | 290 | N | E | 0.018 | 0.047 | **0.935** | **0.5721111** |
| Sequence | 291 | G | B | 0.018 | 0.088 | **0.893** | **0.5536667** |
| Sequence | 292 | V | E | 0.02 | 0.205 | **0.775** | **0.5234444** |
| Sequence | 293 | I | B | 0.021 | 0.279 | **0.699** | **0.5131111** |
| Sequence | 294 | P | E | 0.019 | 0.141 | **0.84** | 0.4702222 |
| Sequence | 295 | H | E | 0.019 | 0.141 | **0.84** | 0.4535556 |
| Sequence | 296 | F | B | 0.004 | **0.514** | 0.481 | 0.4422222 |
| Sequence | 297 | I | B | 0.002 | **0.816** | 0.182 | 0.4562222 |
| Sequence | 298 | V | B | 0.011 | **0.918** | 0.071 | 0.4703333 |
| Sequence | 299 | N | B | 0.018 | **0.846** | 0.136 | 0.478 |
| Sequence | 300 | M | B | 0.023 | **0.655** | 0.322 | 0.4705556 |
| Sequence | 301 | D | E | 0.021 | 0.451 | **0.528** | 0.4851111 |
| Sequence | 302 | G | B | 0.021 | 0.279 | **0.699** | 0.4848889 |
| Sequence | 303 | S | E | 0.022 | 0.359 | **0.619** | **0.5014444** |
| Sequence | 304 | G | B | 0.021 | 0.451 | **0.528** | **0.5107778** |
| Sequence | 305 | T | E | 0.022 | **0.552** | 0.426 | **0.5232222** |
| Sequence | 306 | E | E | 0.023 | **0.655** | 0.322 | **0.4928889** |
| Sequence | 307 | I | B | 0.079 | **0.592** | 0.329 | **0.4964444** |
| Sequence | 308 | Q | E | 0.079 | **0.592** | 0.329 | 0.4755556 |
| Sequence | 309 | E | E | 0.079 | **0.592** | 0.329 | 0.4735556 |
| Sequence | 310 | L | B | 0.074 | 0.484 | 0.442 | 0.4781111 |
| Sequence | 311 | T | E | 0.069 | 0.386 | **0.545** | 0.4783333 |
| Sequence | 312 | L | B | 0.125 | 0.227 | **0.648** | **0.5005556** |
| Sequence | 313 | G | B | 0.199 | 0.152 | **0.649** | **0.5157778** |
| Sequence | 314 | N | E | 0.191 | 0.086 | **0.723** | **0.516** |
| Sequence | 315 | V | B | 0.321 | 0.252 | 0.427 | **0.5392222** |
| Sequence | 316 | I | B | 0.216 | 0.235 | **0.548** | **0.5266667** |
| Sequence | 317 | R | E | 0.216 | 0.235 | **0.548** | **0.5354444** |
| Sequence | 318 | G | E | 0.125 | 0.227 | **0.648** | **0.532** |
| Sequence | 319 | G | B | 0.066 | 0.296 | **0.638** | **0.5054444** |
| Sequence | 320 | L | B | 0.023 | **0.655** | 0.322 | **0.5051111** |
| Sequence | 321 | V | B | 0.018 | **0.846** | 0.136 | 0.4851111 |
| Sequence | 322 | L | B | 0.018 | **0.846** | 0.136 | 0.451 |
| Sequence | 323 | T | E | 0.021 | **0.756** | 0.223 | 0.4517778 |
| Sequence | 324 | S | B | 0.021 | **0.756** | 0.223 | 0.4341111 |
| Sequence | 325 | E | E | 0.018 | **0.846** | 0.136 | 0.4474444 |
| Sequence | 326 | L | B | 0.018 | **0.846** | 0.136 | 0.4595556 |
| Sequence | 327 | N | E | 0.021 | **0.756** | 0.223 | 0.4651111 |
| Sequence | 328 | L | B | 0.021 | 0.451 | **0.528** | 0.4826667 |
| Sequence | 329 | S | E | 0.019 | 0.141 | **0.84** | 0.4658889 |
| Sequence | 330 | Q | E | 0.053 | 0.043 | **0.903** | 0.4805556 |
| Sequence | 331 | G | B | 0.118 | 0.15 | **0.732** | **0.5014444** |
| Sequence | 332 | T | B | 0.148 | 0.418 | 0.435 | 0.4898889 |
| Sequence | 333 | I | B | 0.159 | **0.516** | 0.325 | **0.503** |
| Sequence | 334 | N | E | 0.148 | 0.418 | 0.435 | **0.4901111** |
| Sequence | 335 | N | B | 0.135 | 0.317 | **0.548** | 0.4677778 |
| Sequence | 336 | L | B | 0.148 | 0.418 | 0.435 | 0.4852222 |
| Sequence | 337 | I | E | 0.159 | **0.516** | 0.325 | **0.4978889** |
| Sequence | 338 | T | B | 0.148 | 0.418 | 0.435 | **0.5295556** |
| Sequence | 339 | G | B | 0.125 | 0.227 | **0.648** | **0.5338889** |
| Sequence | 340 | N | E | 0.118 | 0.15 | **0.732** | **0.5333333** |
| Sequence | 341 | E | E | 0.118 | 0.15 | **0.732** | **0.544** |
| Sequence | 342 | Y | E | 0.056 | 0.142 | **0.802** | **0.542** |
| Sequence | 343 | Y | B | 0.052 | 0.084 | **0.864** | **0.5307778** |
| Sequence | 344 | D | E | 0.018 | 0.047 | **0.935** | **0.5146667** |
| Sequence | 345 | R | B | 0.02 | 0.205 | **0.775** | 0.4852222 |
| Sequence | 346 | S | B | 0.022 | **0.552** | 0.426 | 0.4514444 |
| Sequence | 347 | G | B | 0.018 | **0.846** | 0.136 | 0.4193333 |
| Sequence | 348 | L | B | 0.018 | **0.846** | 0.136 | 0.3867778 |
| Sequence | 349 | R | B | 0.001 | **0.9** | 0.099 | 0.3703333 |
| Sequence | 350 | T | B | 0.001 | **0.9** | 0.099 | 0.349 |
| Sequence | 351 | T | E | 0.001 | **0.9** | 0.099 | 0.3595556 |
| Sequence | 352 | V | B | 0.001 | **0.9** | 0.099 | 0.3735556 |
| Sequence | 353 | N | E | 0.002 | **0.816** | 0.182 | 0.3907778 |
| Sequence | 354 | V | B | 0.004 | **0.616** | 0.381 | 0.3972222 |
| Sequence | 355 | R | E | 0.004 | 0.197 | **0.799** | 0.3977778 |
| Sequence | 356 | G | E | 0.005 | 0.045 | **0.951** | 0.3861111 |
| Sequence | 357 | G | B | 0.004 | 0.197 | **0.799** | 0.3736667 |
| Sequence | 358 | T | B | 0.003 | **0.718** | 0.279 | 0.3505556 |
| Sequence | 359 | I | B | 0.011 | **0.918** | 0.071 | 0.3604444 |
| Sequence | 360 | G | B | 0.011 | **0.918** | 0.071 | 0.345 |
| Sequence | 361 | V | B | 0.018 | **0.846** | 0.136 | 0.3547778 |
| Sequence | 362 | L | B | 0.021 | **0.756** | 0.223 | 0.3778889 |
| Sequence | 363 | T | B | 0.021 | 0.451 | **0.528** | 0.4074444 |
| Sequence | 364 | S | B | 0.019 | 0.141 | **0.84** | 0.4401111 |
| Sequence | 365 | G | E | 0.018 | 0.047 | **0.935** | 0.4686667 |
| Sequence | 366 | G | E | 0.018 | 0.047 | **0.935** | **0.5097778** |
| Sequence | 367 | S | E | 0.018 | 0.088 | **0.893** | **0.5476667** |
| Sequence | 368 | D | E | 0.019 | 0.141 | **0.84** | **0.5407778** |
| Sequence | 369 | Y | B | 0.021 | 0.279 | **0.699** | **0.5501111** |
| Sequence | 370 | S | E | 0.022 | 0.359 | **0.619** | **0.5181111** |
| Sequence | 371 | E | E | 0.023 | **0.655** | 0.322 | **0.4961111** |
| Sequence | 372 | L | B | 0.021 | **0.756** | 0.223 | 0.4826667 |
| Sequence | 373 | N | B | 0.021 | **0.756** | 0.223 | 0.4732222 |
| Sequence | 374 | F | B | 0.023 | **0.655** | 0.322 | 0.4815556 |
| Sequence | 375 | I | B | 0.004 | 0.42 | **0.576** | 0.4804444 |
| Sequence | 376 | P | E | 0.019 | 0.141 | **0.84** | 0.477 |
| Sequence | 377 | G | E | 0.018 | 0.019 | **0.964** | 0.4878889 |
| Sequence | 378 | E | E | 0.056 | 0.142 | **0.802** | 0.4686667 |
| Sequence | 379 | I | B | 0.021 | 0.451 | **0.528** | **0.4901111** |
| Sequence | 380 | S | B | 0.023 | **0.655** | 0.322 | **0.4954444** |
| Sequence | 381 | T | E | 0.021 | **0.756** | 0.223 | **0.494** |
| Sequence | 382 | I | B | 0.018 | **0.846** | 0.136 | **0.5027778** |
| Sequence | 383 | L | E | 0.021 | **0.756** | 0.223 | **0.5007778** |
| Sequence | 384 | A | B | 0.022 | **0.552** | 0.426 | **0.4923333** |
| Sequence | 385 | T | E | 0.021 | 0.451 | **0.528** | **0.5055556** |
| Sequence | 386 | N | E | 0.021 | 0.451 | **0.528** | **0.5202222** |
| Sequence | 387 | S | E | 0.021 | 0.451 | **0.528** | **0.5518889** |
| Sequence | 388 | I | B | 0.022 | 0.359 | **0.619** | **0.5701111** |
| Sequence | 389 | G | E | 0.02 | 0.205 | **0.775** | **0.57** |
| Sequence | 390 | N | E | 0.018 | 0.047 | **0.935** | **0.5777778** |
| Sequence | 391 | Q | E | 0.018 | 0.047 | **0.935** | **0.5633333** |
| Sequence | 392 | D | E | 0.018 | 0.047 | **0.935** | **0.5534444** |
| Sequence | 393 | F | B | 0.019 | 0.141 | **0.84** | **0.5664444** |
| Sequence | 394 | A | B | 0.019 | 0.141 | **0.84** | **0.5515556** |
| Sequence | 395 | S | B | 0.021 | 0.279 | **0.699** | **0.5186667** |
| Sequence | 396 | L | B | 0.021 | 0.451 | **0.528** | **0.5013333** |
| Sequence | 397 | S | B | 0.021 | 0.451 | **0.528** | 0.4574444 |
| Sequence | 398 | Q | E | 0.079 | **0.592** | 0.329 | 0.4574444 |
| Sequence | 399 | V | B | 0.085 | **0.775** | 0.14 | 0.4546667 |
| Sequence | 400 | T | E | 0.085 | **0.775** | 0.14 | 0.4568889 |
| Sequence | 401 | I | B | 0.273 | **0.587** | 0.14 | 0.463 |
| Sequence | 402 | H | E | 0.352 | 0.332 | 0.316 | 0.4597778 |
| Sequence | 403 | Q | E | 0.307 | 0.165 | **0.527** | 0.4675556 |
| Sequence | 404 | G | B | 0.386 | 0.097 | **0.517** | 0.4892222 |
| Sequence | 405 | A | B | 0.428 | 0.171 | 0.402 | **0.4995556** |
| Sequence | 406 | E | E | 0.478 | 0.309 | 0.214 | **0.5275556** |
| Sequence | 407 | T | B | 0.373 | 0.401 | 0.226 | **0.5307778** |
| Sequence | 408 | L | B | 0.252 | 0.423 | 0.325 | **0.5354444** |
| Sequence | 409 | W | B | 0.231 | 0.33 | 0.439 | **0.5362222** |
| Sequence | 410 | G | B | 0.064 | 0.216 | **0.721** | **0.5321111** |
| Sequence | 411 | M | B | 0.018 | 0.047 | **0.935** | **0.5286667** |
| Sequence | 412 | R | E | 0.018 | 0.019 | **0.964** | **0.5065556** |
| Sequence | 413 | D | E | 0.018 | 0.019 | **0.964** | **0.518** |
| Sequence | 414 | E | B | 0.018 | 0.088 | **0.893** | 0.4841111 |
| Sequence | 415 | V | E | 0.021 | 0.451 | **0.528** | 0.4865556 |
| Sequence | 416 | F | B | 0.021 | **0.756** | 0.223 | 0.4862222 |
| Sequence | 417 | E | E | 0.018 | **0.846** | 0.136 | 0.4744444 |
| Sequence | 418 | L | B | 0.018 | **0.846** | 0.136 | 0.4883333 |
| Sequence | 419 | Q | E | 0.021 | **0.756** | 0.223 | 0.4847778 |
| Sequence | 420 | T | B | 0.022 | 0.359 | **0.619** | 0.4895556 |
| Sequence | 421 | N | E | 0.02 | 0.205 | **0.775** | **0.5003333** |
| Sequence | 422 | N | E | 0.02 | 0.205 | **0.775** | 0.4827778 |
| Sequence | 423 | L | B | 0.021 | 0.451 | **0.528** | **0.4936667** |
| Sequence | 424 | Q | E | 0.023 | **0.655** | 0.322 | 0.4821111 |
| Sequence | 425 | L | B | 0.004 | **0.514** | 0.481 | 0.4644444 |
| Sequence | 426 | G | B | 0.005 | 0.336 | **0.66** | 0.4406667 |
| Sequence | 427 | G | B | 0.004 | 0.42 | **0.576** | 0.399 |
| Sequence | 428 | E | E | 0.001 | **0.9** | 0.099 | 0.4044444 |
| Sequence | 429 | L | B | 0.001 | **0.9** | 0.099 | 0.3923333 |
| Sequence | 430 | F | B | 0.018 | **0.846** | 0.136 | 0.4084444 |
| Sequence | 431 | I | B | 0.004 | **0.616** | 0.381 | 0.4253333 |
| Sequence | 432 | P | B | 0.004 | 0.42 | **0.576** | 0.4461111 |
| Sequence | 433 | A | B | 0.019 | 0.141 | **0.84** | 0.454 |
| Sequence | 434 | D | E | 0.052 | 0.084 | **0.864** | 0.469 |
| Sequence | 435 | G | E | 0.052 | 0.084 | **0.864** | 0.4802222 |
| Sequence | 436 | T | B | 0.113 | 0.087 | **0.8** | 0.488 |
| Sequence | 437 | G | B | 0.191 | 0.086 | **0.723** | 0.478 |
| Sequence | 438 | G | B | 0.199 | 0.152 | **0.649** | 0.4582222 |
| Sequence | 439 | V | B | 0.352 | 0.332 | 0.316 | 0.4357778 |
| Sequence | 440 | A | B | 0.391 | 0.466 | 0.143 | 0.411 |
| Sequence | 441 | L | B | 0.373 | 0.401 | 0.226 | 0.3846667 |
| Sequence | 442 | I | B | 0.268 | **0.505** | 0.227 | 0.3538889 |
| Sequence | 443 | T | B | 0.252 | 0.423 | 0.325 | 0.3233333 |
| Sequence | 444 | N | B | 0.216 | 0.235 | **0.548** | 0.3106667 |
| Sequence | 445 | H | B | 0.159 | **0.516** | 0.325 | 0.3295556 |
| Sequence | 446 | I | B | 0.172 | **0.69** | 0.138 | 0.3346667 |
| Sequence | 447 | I | B | 0.087 | **0.683** | 0.23 | 0.3447778 |
| Sequence | 448 | A | B | 0.069 | 0.386 | **0.545** | 0.3415556 |
| Sequence | 449 | N | E | 0.018 | 0.088 | **0.893** | 0.3411111 |
| Sequence | 450 | S | E | 0.018 | 0.047 | **0.935** | 0.3548889 |
| Sequence | 451 | G | B | 0.004 | 0.138 | **0.858** | 0.3763333 |
| Sequence | 452 | V | B | 0.003 | **0.718** | 0.279 | 0.4112222 |
| Sequence | 453 | I | B | 0.018 | **0.846** | 0.136 | 0.4426667 |
| Sequence | 454 | T | E | 0.021 | **0.756** | 0.223 | 0.4441111 |
| Sequence | 455 | P | B | 0.023 | **0.655** | 0.322 | 0.4604444 |
| Sequence | 456 | V | E | 0.022 | **0.552** | 0.426 | 0.4898889 |
| Sequence | 457 | N | E | 0.022 | 0.359 | **0.619** | **0.5193333** |
| Sequence | 458 | M | B | 0.019 | 0.141 | **0.84** | **0.5516667** |
| Sequence | 459 | S | E | 0.018 | 0.088 | **0.893** | **0.5521111** |
| Sequence | 460 | P | E | 0.018 | 0.047 | **0.935** | **0.5552222** |
| Sequence | 461 | E | E | 0.018 | 0.019 | **0.964** | **0.5436667** |
| Sequence | 462 | R | E | 0.018 | 0.088 | **0.893** | **0.5145556** |
| Sequence | 463 | M | B | 0.02 | 0.205 | **0.775** | **0.4926667** |
| Sequence | 464 | T | B | 0.005 | 0.262 | **0.733** | 0.4535556 |
| Sequence | 465 | P | B | 0.004 | 0.42 | **0.576** | 0.4122222 |
| Sequence | 466 | I | B | 0.002 | **0.816** | 0.182 | 0.3857778 |
| Sequence | 467 | I | B | 0.011 | **0.918** | 0.071 | 0.3673333 |
| Sequence | 468 | G | B | 0.006 | **0.962** | 0.032 | 0.3796667 |
| Sequence | 469 | F | B | 0.018 | **0.846** | 0.136 | 0.3947778 |
| Sequence | 470 | L | B | 0.022 | **0.552** | 0.426 | 0.4142222 |
| Sequence | 471 | E | B | 0.004 | 0.197 | **0.799** | 0.4513333 |
| Sequence | 472 | P | E | 0.005 | 0.045 | **0.951** | 0.4795556 |
| Sequence | 473 | T | E | 0.018 | 0.019 | **0.964** | **0.5028889** |
| Sequence | 474 | G | E | 0.018 | 0.019 | **0.964** | **0.5285556** |
| Sequence | 475 | E | E | 0.018 | 0.088 | **0.893** | **0.52** |
| Sequence | 476 | V | E | 0.022 | 0.359 | **0.619** | **0.5193333** |
| Sequence | 477 | A | B | 0.021 | 0.451 | **0.528** | 0.481 |
| Sequence | 478 | Q | E | 0.023 | **0.655** | 0.322 | 0.4681111 |
| Sequence | 479 | L | B | 0.018 | **0.846** | 0.136 | 0.4591111 |
| Sequence | 480 | T | B | 0.011 | **0.918** | 0.071 | 0.438 |
| Sequence | 481 | I | B | 0.018 | **0.846** | 0.136 | 0.4334444 |
| Sequence | 482 | Y | B | 0.021 | **0.756** | 0.223 | 0.4365556 |
| Sequence | 483 | G | E | 0.022 | 0.359 | **0.619** | 0.4255556 |
| Sequence | 484 | P | E | 0.02 | 0.205 | **0.775** | 0.4502222 |
| Sequence | 485 | L | B | 0.021 | 0.451 | **0.528** | 0.4542222 |
| Sequence | 486 | T | E | 0.023 | **0.655** | 0.322 | **0.4973333** |
| Sequence | 487 | V | B | 0.023 | **0.655** | 0.322 | **0.5065556** |
| Sequence | 488 | N | E | 0.021 | 0.451 | **0.528** | **0.5128889** |
| Sequence | 489 | L | B | 0.021 | 0.279 | **0.699** | **0.5205556** |
| Sequence | 490 | S | E | 0.019 | 0.141 | **0.84** | **0.5265556** |
| Sequence | 491 | H | B | 0.053 | 0.043 | **0.903** | **0.5271111** |
| Sequence | 492 | S | E | 0.058 | 0.017 | **0.925** | **0.5265556** |
| Sequence | 493 | P | B | 0.354 | 0.048 | **0.598** | **0.526** |
| Sequence | 494 | E | E | 0.386 | 0.097 | **0.517** | **0.5421111** |
| Sequence | 495 | I | B | 0.428 | 0.171 | 0.402 | **0.5387778** |
| Sequence | 496 | L | B | 0.428 | 0.171 | 0.402 | **0.5541111** |
| Sequence | 497 | G | B | 0.199 | 0.152 | **0.649** | **0.5491111** |
| Sequence | 498 | K | E | 0.199 | 0.152 | **0.649** | **0.5468889** |
| Sequence | 499 | I | E | 0.199 | 0.152 | **0.649** | **0.5424444** |
| Sequence | 500 | I | E | 0.113 | 0.087 | **0.8** | **0.5304444** |
| Sequence | 501 | T | E | 0.113 | 0.087 | **0.8** | **0.5413333** |
| Sequence | 502 | Q | E | 0.052 | 0.084 | **0.864** | **0.5058889** |
| Sequence | 503 | P | B | 0.019 | 0.141 | **0.84** | 0.4575556 |
| Sequence | 504 | I | B | 0.022 | 0.359 | **0.619** | 0.4258889 |
| Sequence | 505 | P | B | 0.023 | **0.655** | 0.322 | 0.3831111 |
| Sequence | 506 | I | B | 0.021 | **0.756** | 0.223 | 0.3793333 |
| Sequence | 507 | A | B | 0.018 | **0.846** | 0.136 | 0.3808889 |
| Sequence | 508 | V | B | 0.021 | **0.756** | 0.223 | 0.3921111 |
| Sequence | 509 | T | B | 0.023 | **0.655** | 0.322 | 0.4175556 |
| Sequence | 510 | N | B | 0.02 | 0.205 | **0.775** | 0.4293333 |
| Sequence | 511 | S | E | 0.053 | 0.043 | **0.903** | 0.4784444 |
| Sequence | 512 | D | E | 0.053 | 0.043 | **0.903** | **0.5087778** |
| Sequence | 513 | V | B | 0.052 | 0.084 | **0.864** | **0.5434444** |
| Sequence | 514 | F | B | 0.052 | 0.084 | **0.864** | **0.5833333** |
| Sequence | 515 | G | E | 0.018 | 0.047 | **0.935** | **0.5853333** |
| Sequence | 516 | T | E | 0.058 | 0.017 | **0.925** | **0.5818889** |
| Sequence | 517 | S | E | 0.184 | 0.043 | **0.773** | **0.5687778** |
| Sequence | 518 | K | E | 0.354 | 0.048 | **0.598** | **0.5568889** |
| Sequence | 519 | L | B | 0.386 | 0.097 | **0.517** | **0.552** |
| Sequence | 520 | F | B | **0.502** | 0.102 | 0.396 | **0.5464444** |
| Sequence | 521 | V | E | **0.605** | 0.105 | 0.29 | **0.5574444** |
| Sequence | 522 | E | E | **0.502** | 0.102 | 0.396 | **0.5573333** |
| Sequence | 523 | H | B | **0.538** | 0.173 | 0.289 | **0.5324444** |
| Sequence | 524 | N | B | 0.428 | 0.171 | 0.402 | **0.5192222** |
| Sequence | 525 | T | E | 0.191 | 0.086 | **0.723** | **0.5095556** |
| Sequence | 526 | K | E | 0.113 | 0.087 | **0.8** | **0.4931111** |
| Sequence | 527 | G | B | 0.064 | 0.216 | **0.721** | 0.4765556 |
| Sequence | 528 | L | B | 0.021 | 0.451 | **0.528** | 0.452 |
| Sequence | 529 | I | B | 0.023 | **0.655** | 0.322 | 0.423 |
| Sequence | 530 | W | B | 0.023 | **0.655** | 0.322 | 0.4093333 |
| Sequence | 531 | S | B | 0.022 | **0.552** | 0.426 | 0.3795556 |
| Sequence | 532 | D | B | 0.022 | **0.552** | 0.426 | 0.3877778 |
| Sequence | 533 | I | B | 0.021 | **0.756** | 0.223 | 0.4151111 |
| Sequence | 534 | I | E | 0.018 | **0.846** | 0.136 | 0.4386667 |
| Sequence | 535 | F | B | 0.021 | **0.756** | 0.223 | 0.4581111 |
| Sequence | 536 | N | E | 0.005 | 0.262 | **0.733** | 0.4767778 |
| Sequence | 537 | P | E | 0.018 | 0.088 | **0.893** | 0.4898889 |
| Sequence | 538 | Q | E | 0.018 | 0.088 | **0.893** | 0.49 |
| Sequence | 539 | D | E | 0.018 | 0.088 | **0.893** | 0.4882222 |
| Sequence | 540 | K | E | 0.064 | 0.216 | **0.721** | 0.4827778 |
| Sequence | 541 | T | B | 0.023 | **0.655** | 0.322 | 0.4827778 |
| Sequence | 542 | W | B | 0.018 | **0.846** | 0.136 | 0.4697778 |
| Sequence | 543 | Y | B | 0.018 | **0.846** | 0.136 | 0.4537778 |
| Sequence | 544 | L | B | 0.085 | **0.775** | 0.14 | 0.4623333 |
| Sequence | 545 | T | B | 0.074 | 0.484 | 0.442 | 0.4706667 |
| Sequence | 546 | N | B | 0.069 | 0.386 | **0.545** | 0.484 |
| Sequence | 547 | F | B | 0.069 | 0.386 | **0.545** | **0.5142222** |
| Sequence | 548 | R | E | 0.135 | 0.317 | **0.548** | **0.534** |
| Sequence | 549 | G | E | 0.125 | 0.227 | **0.648** | **0.5512222** |
| Sequence | 550 | S | B | 0.191 | 0.086 | **0.723** | **0.5595556** |
| Sequence | 551 | E | E | 0.191 | 0.086 | **0.723** | **0.5476667** |
| Sequence | 552 | D | E | 0.307 | 0.165 | **0.527** | **0.5372222** |
| Sequence | 553 | F | B | 0.307 | 0.165 | **0.527** | **0.5115556** |
| Sequence | 554 | Y | B | 0.428 | 0.171 | 0.402 | **0.4918889** |
| Sequence | 555 | G | B | 0.428 | 0.171 | 0.402 | 0.4571111 |
| Sequence | 556 | L | B | **0.538** | 0.173 | 0.289 | 0.4492222 |
| Sequence | 557 | S | B | **0.605** | 0.105 | 0.29 | 0.4453333 |
| Sequence | 558 | A | B | **0.701** | 0.107 | 0.192 | 0.4515556 |
| Sequence | 559 | A | B | **0.751** | 0.05 | 0.199 | 0.4408889 |
| Sequence | 560 | R | E | **0.751** | 0.05 | 0.199 | 0.4584444 |
| Sequence | 561 | E | E | **0.717** | 0.014 | 0.269 | 0.4832222 |
| Sequence | 562 | A | B | **0.717** | 0.014 | 0.269 | 0.4705556 |
| Sequence | 563 | S | B | **0.622** | 0.015 | 0.363 | 0.4785556 |
| Sequence | 564 | N | E | **0.717** | 0.014 | 0.269 | **0.5116667** |
| Sequence | 565 | W | B | **0.879** | 0.01 | 0.111 | **0.504** |
| Sequence | 566 | L | B | **0.879** | 0.01 | 0.111 | **0.4991111** |
| Sequence | 567 | R | B | **0.879** | 0.01 | 0.111 | **0.4928889** |
| Sequence | 568 | Q | B | **0.879** | 0.01 | 0.111 | **0.4991111** |
| Sequence | 569 | Q | E | **0.802** | 0.014 | 0.185 | 0.4818889 |
| Sequence | 570 | H | B | **0.751** | 0.05 | 0.199 | 0.458 |
| Sequence | 571 | I | B | **0.701** | 0.107 | 0.192 | 0.4826667 |
| Sequence | 572 | W | B | **0.779** | 0.1 | 0.12 | **0.4918889** |
| Sequence | 573 | S | B | **0.831** | 0.044 | 0.125 | **0.4986667** |
| Sequence | 574 | L | B | **0.831** | 0.044 | 0.125 | **0.5066667** |
| Sequence | 575 | Q | B | **0.802** | 0.014 | 0.185 | **0.5131111** |
| Sequence | 576 | R | E | **0.802** | 0.014 | 0.185 | **0.5296667** |
| Sequence | 577 | R | E | **0.802** | 0.014 | 0.185 | **0.5322222** |
| Sequence | 578 | S | B | **0.717** | 0.014 | 0.269 | **0.5334444** |
| Sequence | 579 | N | E | **0.717** | 0.014 | 0.269 | **0.5595556** |
| Sequence | 580 | K | E | **0.717** | 0.014 | 0.269 | **0.5743333** |
| Sequence | 581 | L | B | **0.622** | 0.015 | 0.363 | **0.5621111** |
| Sequence | 582 | L | B | **0.622** | 0.015 | 0.363 | **0.5368889** |
| Sequence | 583 | D | E | **0.522** | 0.016 | 0.462 | **0.5387778** |
| Sequence | 584 | H | E | 0.339 | 0.016 | **0.645** | **0.5064444** |
| Sequence | 585 | G | E | 0.184 | 0.043 | **0.773** | 0.4704444 |
| Sequence | 586 | V | B | 0.113 | 0.087 | **0.8** | 0.438 |
| Sequence | 587 | D | B | 0.056 | 0.142 | **0.802** | 0.4146667 |
| Sequence | 588 | G | B | 0.074 | 0.484 | 0.442 | 0.3795556 |
| Sequence | 589 | L | B | 0.021 | **0.756** | 0.223 | 0.3381111 |
| Sequence | 590 | W | B | 0.018 | **0.846** | 0.136 | 0.3394444 |
| Sequence | 591 | M | B | 0.018 | **0.846** | 0.136 | 0.3515556 |
| Sequence | 592 | N | B | 0.021 | **0.756** | 0.223 | 0.3448889 |
| Sequence | 593 | V | B | 0.074 | 0.484 | 0.442 | 0.3707778 |
| Sequence | 594 | Q | E | 0.135 | 0.317 | **0.548** | 0.4076667 |
| Sequence | 595 | G | E | 0.191 | 0.086 | **0.723** | 0.4433333 |
| Sequence | 596 | G | B | 0.268 | 0.043 | **0.689** | 0.4673333 |
| Sequence | 597 | Y | B | 0.455 | 0.046 | **0.498** | **0.5066667** |
| Sequence | 598 | E | E | **0.502** | 0.102 | 0.396 | **0.5494444** |
| Sequence | 599 | K | E | **0.605** | 0.105 | 0.29 | **0.5565556** |
| Sequence | 600 | L | B | **0.561** | 0.047 | 0.393 | **0.5661111** |
| Sequence | 601 | D | E | 0.455 | 0.046 | **0.498** | **0.578** |
| Sequence | 602 | A | E | 0.455 | 0.046 | **0.498** | **0.5862222** |
| Sequence | 603 | A | E | 0.354 | 0.048 | **0.598** | **0.5775556** |
| Sequence | 604 | I | B | 0.268 | 0.043 | **0.689** | **0.5782222** |
| Sequence | 605 | G | E | 0.113 | 0.087 | **0.8** | **0.574** |
| Sequence | 606 | D | E | 0.113 | 0.043 | **0.844** | **0.5447778** |
| Sequence | 607 | A | B | 0.113 | 0.043 | **0.844** | **0.5088889** |
| Sequence | 608 | K | E | 0.184 | 0.043 | **0.773** | 0.4701111 |
| Sequence | 609 | M | B | 0.354 | 0.048 | **0.598** | 0.4257778 |
| Sequence | 610 | P | B | **0.561** | 0.047 | 0.393 | 0.3807778 |
| Sequence | 611 | W | B | **0.701** | 0.107 | 0.192 | 0.3477778 |
| Sequence | 612 | I | B | **0.725** | 0.163 | 0.112 | 0.3323333 |
| Sequence | 613 | M | B | **0.725** | 0.163 | 0.112 | 0.3096667 |
| Sequence | 614 | A | B | **0.779** | 0.1 | 0.12 | 0.3206667 |
| Sequence | 615 | S | B | **0.66** | 0.049 | 0.291 | 0.3324444 |
| Sequence | 616 | L | B | 0.455 | 0.046 | **0.498** | 0.344 |
| Sequence | 617 | G | B | 0.181 | 0.016 | **0.803** | 0.3668889 |
| Sequence | 618 | Y | B | **0.522** | 0.016 | 0.462 | 0.406 |
| Sequence | 619 | D | E | **0.802** | 0.014 | 0.185 | 0.4451111 |
| Sequence | 620 | F | B | **0.879** | 0.01 | 0.111 | 0.4545556 |
| Sequence | 621 | M | B | **0.879** | 0.01 | 0.111 | 0.4811111 |
| Sequence | 622 | H | E | **0.802** | 0.014 | 0.185 | **0.5046667** |
| Sequence | 623 | K | E | **0.6** | 0.003 | 0.397 | **0.5127778** |
| Sequence | 624 | L | B | 0.246 | 0.004 | **0.75** | **0.5228889** |
| Sequence | 625 | S | E | 0.109 | 0.005 | **0.886** | **0.5316667** |
| Sequence | 626 | D | E | 0.176 | 0.004 | **0.82** | **0.5268889** |
| Sequence | 627 | F | B | 0.43 | 0.016 | **0.555** | **0.5174444** |
| Sequence | 628 | Y | E | **0.522** | 0.016 | 0.462 | **0.4988889** |
| Sequence | 629 | N | B | **0.522** | 0.016 | 0.462 | 0.4792222 |
| Sequence | 630 | L | B | **0.802** | 0.014 | 0.185 | 0.4433333 |
| Sequence | 631 | K | B | **0.802** | 0.014 | 0.185 | 0.3966667 |
| Sequence | 632 | A | B | **0.802** | 0.014 | 0.185 | 0.3628889 |
| Sequence | 633 | L | B | **0.831** | 0.044 | 0.125 | 0.3184444 |
| Sequence | 634 | Y | B | **0.831** | 0.044 | 0.125 | 0.3005556 |
| Sequence | 635 | G | B | **0.751** | 0.05 | 0.199 | 0.2925556 |
| Sequence | 636 | F | B | **0.605** | 0.105 | 0.29 | 0.2895556 |
| Sequence | 637 | G | B | **0.538** | 0.173 | 0.289 | 0.299 |
| Sequence | 638 | F | B | 0.453 | 0.248 | 0.299 | 0.3362222 |
| Sequence | 639 | G | B | 0.352 | 0.332 | 0.316 | 0.3693333 |
| Sequence | 640 | F | B | 0.231 | 0.33 | 0.439 | 0.4222222 |
| Sequence | 641 | A | B | 0.118 | 0.15 | **0.732** | 0.4626667 |
| Sequence | 642 | T | E | 0.113 | 0.087 | **0.8** | **0.5064444** |
| Sequence | 643 | G | E | 0.053 | 0.043 | **0.903** | **0.5271111** |
| Sequence | 644 | K | E | 0.113 | 0.087 | **0.8** | **0.5627778** |
| Sequence | 645 | N | E | 0.064 | 0.216 | **0.721** | **0.5778889** |
| Sequence | 646 | K | E | 0.066 | 0.296 | **0.638** | **0.5724444** |
| Sequence | 647 | W | B | 0.074 | 0.484 | 0.442 | **0.5854444** |
| Sequence | 648 | N | E | 0.074 | 0.484 | 0.442 | **0.5922222** |
| Sequence | 649 | T | E | 0.159 | **0.516** | 0.325 | **0.5934444** |
| Sequence | 650 | I | B | 0.148 | 0.418 | 0.435 | **0.5837778** |
| Sequence | 651 | N | E | 0.135 | 0.317 | **0.548** | **0.5737778** |
| Sequence | 652 | S | E | 0.125 | 0.227 | **0.648** | **0.5723333** |
| Sequence | 653 | T | E | 0.125 | 0.227 | **0.648** | **0.5462222** |
| Sequence | 654 | T | E | 0.199 | 0.152 | **0.649** | **0.5365556** |
| Sequence | 655 | N | E | 0.199 | 0.152 | **0.649** | **0.52** |
| Sequence | 656 | D | E | 0.231 | 0.33 | 0.439 | 0.4763333 |
| Sequence | 657 | I | B | 0.159 | **0.516** | 0.325 | 0.4363333 |
| Sequence | 658 | Y | B | 0.169 | **0.612** | 0.219 | 0.3958889 |
| Sequence | 659 | M | B | 0.169 | **0.612** | 0.219 | 0.3743333 |
| Sequence | 660 | G | B | 0.252 | 0.423 | 0.325 | 0.3552222 |
| Sequence | 661 | L | B | 0.321 | 0.252 | 0.427 | 0.3245556 |
| Sequence | 662 | V | B | 0.428 | 0.171 | 0.402 | 0.312 |
| Sequence | 663 | G | B | 0.428 | 0.171 | 0.402 | 0.2926667 |
| Sequence | 664 | A | B | 0.428 | 0.171 | 0.402 | 0.2965556 |
| Sequence | 665 | Y | B | 0.453 | 0.248 | 0.299 | 0.3043333 |
| Sequence | 666 | V | B | 0.352 | 0.332 | 0.316 | 0.3371111 |
| Sequence | 667 | G | B | 0.352 | 0.332 | 0.316 | 0.3682222 |
| Sequence | 668 | L | B | 0.231 | 0.33 | 0.439 | 0.3831111 |
| Sequence | 669 | M | B | 0.148 | 0.418 | 0.435 | 0.4097778 |
| Sequence | 670 | H | B | 0.135 | 0.317 | **0.548** | 0.4368889 |
| Sequence | 671 | E | E | 0.118 | 0.15 | **0.732** | 0.45 |
| Sequence | 672 | A | E | 0.113 | 0.087 | **0.8** | 0.4706667 |
| Sequence | 673 | T | B | 0.191 | 0.086 | **0.723** | 0.4764444 |
| Sequence | 674 | G | B | 0.199 | 0.152 | **0.649** | 0.4725556 |
| Sequence | 675 | L | B | 0.307 | 0.165 | **0.527** | 0.4384444 |
| Sequence | 676 | Y | B | 0.352 | 0.332 | 0.316 | 0.4192222 |
| Sequence | 677 | G | B | 0.252 | 0.423 | 0.325 | 0.4085556 |
| Sequence | 678 | T | B | 0.268 | **0.505** | 0.227 | 0.3915556 |
| Sequence | 679 | V | B | 0.169 | **0.612** | 0.219 | 0.3867778 |
| Sequence | 680 | S | B | 0.169 | **0.612** | 0.219 | 0.3954444 |
| Sequence | 681 | G | B | 0.169 | **0.612** | 0.219 | 0.4045556 |
| Sequence | 682 | Q | E | 0.159 | **0.516** | 0.325 | 0.4322222 |
| Sequence | 683 | F | B | 0.252 | 0.423 | 0.325 | 0.4682222 |
| Sequence | 684 | A | B | 0.252 | 0.423 | 0.325 | **0.5028889** |
| Sequence | 685 | T | B | 0.231 | 0.33 | 0.439 | **0.5323333** |
| Sequence | 686 | N | E | 0.135 | 0.317 | **0.548** | **0.5646667** |
| Sequence | 687 | R | E | 0.135 | 0.317 | **0.548** | **0.588** |
| Sequence | 688 | T | B | 0.148 | 0.418 | 0.435 | **0.6008889** |
| Sequence | 689 | K | E | 0.148 | 0.418 | 0.435 | **0.613** |
| Sequence | 690 | T | E | 0.069 | 0.386 | **0.545** | **0.6017778** |
| Sequence | 691 | K | E | 0.056 | 0.142 | **0.802** | **0.5852222** |
| Sequence | 692 | C | B | 0.018 | 0.047 | **0.935** | **0.5762222** |
| Sequence | 693 | T | E | 0.018 | 0.047 | **0.935** | **0.5802222** |
| Sequence | 694 | G | B | 0.056 | 0.142 | **0.802** | **0.5618889** |
| Sequence | 695 | F | B | 0.069 | 0.386 | **0.545** | **0.551** |
| Sequence | 696 | D | B | 0.022 | **0.552** | 0.426 | **0.5453333** |
| Sequence | 697 | E | E | 0.022 | **0.552** | 0.426 | **0.535** |
| Sequence | 698 | T | E | 0.021 | 0.451 | **0.528** | **0.5401111** |
| Sequence | 699 | Y | B | 0.021 | 0.279 | **0.699** | **0.5647778** |
| Sequence | 700 | N | E | 0.022 | 0.359 | **0.619** | **0.5777778** |
| Sequence | 701 | W | B | 0.022 | 0.359 | **0.619** | **0.5793333** |
| Sequence | 702 | K | E | 0.074 | 0.484 | 0.442 | **0.5724444** |
| Sequence | 703 | E | E | 0.069 | 0.386 | **0.545** | **0.5743333** |
| Sequence | 704 | N | E | 0.064 | 0.216 | **0.721** | **0.575** |
| Sequence | 705 | V | B | 0.052 | 0.084 | **0.864** | **0.5658889** |
| Sequence | 706 | P | B | 0.191 | 0.086 | **0.723** | **0.5657778** |
| Sequence | 707 | T | E | 0.278 | 0.093 | **0.628** | **0.554** |
| Sequence | 708 | E | E | 0.307 | 0.165 | **0.527** | **0.5366667** |
| Sequence | 709 | A | B | 0.352 | 0.332 | 0.316 | **0.5234444** |
| Sequence | 710 | I | B | 0.252 | 0.423 | 0.325 | **0.5121111** |
| Sequence | 711 | E | E | 0.268 | **0.505** | 0.227 | **0.5004444** |
| Sequence | 712 | I | B | 0.252 | 0.423 | 0.325 | **0.4996667** |
| Sequence | 713 | G | B | 0.148 | 0.418 | 0.435 | 0.4892222 |
| Sequence | 714 | W | B | 0.159 | **0.516** | 0.325 | **0.4951111** |
| Sequence | 715 | K | E | 0.169 | **0.612** | 0.219 | **0.5044444** |
| Sequence | 716 | W | B | 0.087 | **0.683** | 0.23 | **0.5098889** |
| Sequence | 717 | S | B | 0.079 | **0.592** | 0.329 | **0.5024444** |
| Sequence | 718 | I | B | 0.074 | 0.484 | 0.442 | **0.5166667** |
| Sequence | 719 | D | E | 0.066 | 0.296 | **0.638** | **0.5146667** |
| Sequence | 720 | E | E | 0.022 | 0.359 | **0.619** | **0.5218889** |
| Sequence | 721 | F | B | 0.023 | **0.655** | 0.322 | **0.5276667** |
| Sequence | 722 | K | E | 0.021 | **0.756** | 0.223 | **0.5382222** |
| Sequence | 723 | I | B | 0.023 | **0.655** | 0.322 | **0.5231111** |
| Sequence | 724 | N | E | 0.02 | 0.205 | **0.775** | **0.5096667** |
| Sequence | 725 | P | E | 0.056 | 0.142 | **0.802** | **0.4978889** |
| Sequence | 726 | R | E | 0.118 | 0.15 | **0.732** | 0.4792222 |
| Sequence | 727 | G | B | 0.216 | 0.235 | **0.548** | 0.4474444 |
| Sequence | 728 | Q | E | 0.268 | **0.505** | 0.227 | 0.4533333 |
| Sequence | 729 | V | B | 0.172 | **0.69** | 0.138 | 0.4561111 |
| Sequence | 730 | I | B | 0.273 | **0.587** | 0.14 | 0.4502222 |
| Sequence | 731 | F | B | 0.373 | 0.401 | 0.226 | 0.455 |
| Sequence | 732 | E | E | 0.478 | 0.309 | 0.214 | 0.4802222 |
| Sequence | 733 | Q | E | 0.428 | 0.171 | 0.402 | **0.4938889** |
| Sequence | 734 | L | B | 0.184 | 0.043 | **0.773** | **0.5066667** |
| Sequence | 735 | S | E | 0.115 | 0.016 | **0.868** | **0.5161111** |
| Sequence | 736 | K | E | 0.113 | 0.043 | **0.844** | **0.5385556** |
| Sequence | 737 | H | B | 0.118 | 0.15 | **0.732** | **0.5248889** |
| Sequence | 738 | H | E | 0.069 | 0.386 | **0.545** | **0.5233333** |
| Sequence | 739 | F | B | 0.079 | **0.592** | 0.329 | **0.5392222** |
| Sequence | 740 | S | B | 0.079 | **0.592** | 0.329 | **0.5378889** |
| Sequence | 741 | L | B | 0.069 | 0.386 | **0.545** | **0.5333333** |
| Sequence | 742 | S | E | 0.064 | 0.216 | **0.721** | **0.5386667** |
| Sequence | 743 | Q | E | 0.064 | 0.216 | **0.721** | **0.5305556** |
| Sequence | 744 | E | E | 0.056 | 0.142 | **0.802** | **0.536** |
| Sequence | 745 | G | E | 0.052 | 0.084 | **0.864** | **0.5275556** |
| Sequence | 746 | D | E | 0.064 | 0.216 | **0.721** | **0.5221111** |
| Sequence | 747 | T | E | 0.079 | **0.592** | 0.329 | **0.5163333** |
| Sequence | 748 | A | B | 0.021 | **0.756** | 0.223 | **0.5083333** |
| Sequence | 749 | I | E | 0.021 | **0.756** | 0.223 | **0.5073333** |
| Sequence | 750 | L | B | 0.023 | **0.655** | 0.322 | **0.498** |
| Sequence | 751 | D | E | 0.069 | 0.386 | **0.545** | 0.4892222 |
| Sequence | 752 | K | E | 0.064 | 0.216 | **0.721** | 0.481 |
| Sequence | 753 | E | E | 0.064 | 0.216 | **0.721** | 0.4781111 |
| Sequence | 754 | F | E | 0.074 | 0.484 | 0.442 | 0.4767778 |
| Sequence | 755 | L | B | 0.087 | **0.683** | 0.23 | 0.4823333 |
| Sequence | 756 | T | B | 0.087 | **0.683** | 0.23 | 0.4698889 |
| Sequence | 757 | T | B | 0.087 | **0.683** | 0.23 | 0.4433333 |
| Sequence | 758 | T | B | 0.087 | **0.683** | 0.23 | 0.4141111 |
| Sequence | 759 | V | B | 0.021 | **0.756** | 0.223 | 0.4043333 |
| Sequence | 760 | I | B | 0.021 | **0.756** | 0.223 | 0.3965556 |
| Sequence | 761 | G | B | 0.021 | **0.756** | 0.223 | 0.3928889 |
| Sequence | 762 | I | B | 0.021 | **0.756** | 0.223 | 0.4018889 |
| Sequence | 763 | S | B | 0.018 | **0.846** | 0.136 | 0.4131111 |
| Sequence | 764 | G | B | 0.018 | **0.846** | 0.136 | 0.4217778 |
| Sequence | 765 | E | E | 0.018 | **0.846** | 0.136 | 0.4466667 |
| Sequence | 766 | Y | B | 0.023 | **0.655** | 0.322 | 0.4594444 |
| Sequence | 767 | D | E | 0.004 | 0.42 | **0.576** | **0.492** |
| Sequence | 768 | L | B | 0.022 | 0.359 | **0.619** | **0.4965556** |
| Sequence | 769 | D | E | 0.021 | 0.451 | **0.528** | **0.5117778** |
| Sequence | 770 | L | B | 0.022 | 0.359 | **0.619** | **0.527** |
| Sequence | 771 | R | E | 0.022 | 0.359 | **0.619** | **0.5156667** |
| Sequence | 772 | S | E | 0.021 | 0.279 | **0.699** | **0.5134444** |
| Sequence | 773 | K | E | 0.021 | 0.279 | **0.699** | **0.4965556** |
| Sequence | 774 | I | E | 0.021 | 0.451 | **0.528** | 0.4764444 |
| Sequence | 775 | I | B | 0.023 | **0.655** | 0.322 | 0.4691111 |
| Sequence | 776 | K | E | 0.023 | **0.655** | 0.322 | 0.4472222 |
| Sequence | 777 | L | B | 0.079 | **0.592** | 0.329 | 0.4417778 |
| Sequence | 778 | Q | B | 0.079 | **0.592** | 0.329 | 0.4311111 |
| Sequence | 779 | A | B | 0.079 | **0.592** | 0.329 | 0.4245556 |
| Sequence | 780 | S | B | 0.148 | 0.418 | 0.435 | 0.4273333 |
| Sequence | 781 | V | B | 0.148 | 0.418 | 0.435 | 0.4424444 |
| Sequence | 782 | D | E | 0.148 | 0.418 | 0.435 | 0.477 |
| Sequence | 783 | W | B | 0.252 | 0.423 | 0.325 | **0.4913333** |
| Sequence | 784 | I | B | 0.252 | 0.423 | 0.325 | **0.5137778** |
| Sequence | 785 | K | E | 0.231 | 0.33 | 0.439 | **0.532** |
| Sequence | 786 | G | B | 0.216 | 0.235 | **0.548** | **0.5513333** |
| Sequence | 787 | I | B | 0.125 | 0.227 | **0.648** | **0.5621111** |
| Sequence | 788 | S | E | 0.125 | 0.227 | **0.648** | **0.5765556** |
| Sequence | 789 | G | E | 0.199 | 0.152 | **0.649** | **0.5813333** |
| Sequence | 790 | D | E | 0.278 | 0.093 | **0.628** | **0.5626667** |
| Sequence | 791 | F | B | **0.538** | 0.173 | 0.289 | **0.5498889** |
| Sequence | 792 | A | E | **0.649** | 0.163 | 0.188 | **0.5448889** |
| Sequence | 793 | A | B | **0.701** | 0.107 | 0.192 | **0.5372222** |
| Sequence | 794 | K | B | **0.701** | 0.107 | 0.192 | **0.5223333** |
| Sequence | 795 | S | B | **0.779** | 0.1 | 0.12 | **0.523** |
| Sequence | 796 | E | E | **0.779** | 0.1 | 0.12 | **0.5194444** |
| Sequence | 797 | V | B | **0.701** | 0.107 | 0.192 | **0.5187778** |
| Sequence | 798 | L | B | **0.605** | 0.105 | 0.29 | **0.519** |
| Sequence | 799 | N | E | **0.502** | 0.102 | 0.396 | **0.5394444** |
| Sequence | 800 | M | E | 0.386 | 0.097 | **0.517** | **0.5511111** |
| Sequence | 801 | K | E | 0.321 | 0.252 | 0.427 | **0.5647778** |
| Sequence | 802 | F | B | 0.231 | 0.33 | 0.439 | **0.574** |
| Sequence | 803 | K | E | 0.125 | 0.227 | **0.648** | **0.5852222** |
| Sequence | 804 | D | E | 0.064 | 0.216 | **0.721** | **0.5736667** |
| Sequence | 805 | K | E | 0.052 | 0.084 | **0.864** | **0.5638889** |
| Sequence | 806 | N | E | 0.018 | 0.019 | **0.964** | **0.5466667** |
| Sequence | 807 | D | E | 0.058 | 0.017 | **0.925** | **0.5292222** |
| Sequence | 808 | T | E | 0.052 | 0.084 | **0.864** | **0.5091111** |
| Sequence | 809 | S | B | 0.069 | 0.386 | **0.545** | 0.471 |
| Sequence | 810 | T | B | 0.021 | **0.756** | 0.223 | 0.4428889 |
| Sequence | 811 | F | B | 0.018 | **0.846** | 0.136 | 0.4034444 |
| Sequence | 812 | R | B | 0.018 | **0.846** | 0.136 | 0.3815556 |
| Sequence | 813 | G | B | 0.072 | **0.855** | 0.073 | 0.363 |
| Sequence | 814 | T | B | 0.085 | **0.775** | 0.14 | 0.3618889 |
| Sequence | 815 | L | B | 0.159 | **0.516** | 0.325 | 0.3601111 |
| Sequence | 816 | G | B | 0.216 | 0.235 | **0.548** | 0.3743333 |
| Sequence | 817 | A | B | 0.278 | 0.093 | **0.628** | 0.3558889 |
| Sequence | 818 | S | B | 0.386 | 0.097 | **0.517** | 0.3791111 |
| Sequence | 819 | A | B | **0.649** | 0.163 | 0.188 | 0.3956667 |
| Sequence | 820 | Q | B | **0.725** | 0.163 | 0.112 | 0.4221111 |
| Sequence | 821 | L | B | **0.779** | 0.1 | 0.12 | 0.4294444 |
| Sequence | 822 | L | B | **0.751** | 0.05 | 0.199 | 0.4493333 |
| Sequence | 823 | E | E | **0.66** | 0.049 | 0.291 | 0.4343333 |
| Sequence | 824 | N | B | 0.455 | 0.046 | **0.498** | 0.4412222 |
| Sequence | 825 | F | B | 0.199 | 0.152 | **0.649** | 0.4213333 |
| Sequence | 826 | E | E | 0.148 | 0.418 | 0.435 | 0.4263333 |
| Sequence | 827 | V | B | 0.079 | **0.592** | 0.329 | 0.4008889 |
| Sequence | 828 | H | B | 0.079 | **0.592** | 0.329 | 0.3922222 |
| Sequence | 829 | L | B | 0.079 | **0.592** | 0.329 | 0.3806667 |
| Sequence | 830 | D | B | 0.169 | **0.612** | 0.219 | 0.3897778 |
| Sequence | 831 | I | B | 0.169 | **0.612** | 0.219 | 0.402 |
| Sequence | 832 | F | B | 0.159 | **0.516** | 0.325 | 0.4298889 |
| Sequence | 833 | G | B | 0.135 | 0.317 | **0.548** | 0.4537778 |
| Sequence | 834 | D | E | 0.118 | 0.15 | **0.732** | **0.5017778** |
| Sequence | 835 | L | E | 0.118 | 0.15 | **0.732** | **0.5387778** |
| Sequence | 836 | G | B | 0.113 | 0.087 | **0.8** | **0.5741111** |
| Sequence | 837 | N | E | 0.053 | 0.043 | **0.903** | **0.5793333** |
| Sequence | 838 | D | E | 0.053 | 0.043 | **0.903** | **0.5995556** |
| Sequence | 839 | K | E | 0.053 | 0.043 | **0.903** | **0.6092222** |
| Sequence | 840 | G | B | 0.056 | 0.142 | **0.802** | **0.6116667** |
| Sequence | 841 | I | B | 0.064 | 0.216 | **0.721** | **0.6126667** |
| Sequence | 842 | G | B | 0.02 | 0.205 | **0.775** | **0.5985556** |
| Sequence | 843 | G | B | 0.021 | 0.279 | **0.699** | **0.5831111** |
| Sequence | 844 | Q | E | 0.021 | 0.451 | **0.528** | **0.5643333** |
| Sequence | 845 | V | B | 0.022 | 0.359 | **0.619** | **0.5572222** |
| Sequence | 846 | G | E | 0.022 | 0.359 | **0.619** | **0.5595556** |
| Sequence | 847 | A | B | 0.022 | **0.552** | 0.426 | **0.5507778** |
| Sequence | 848 | T | E | 0.021 | **0.756** | 0.223 | 0.4838889 |
| Sequence | 849 | Y | B | 0.003 | **0.718** | 0.279 | 0.413 |
| Sequence | 850 | R | E | 0.004 | **0.514** | 0.481 | 0.3478889 |
| Sequence | 851 | F | E | 0.016 | 0.005 | **0.979** | 0.2838889 |
| All predictions above 0.49 are colored in bold font for probabilityof helix/sheet, loop, and B-cell antigenicity, respectively. | | | | | | | |

**S8 Table:** B-cell epitope prediction, probability of being a helix, loop, or sheet for Q1MRS4 through BepiPred - 2.0.

| **Entry** | **Position** | **AminoAcid** | **Exposed/Buried** | **Helix** | **Sheet** | **Coil** | **Epitope** |
| --- | --- | --- | --- | --- | --- | --- | --- |
| Sequence | 1 | M | E | 0.003 | 0.003 | **0.994** | 0.220778 |
| Sequence | 2 | H | E | 0.184 | 0.043 | **0.773** | 0.267778 |
| Sequence | 3 | E | B | **0.802** | 0.014 | 0.185 | 0.292889 |
| Sequence | 4 | M | B | **0.938** | 0.007 | 0.055 | 0.341889 |
| Sequence | 5 | S | B | **0.938** | 0.007 | 0.055 | 0.385556 |
| Sequence | 6 | L | B | **0.975** | 0.003 | 0.022 | 0.391556 |
| Sequence | 7 | V | B | **0.975** | 0.003 | 0.022 | 0.391333 |
| Sequence | 8 | T | B | **0.97** | 0.001 | 0.03 | 0.393667 |
| Sequence | 9 | G | B | **0.988** | 0 | 0.012 | 0.401 |
| Sequence | 10 | I | B | **0.988** | 0 | 0.012 | 0.372556 |
| Sequence | 11 | L | B | **0.988** | 0 | 0.012 | 0.382444 |
| Sequence | 12 | S | E | **0.988** | 0 | 0.012 | 0.406222 |
| Sequence | 13 | I | B | **0.988** | 0 | 0.012 | 0.408556 |
| Sequence | 14 | I | B | **0.97** | 0.001 | 0.03 | 0.398222 |
| Sequence | 15 | Q | E | **0.988** | 0 | 0.012 | 0.424778 |
| Sequence | 16 | E | E | **0.97** | 0.001 | 0.03 | 0.450556 |
| Sequence | 17 | E | B | **0.97** | 0.001 | 0.03 | 0.466556 |
| Sequence | 18 | M | B | **0.97** | 0.001 | 0.03 | 0.459778 |
| Sequence | 19 | S | E | **0.923** | 0.002 | 0.076 | 0.473778 |
| Sequence | 20 | K | E | **0.782** | 0.003 | 0.216 | 0.48 |
| Sequence | 21 | N | E | 0.321 | 0.003 | **0.675** | **0.499778** |
| Sequence | 22 | G | E | 0.016 | 0.005 | **0.979** | **0.495889** |
| Sequence | 23 | V | B | 0.005 | 0.015 | **0.979** | **0.518889** |
| Sequence | 24 | N | E | 0.005 | 0.262 | **0.733** | **0.506333** |
| Sequence | 25 | K | E | 0.003 | **0.718** | 0.279 | 0.476889 |
| Sequence | 26 | L | B | 0.001 | **0.9** | 0.099 | 0.454111 |
| Sequence | 27 | Q | E | 0.001 | **0.9** | 0.099 | 0.432778 |
| Sequence | 28 | R | E | 0.001 | **0.959** | 0.04 | 0.443444 |
| Sequence | 29 | V | B | 0.001 | **0.959** | 0.04 | 0.421222 |
| Sequence | 30 | K | B | 0 | **0.983** | 0.017 | 0.398778 |
| Sequence | 31 | V | B | 0 | **0.983** | 0.017 | 0.415222 |
| Sequence | 32 | C | E | 0.001 | **0.959** | 0.04 | 0.413778 |
| Sequence | 33 | Y | B | 0.011 | **0.918** | 0.071 | 0.431222 |
| Sequence | 34 | G | B | 0.022 | **0.552** | 0.426 | 0.465222 |
| Sequence | 35 | E | E | 0.022 | 0.359 | **0.619** | 0.471333 |
| Sequence | 36 | L | B | 0.066 | 0.296 | **0.638** | **0.5** |
| Sequence | 37 | T | B | 0.056 | 0.142 | **0.802** | **0.501111** |
| Sequence | 38 | N | E | 0.018 | 0.088 | **0.893** | **0.514889** |
| Sequence | 39 | I | B | 0.018 | 0.088 | **0.893** | **0.519333** |
| Sequence | 40 | V | E | 0.053 | 0.043 | **0.903** | **0.499222** |
| Sequence | 41 | P | E | **0.923** | 0.002 | 0.076 | **0.498** |
| Sequence | 42 | D | E | **0.97** | 0.001 | 0.03 | **0.490222** |
| Sequence | 43 | S | B | **0.97** | 0.001 | 0.03 | 0.446111 |
| Sequence | 44 | L | B | **0.988** | 0 | 0.012 | 0.434667 |
| Sequence | 45 | Q | E | **0.97** | 0.001 | 0.03 | 0.431111 |
| Sequence | 46 | F | B | **0.97** | 0.001 | 0.03 | 0.434333 |
| Sequence | 47 | A | B | **0.97** | 0.001 | 0.03 | 0.417556 |
| Sequence | 48 | F | B | **0.97** | 0.001 | 0.03 | 0.422889 |
| Sequence | 49 | K | E | **0.97** | 0.001 | 0.03 | 0.444222 |
| Sequence | 50 | I | B | **0.97** | 0.001 | 0.03 | 0.446222 |
| Sequence | 51 | F | B | **0.923** | 0.002 | 0.076 | 0.448444 |
| Sequence | 52 | T | B | **0.802** | 0.014 | 0.185 | 0.483556 |
| Sequence | 53 | E | E | 0.43 | 0.016 | **0.555** | **0.494556** |
| Sequence | 54 | G | E | 0.115 | 0.016 | **0.868** | **0.507556** |
| Sequence | 55 | T | B | 0.053 | 0.043 | **0.903** | **0.507333** |
| Sequence | 56 | S | B | 0.113 | 0.087 | **0.8** | **0.518556** |
| Sequence | 57 | L | B | 0.113 | 0.087 | **0.8** | **0.514667** |
| Sequence | 58 | E | E | 0.052 | 0.084 | **0.864** | **0.496778** |
| Sequence | 59 | G | E | 0.018 | 0.088 | **0.893** | **0.490667** |
| Sequence | 60 | A | B | 0.022 | 0.359 | **0.619** | 0.477 |
| Sequence | 61 | I | E | 0.002 | **0.816** | 0.182 | 0.471556 |
| Sequence | 62 | L | B | 0.001 | **0.9** | 0.099 | 0.481778 |
| Sequence | 63 | E | E | 0.001 | **0.959** | 0.04 | 0.467778 |
| Sequence | 64 | I | B | 0.001 | **0.959** | 0.04 | 0.465222 |
| Sequence | 65 | E | E | 0.001 | **0.9** | 0.099 | 0.464556 |
| Sequence | 66 | K | E | 0.002 | **0.816** | 0.182 | 0.466222 |
| Sequence | 67 | I | E | 0.003 | **0.718** | 0.279 | 0.452778 |
| Sequence | 68 | P | E | 0.021 | 0.279 | **0.699** | 0.451333 |
| Sequence | 69 | L | B | 0.021 | 0.451 | **0.528** | 0.435222 |
| Sequence | 70 | M | E | 0.011 | **0.918** | 0.071 | 0.431667 |
| Sequence | 71 | L | B | 0.001 | **0.959** | 0.04 | 0.428889 |
| Sequence | 72 | R | E | 0.001 | **0.959** | 0.04 | 0.425889 |
| Sequence | 73 | C | B | 0.021 | **0.756** | 0.223 | 0.437 |
| Sequence | 74 | S | E | 0.02 | 0.205 | **0.775** | 0.449222 |
| Sequence | 75 | N | E | 0.018 | 0.047 | **0.935** | 0.449111 |
| Sequence | 76 | C | B | 0.018 | 0.019 | **0.964** | 0.475 |
| Sequence | 77 | L | E | 0.005 | 0.015 | **0.979** | 0.483444 |
| Sequence | 78 | S | E | 0.004 | 0.197 | **0.799** | **0.507111** |
| Sequence | 79 | L | E | 0.004 | **0.514** | 0.481 | **0.511333** |
| Sequence | 80 | F | B | 0.004 | **0.616** | 0.381 | **0.521222** |
| Sequence | 81 | T | E | 0.004 | **0.514** | 0.481 | **0.545556** |
| Sequence | 82 | P | B | 0.022 | 0.359 | **0.619** | **0.554111** |
| Sequence | 83 | E | E | 0.02 | 0.205 | **0.775** | **0.566333** |
| Sequence | 84 | D | E | 0.02 | 0.205 | **0.775** | **0.581111** |
| Sequence | 85 | K | E | 0.056 | 0.142 | **0.802** | **0.592** |
| Sequence | 86 | Q | E | 0.064 | 0.216 | **0.721** | **0.599** |
| Sequence | 87 | K | E | 0.022 | 0.359 | **0.619** | **0.600889** |
| Sequence | 88 | I | E | 0.022 | 0.359 | **0.619** | **0.598111** |
| Sequence | 89 | F | E | 0.021 | 0.451 | **0.528** | **0.571444** |
| Sequence | 90 | F | E | 0.022 | **0.552** | 0.426 | **0.555889** |
| Sequence | 91 | I | B | 0.004 | **0.514** | 0.481 | **0.541333** |
| Sequence | 92 | T | E | 0.004 | 0.42 | **0.576** | **0.510333** |
| Sequence | 93 | C | B | 0.004 | 0.085 | **0.91** | **0.507333** |
| Sequence | 94 | P | E | 0.018 | 0.019 | **0.964** | **0.506333** |
| Sequence | 95 | S | E | 0.018 | 0.047 | **0.935** | **0.509222** |
| Sequence | 96 | C | B | 0.018 | 0.019 | **0.964** | **0.517556** |
| Sequence | 97 | K | E | 0.005 | 0.015 | **0.979** | **0.531444** |
| Sequence | 98 | K | E | 0.005 | 0.015 | **0.979** | **0.544556** |
| Sequence | 99 | E | E | 0.018 | 0.088 | **0.893** | **0.532889** |
| Sequence | 100 | V | B | 0.004 | 0.138 | **0.858** | **0.525111** |
| Sequence | 101 | A | E | 0.005 | 0.262 | **0.733** | **0.541889** |
| Sequence | 102 | Y | B | 0.004 | **0.616** | 0.381 | **0.537111** |
| Sequence | 103 | N | E | 0.002 | **0.816** | 0.182 | **0.521111** |
| Sequence | 104 | V | B | 0.001 | **0.9** | 0.099 | **0.512111** |
| Sequence | 105 | E | E | 0.003 | **0.718** | 0.279 | **0.511667** |
| Sequence | 106 | T | E | 0.005 | 0.262 | **0.733** | 0.487556 |
| Sequence | 107 | G | E | 0.005 | 0.045 | **0.951** | 0.492222 |
| Sequence | 108 | R | E | 0.004 | 0.138 | **0.858** | 0.488889 |
| Sequence | 109 | E | E | 0.004 | **0.616** | 0.381 | **0.496556** |
| Sequence | 110 | F | B | 0.001 | **0.9** | 0.099 | **0.495222** |
| Sequence | 111 | Y | E | 0.001 | **0.959** | 0.04 | 0.487889 |
| Sequence | 112 | I | B | 0.001 | **0.959** | 0.04 | 0.487556 |
| Sequence | 113 | Q | E | 0.001 | **0.959** | 0.04 | 0.478 |
| Sequence | 114 | H | E | 0.001 | **0.959** | 0.04 | 0.473889 |
| Sequence | 115 | L | B | 0.001 | **0.959** | 0.04 | 0.429444 |
| Sequence | 116 | E | E | 0.001 | **0.9** | 0.099 | 0.371111 |
| Sequence | 117 | V | B | 0.003 | **0.718** | 0.279 | 0.326556 |
| Sequence | 118 | E | E | 0.005 | 0.015 | **0.979** | 0.269111 |
| All predictions above 0.49 are colored in bold font for probabilityof helix/sheet, loop, and B-cell antigenicity, respectively. | | | | | | | |
